# Supplementary material for: Loss function of tumor suppressor FRMD8 confers resistance to tamoxifen therapy via a dual mechanism
Source: eLife. 2025 Apr 11;13:RP101888. doi: 10.7554/eLife.101888 (PMC11991697; doi:10.7554/eLife.101888)

Figure 1-source data: Unedited gel pictures for figure 1.

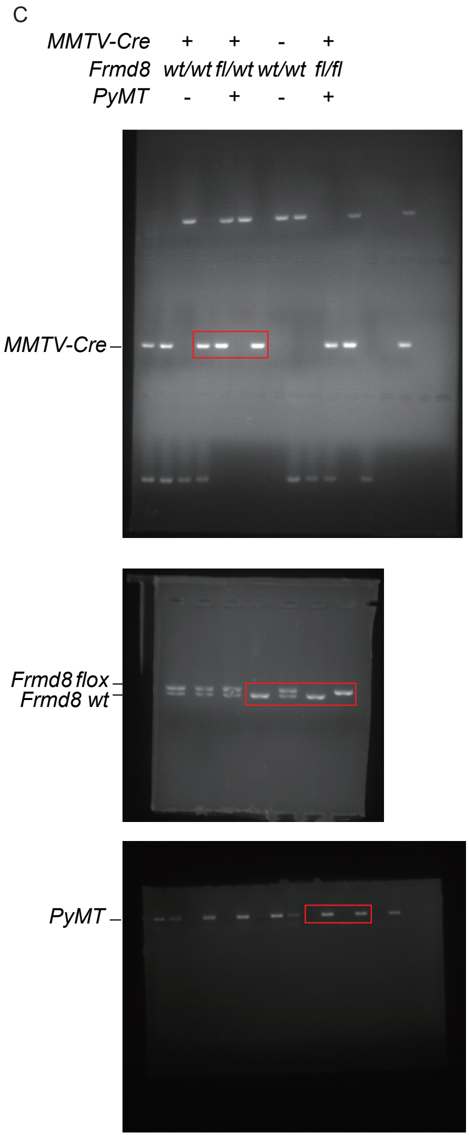

Supplement: Figure 1—source data 1. [file elife-101888-fig1-data1.pdf]
