# Supplementary figures and images for: Loss function of tumor suppressor FRMD8 confers resistance to tamoxifen therapy via a dual mechanism
Source: eLife. 2025 Apr 11;13:RP101888. doi: 10.7554/eLife.101888 (PMC11991697; doi:10.7554/eLife.101888)

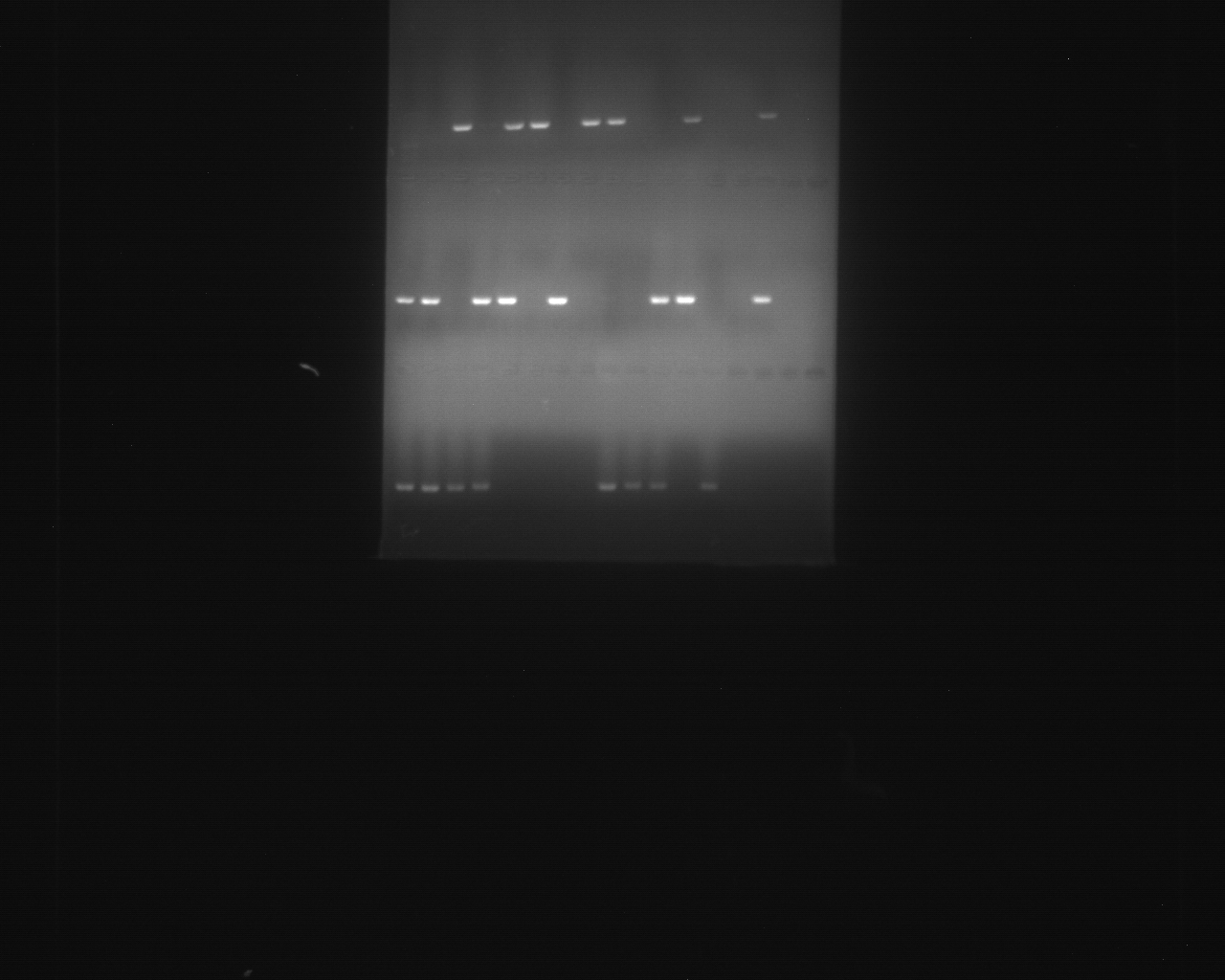

Supplement: Figure 1—source data 2. [file elife-101888-fig1-data2.zip › Figure 1C/Cre.bmp]

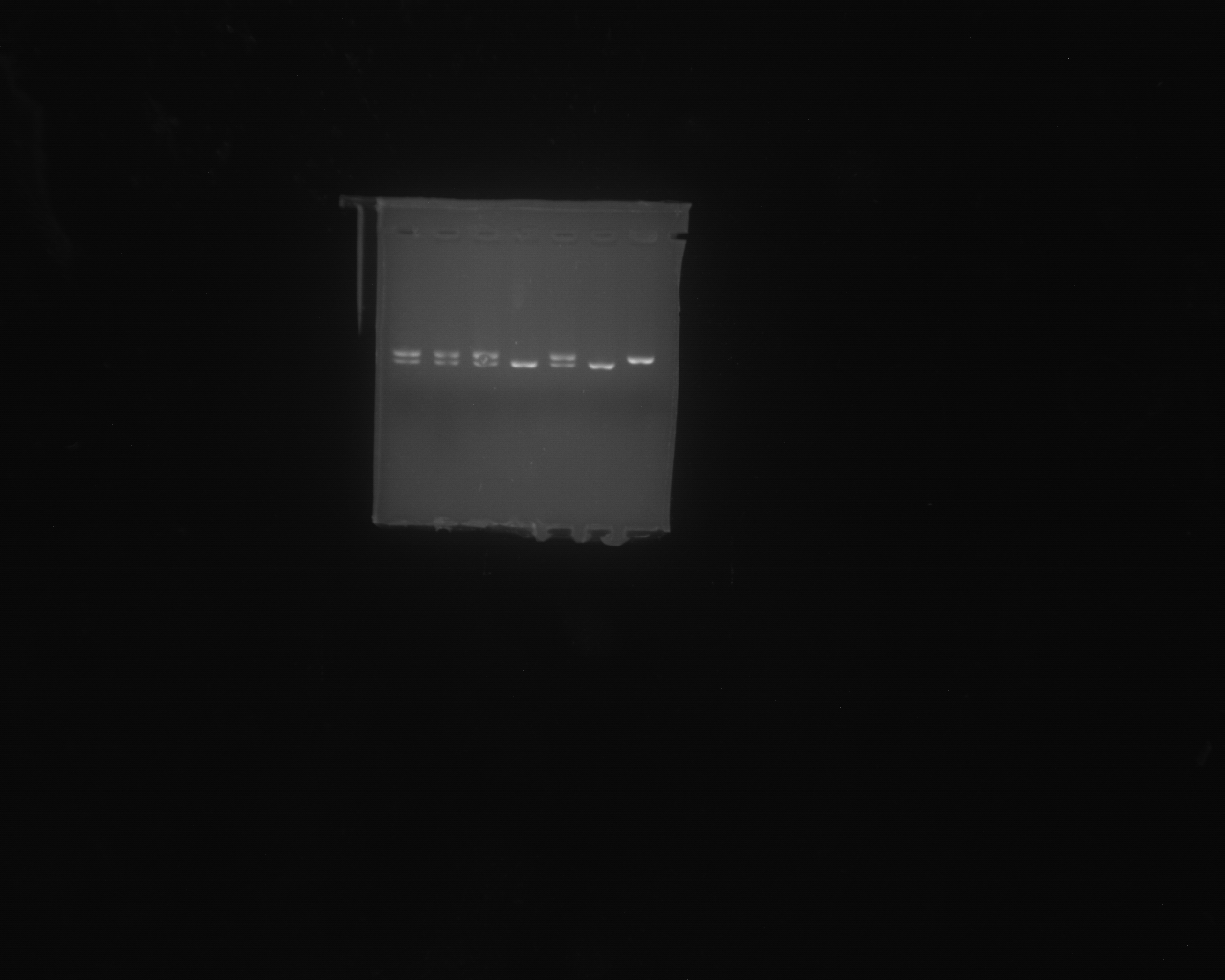

Supplement: Figure 1—source data 2. [file elife-101888-fig1-data2.zip › Figure 1C/Frmd8ecKO.bmp]

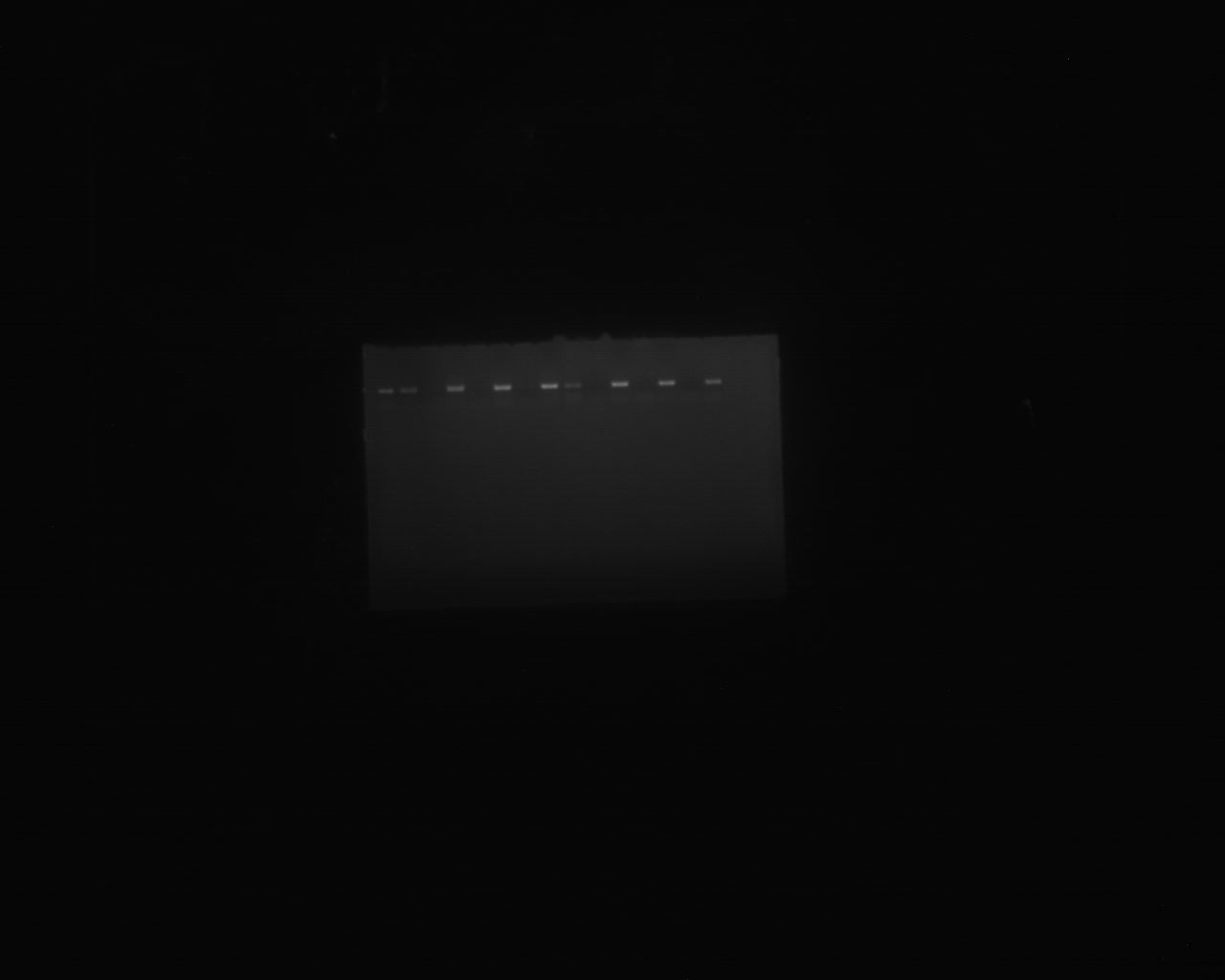

Supplement: Figure 1—source data 2. [file elife-101888-fig1-data2.zip › Figure 1C/PyMT.bmp]

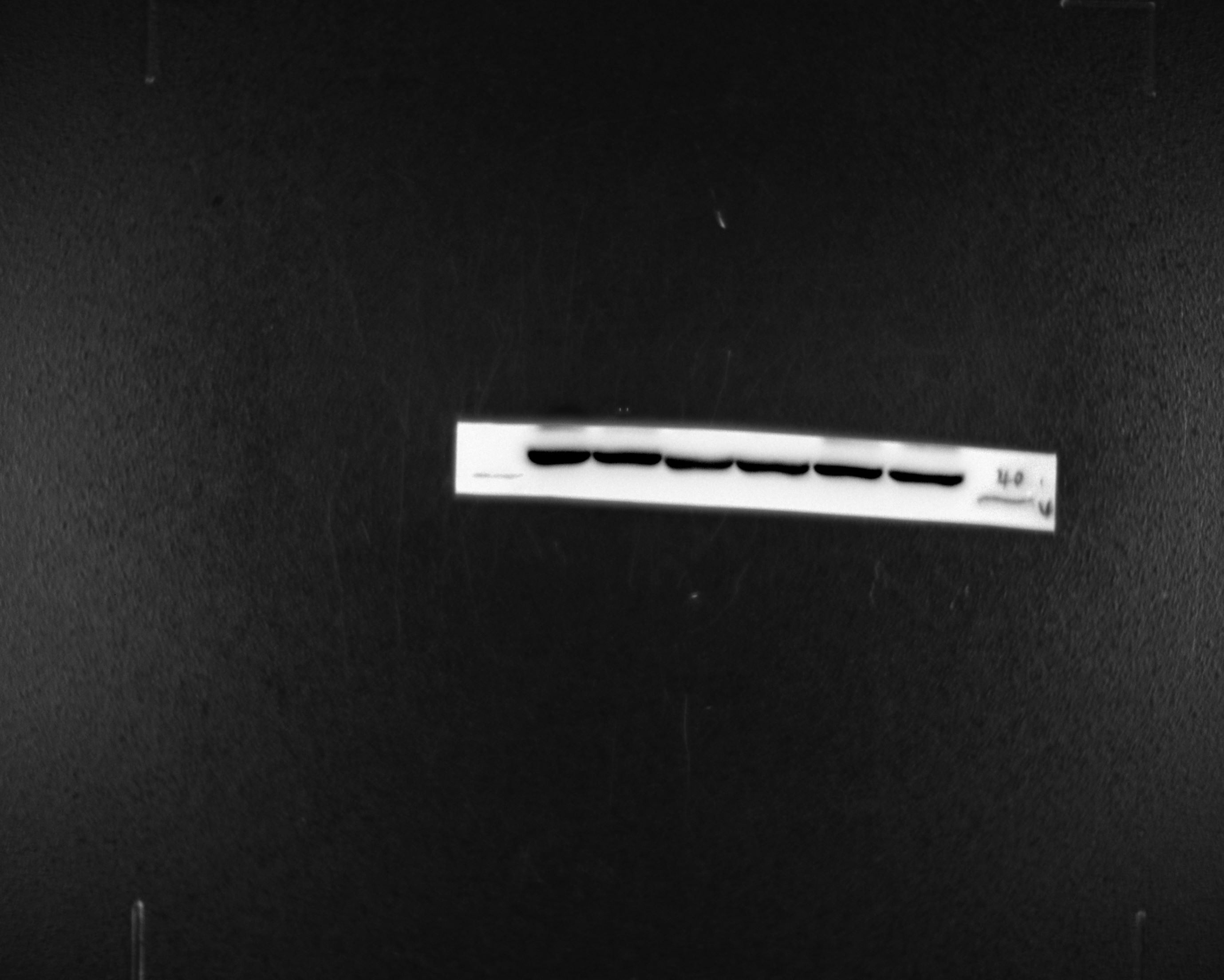

Supplement: Figure 1—figure supplement 1—source data 2. [file elife-101888-fig1-figsupp1-data2.zip › Figure 1– figure supplement 1B/Actin.jpg]

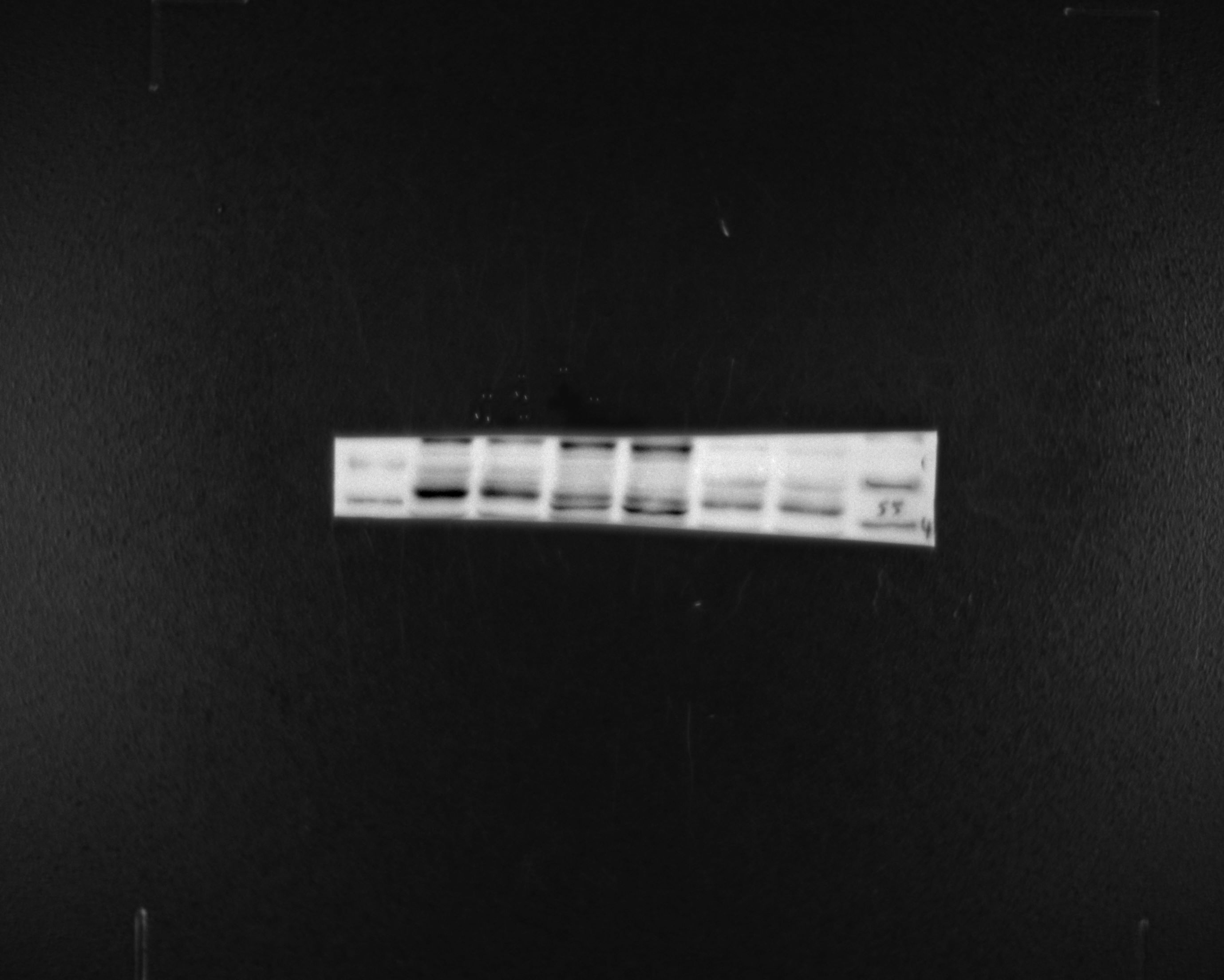

Supplement: Figure 1—figure supplement 1—source data 2. [file elife-101888-fig1-figsupp1-data2.zip › Figure 1– figure supplement 1B/Frmd8.jpg]

Figure 3-source data: Unedited western blot pictures for figure 3.

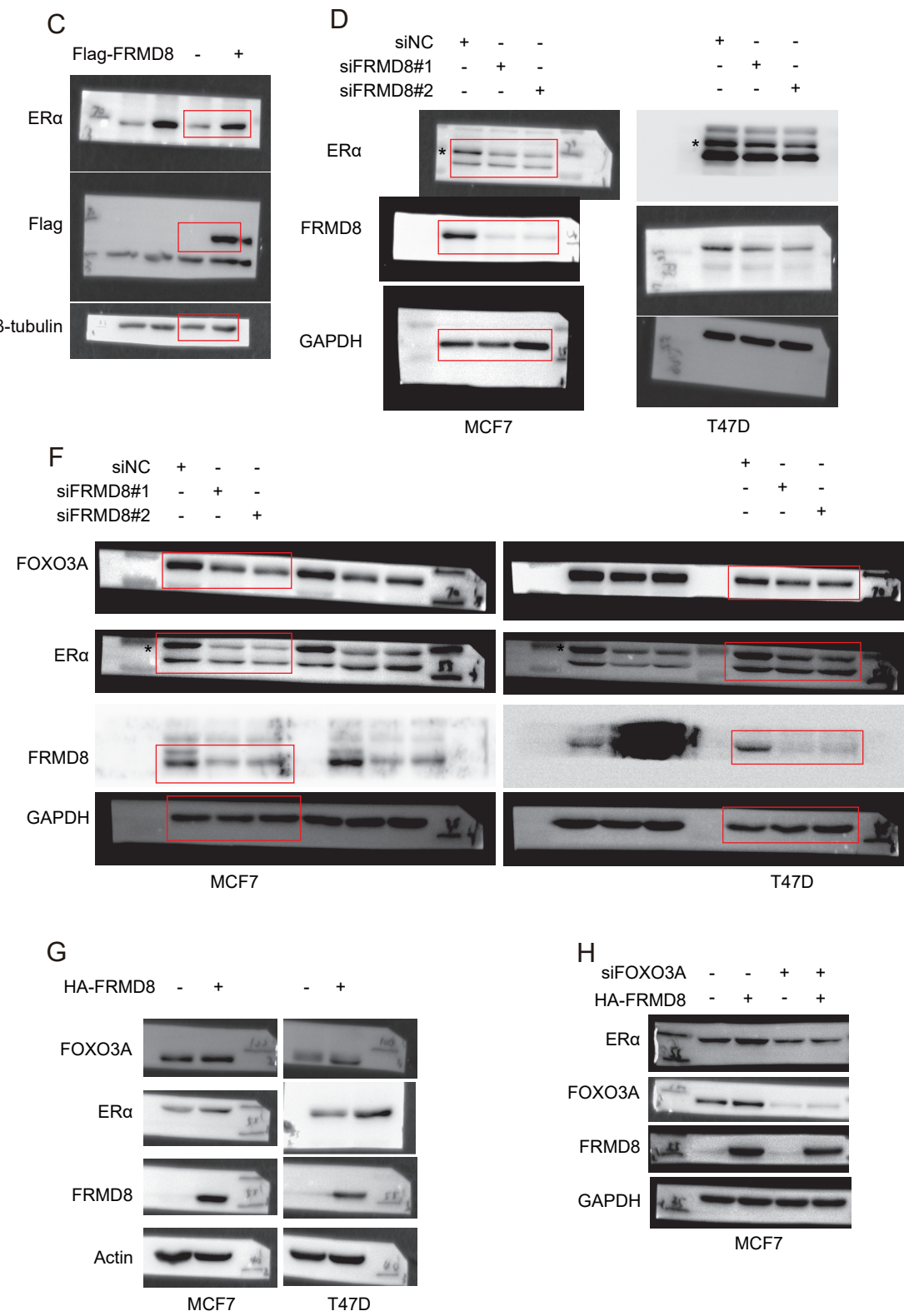

Supplement: Figure 3—source data 1. [file elife-101888-fig3-data1.pdf]

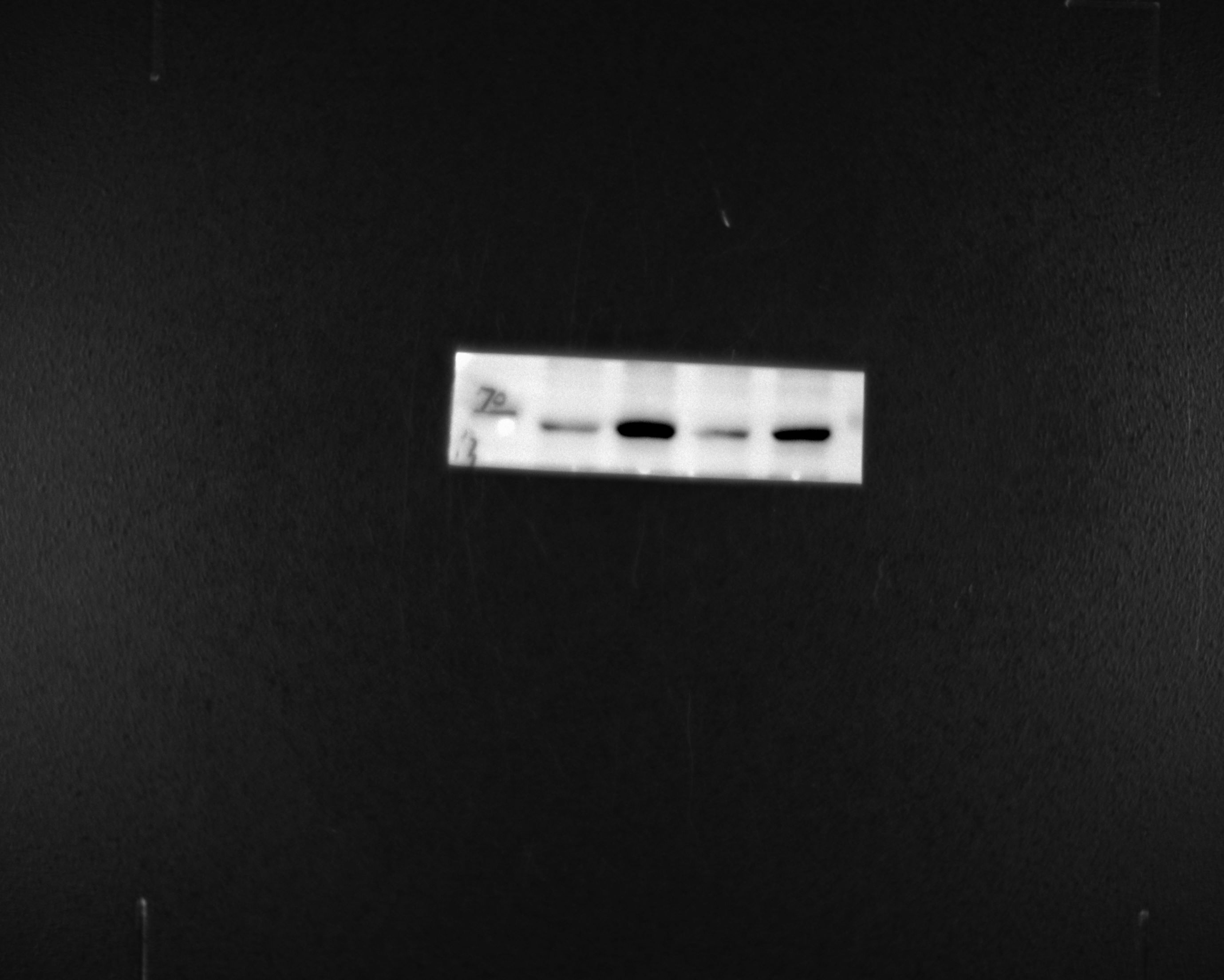

Supplement: Figure 3—source data 2. [file elife-101888-fig3-data2.zip › Figure 3C/ERα.jpg]

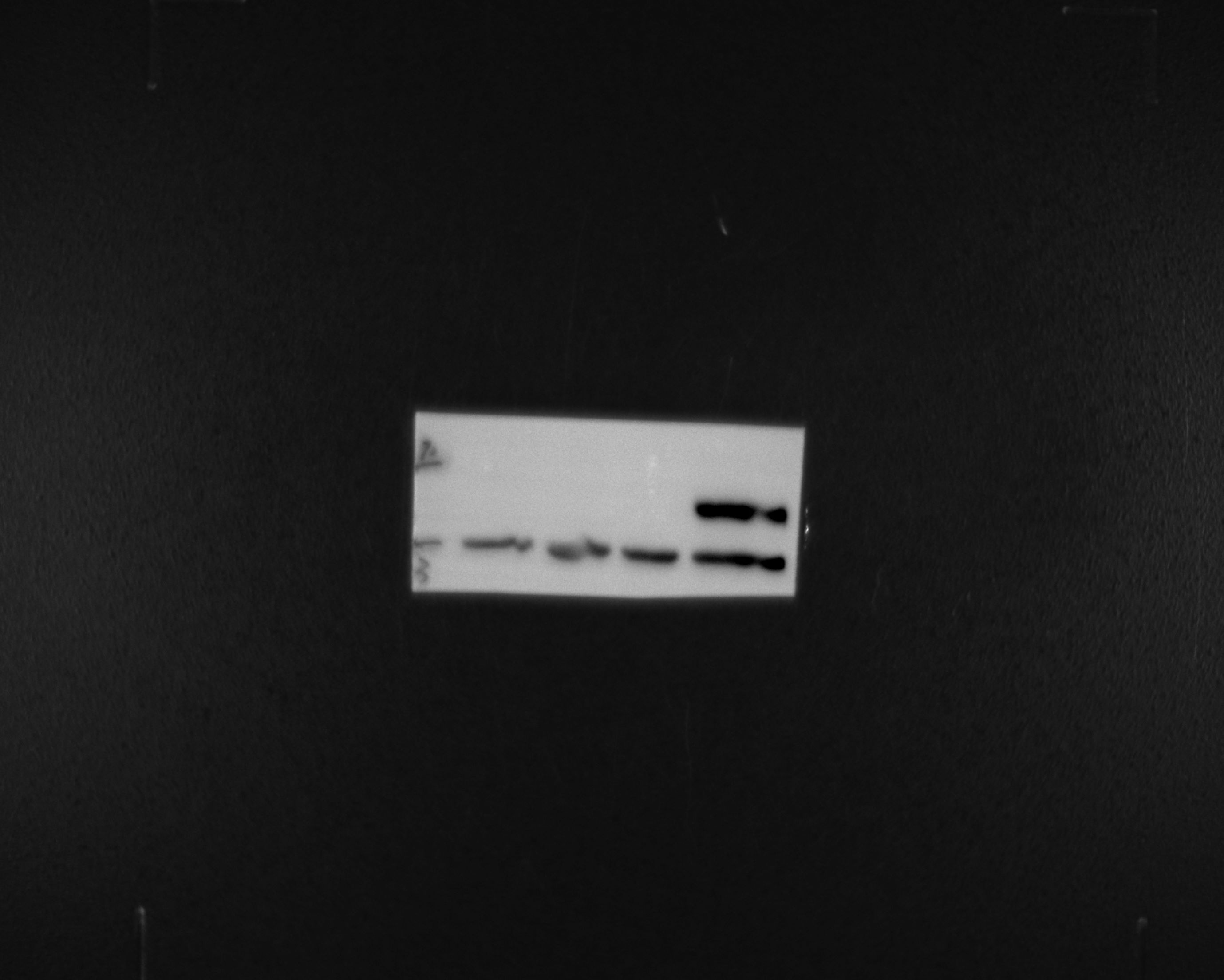

Supplement: Figure 3—source data 2. [file elife-101888-fig3-data2.zip › Figure 3C/Flag.jpg]

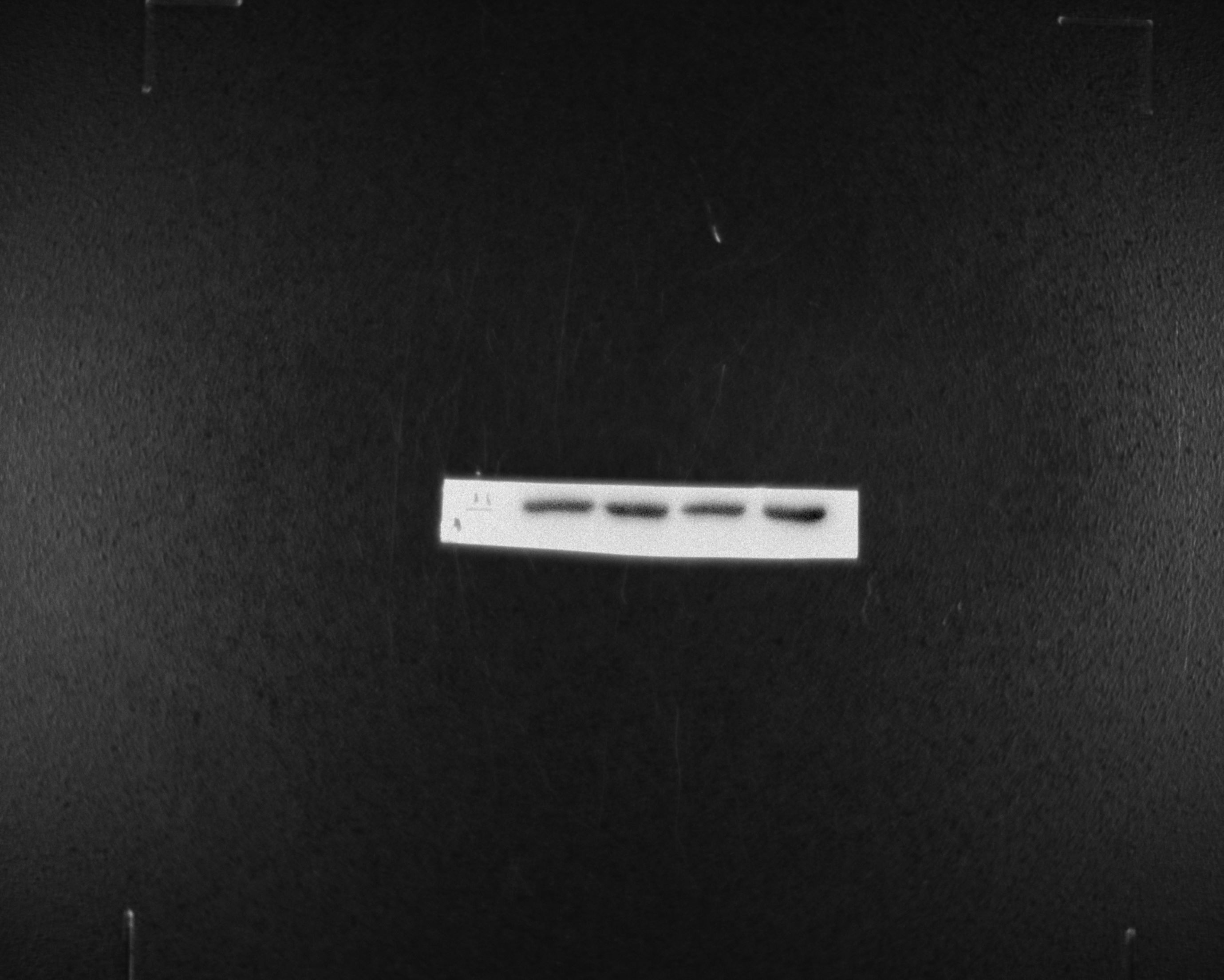

Supplement: Figure 3—source data 2. [file elife-101888-fig3-data2.zip › Figure 3C/β-tubulin.jpg]

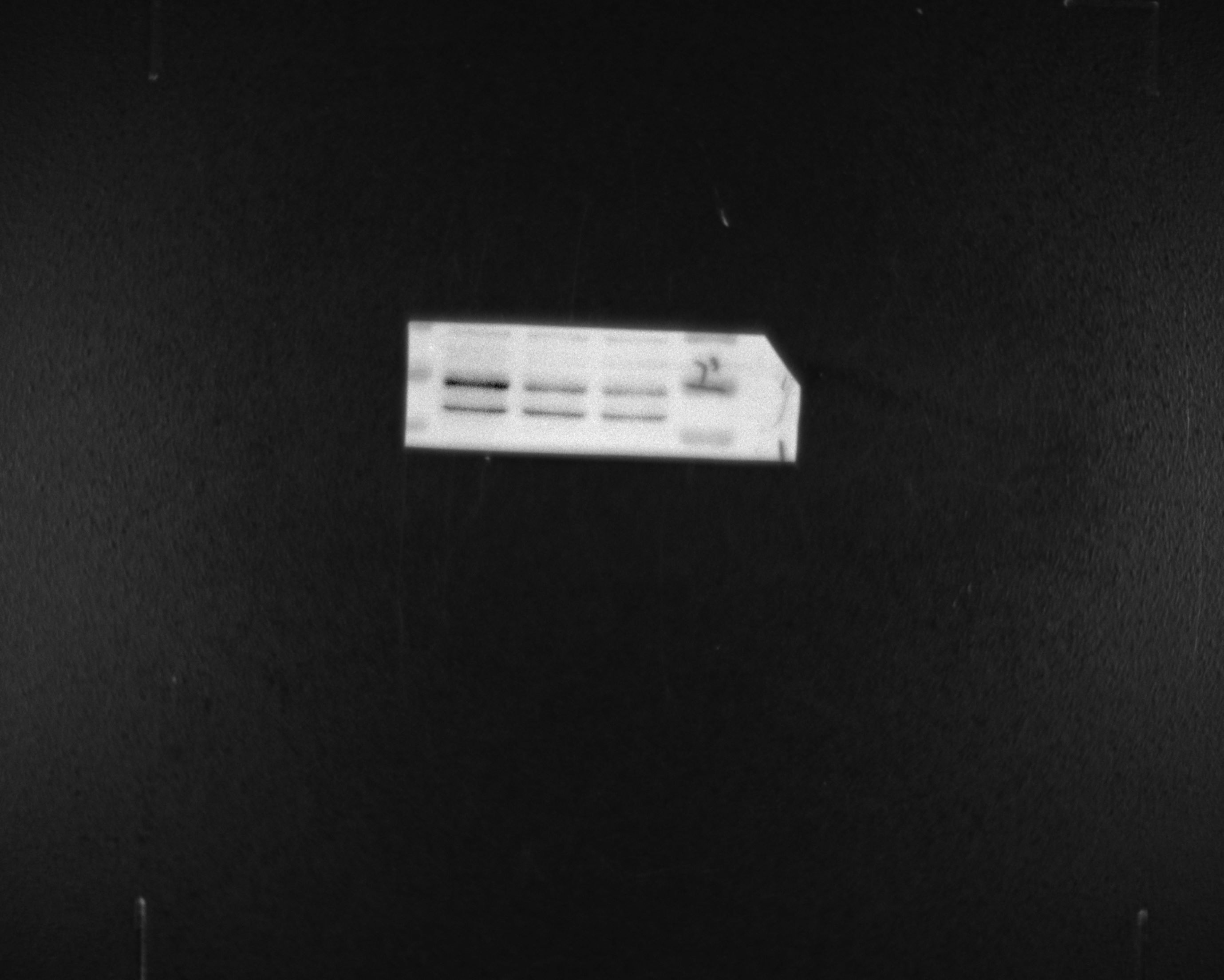

Supplement: Figure 3—source data 2. [file elife-101888-fig3-data2.zip › Figure 3D/MCF7/ERα.jpg]

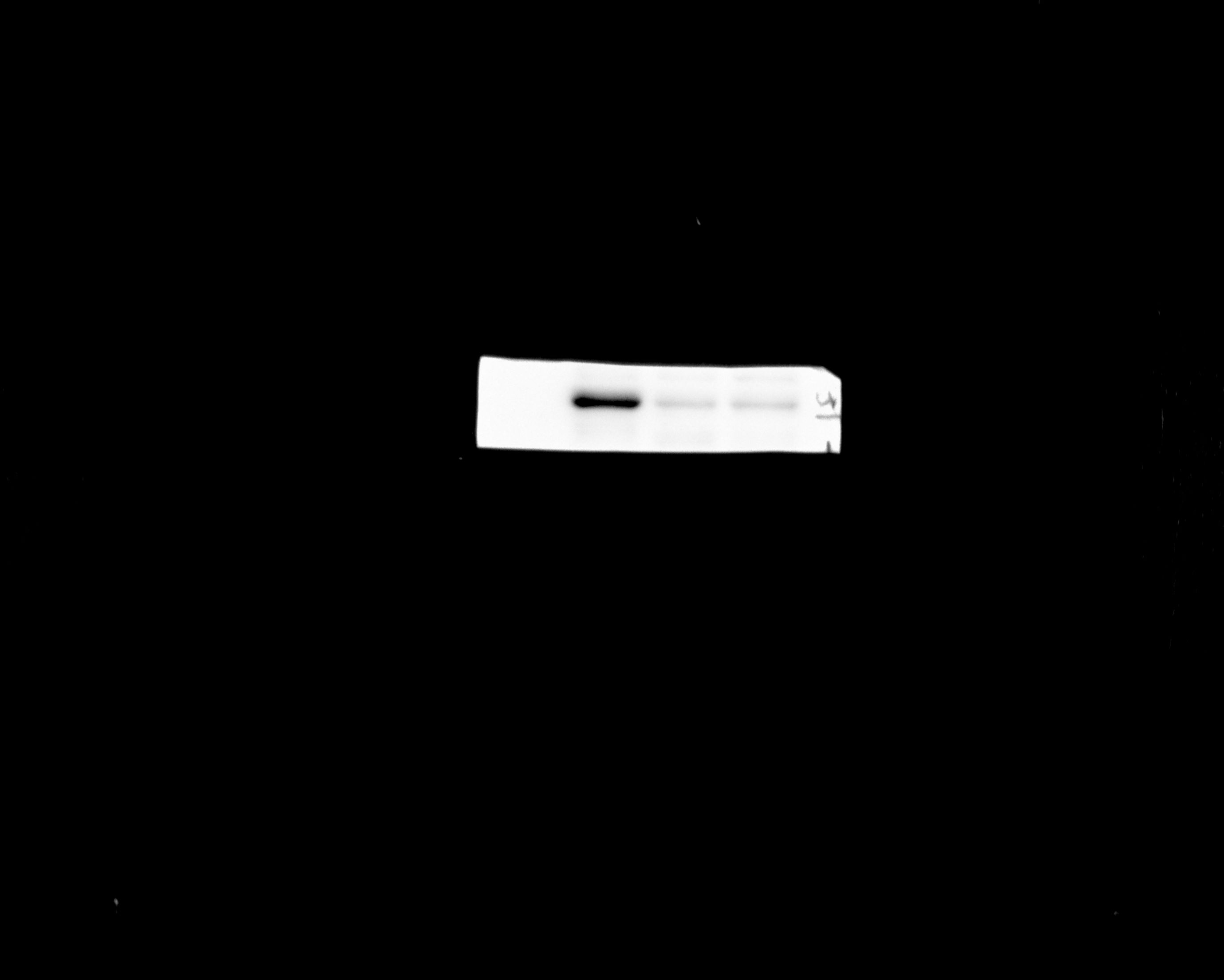

Supplement: Figure 3—source data 2. [file elife-101888-fig3-data2.zip › Figure 3D/MCF7/FRMD8.jpg]

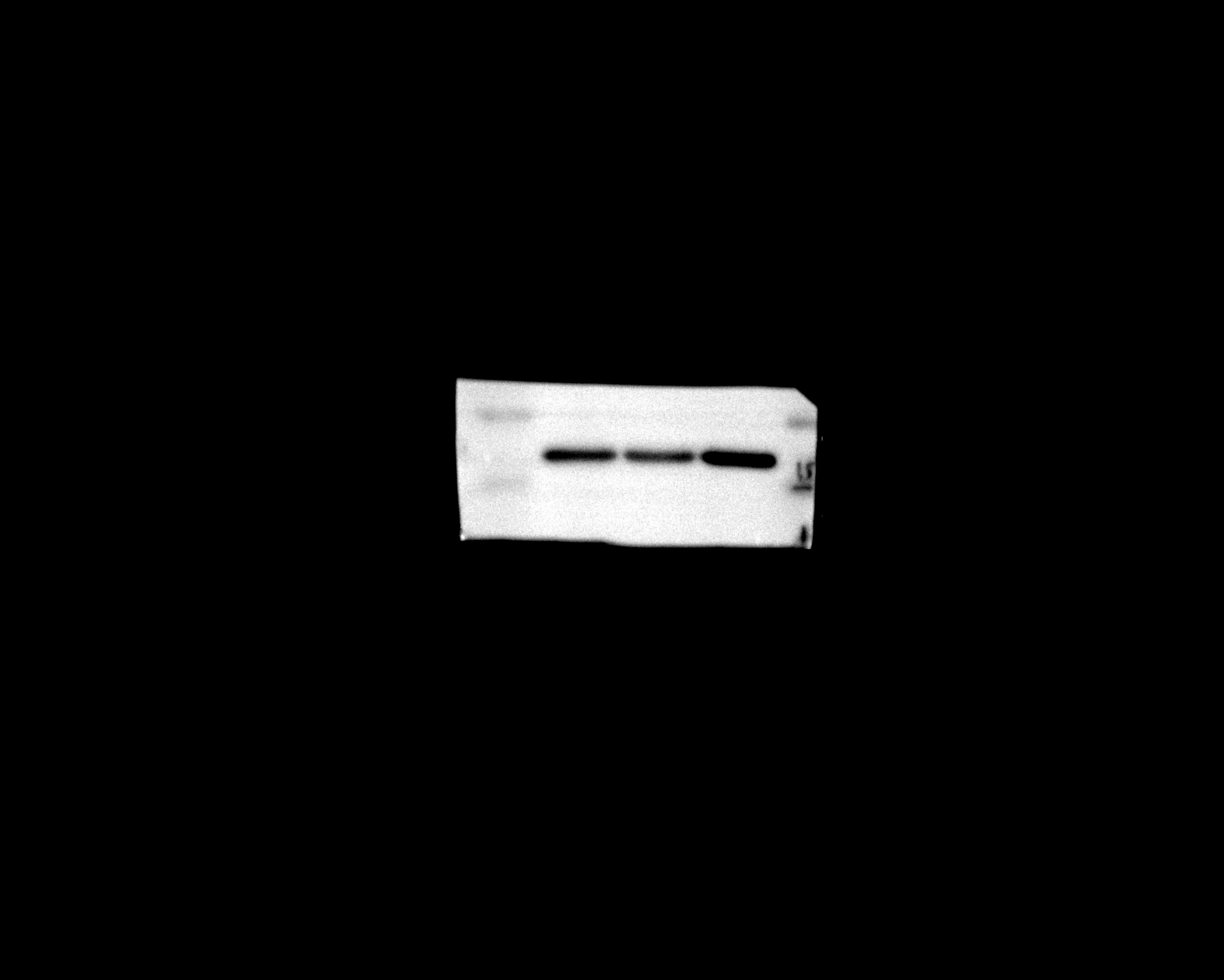

Supplement: Figure 3—source data 2. [file elife-101888-fig3-data2.zip › Figure 3D/MCF7/GAPDH.jpg]

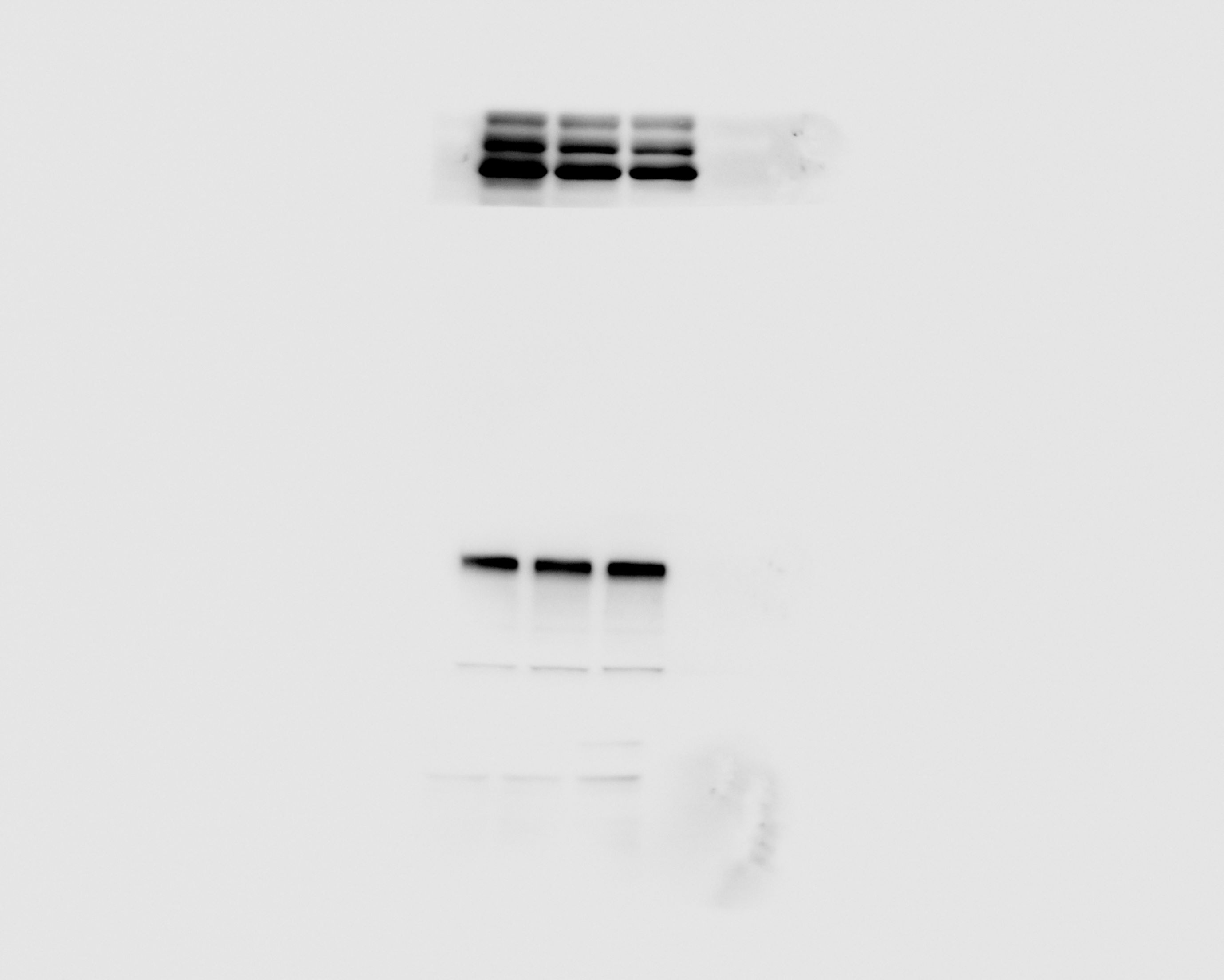

Supplement: Figure 3—source data 2. [file elife-101888-fig3-data2.zip › Figure 3D/T47D/ERα.jpg]

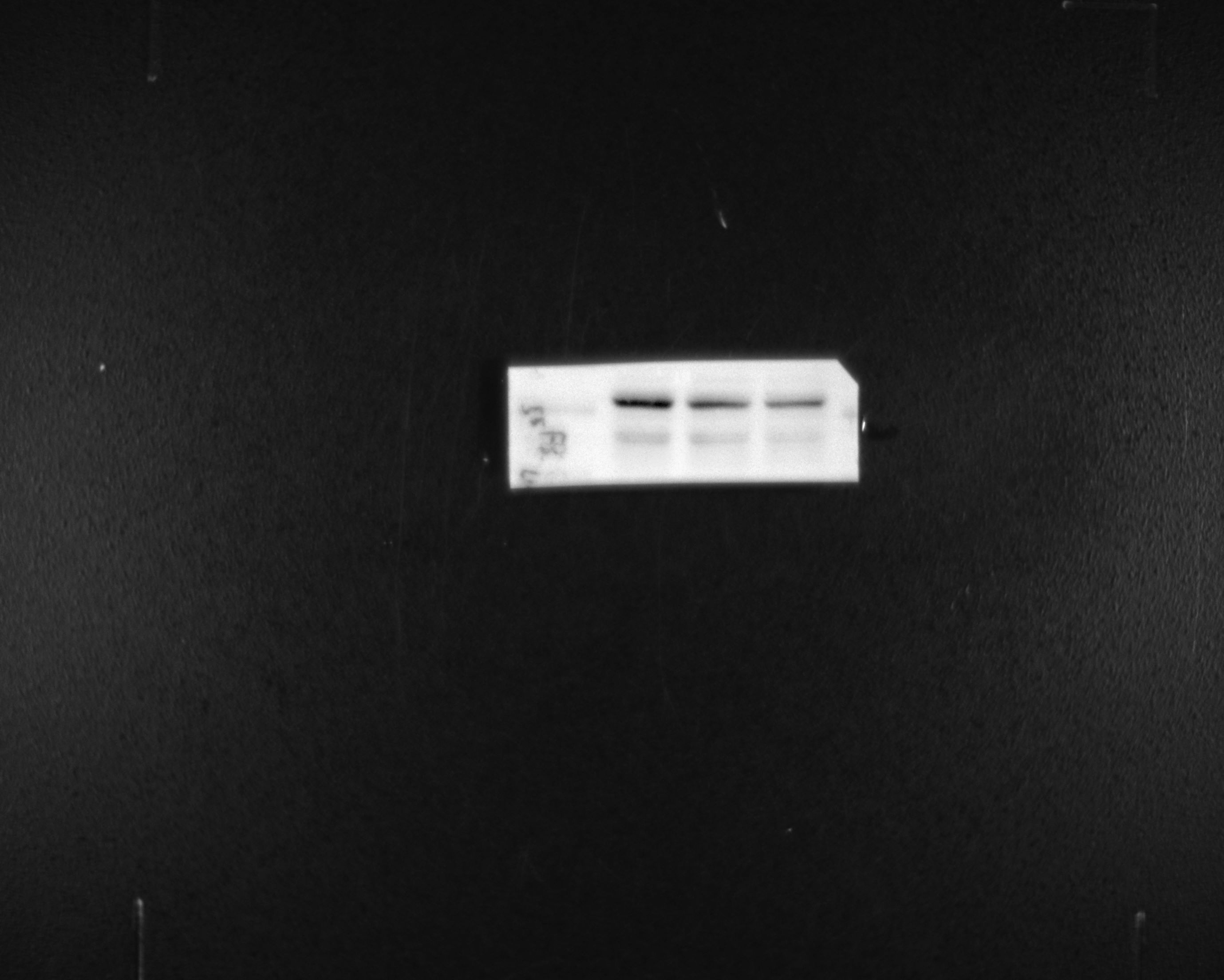

Supplement: Figure 3—source data 2. [file elife-101888-fig3-data2.zip › Figure 3D/T47D/FRMD8.jpg]

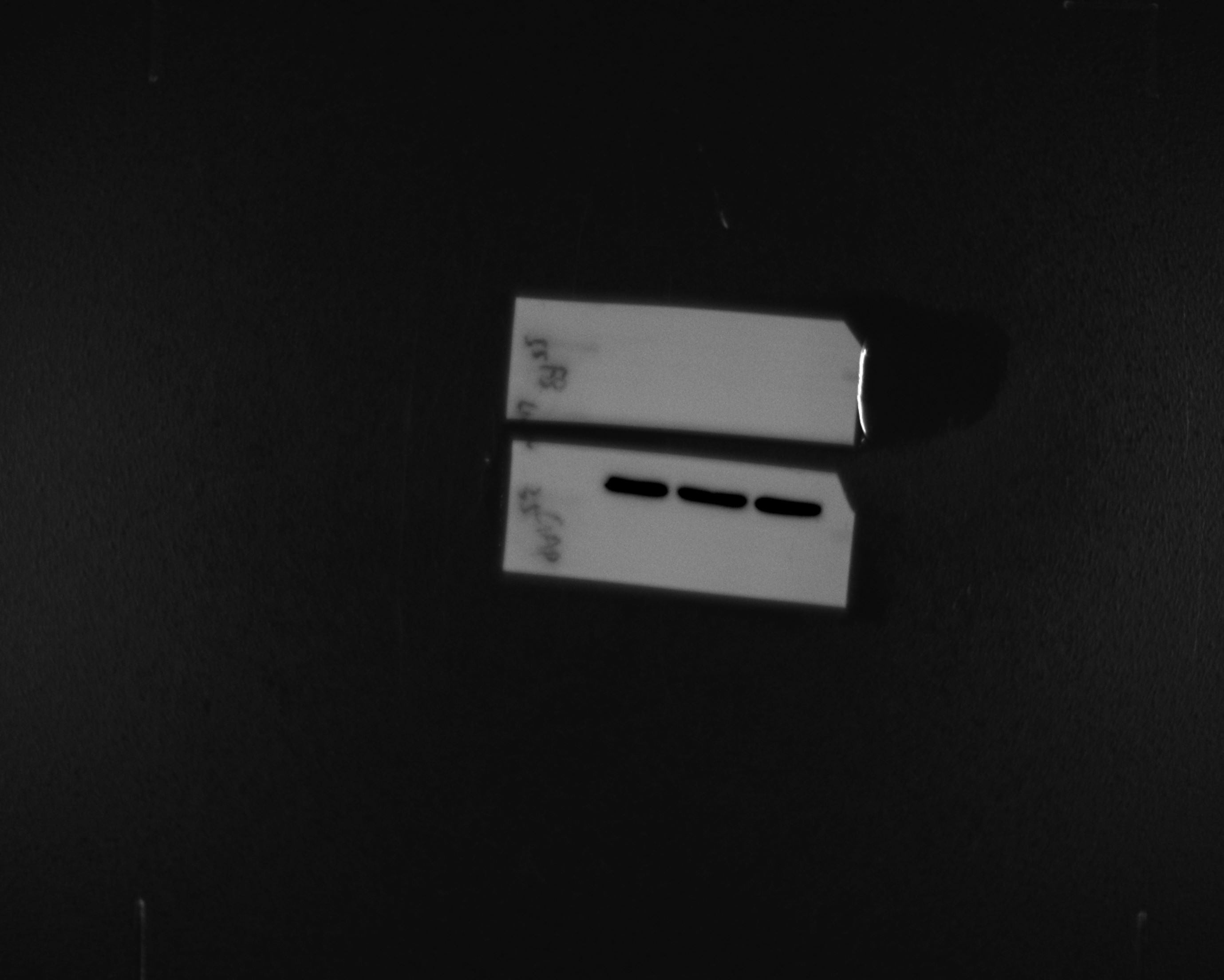

Supplement: Figure 3—source data 2. [file elife-101888-fig3-data2.zip › Figure 3D/T47D/GAPDH.jpg]

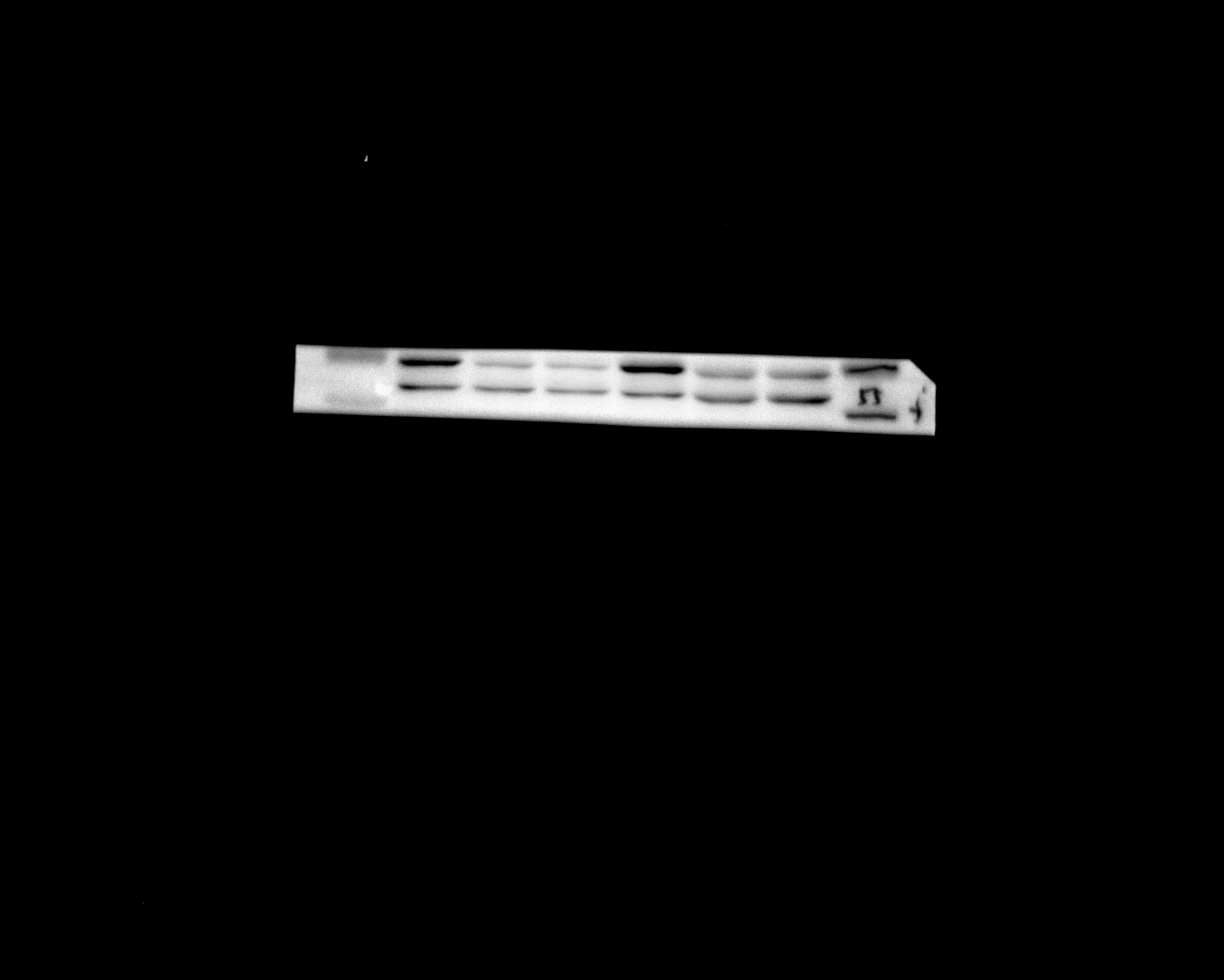

Supplement: Figure 3—source data 2. [file elife-101888-fig3-data2.zip › Figure 3F/MCF7/ERα.jpg]

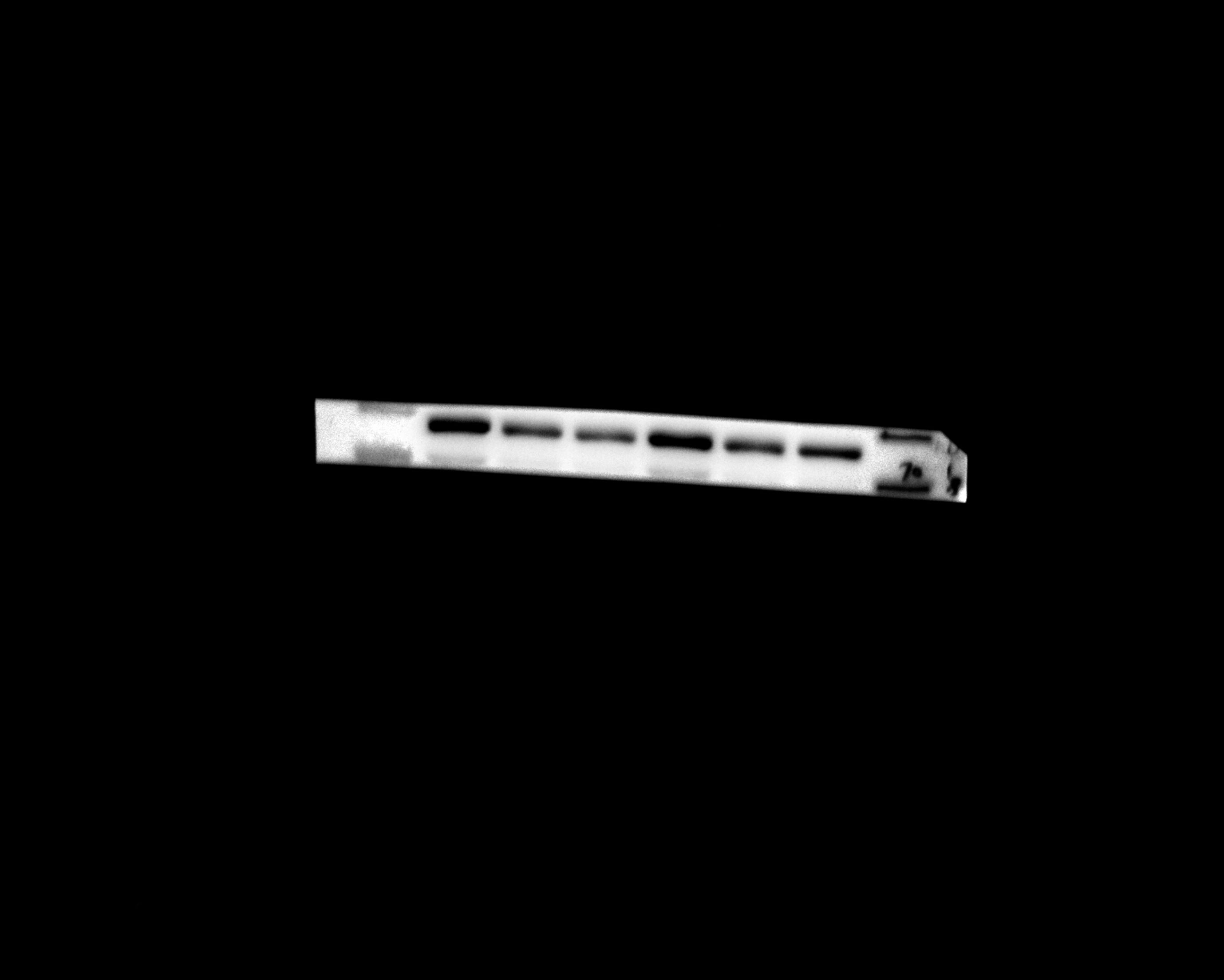

Supplement: Figure 3—source data 2. [file elife-101888-fig3-data2.zip › Figure 3F/MCF7/FOXO3A.jpg]

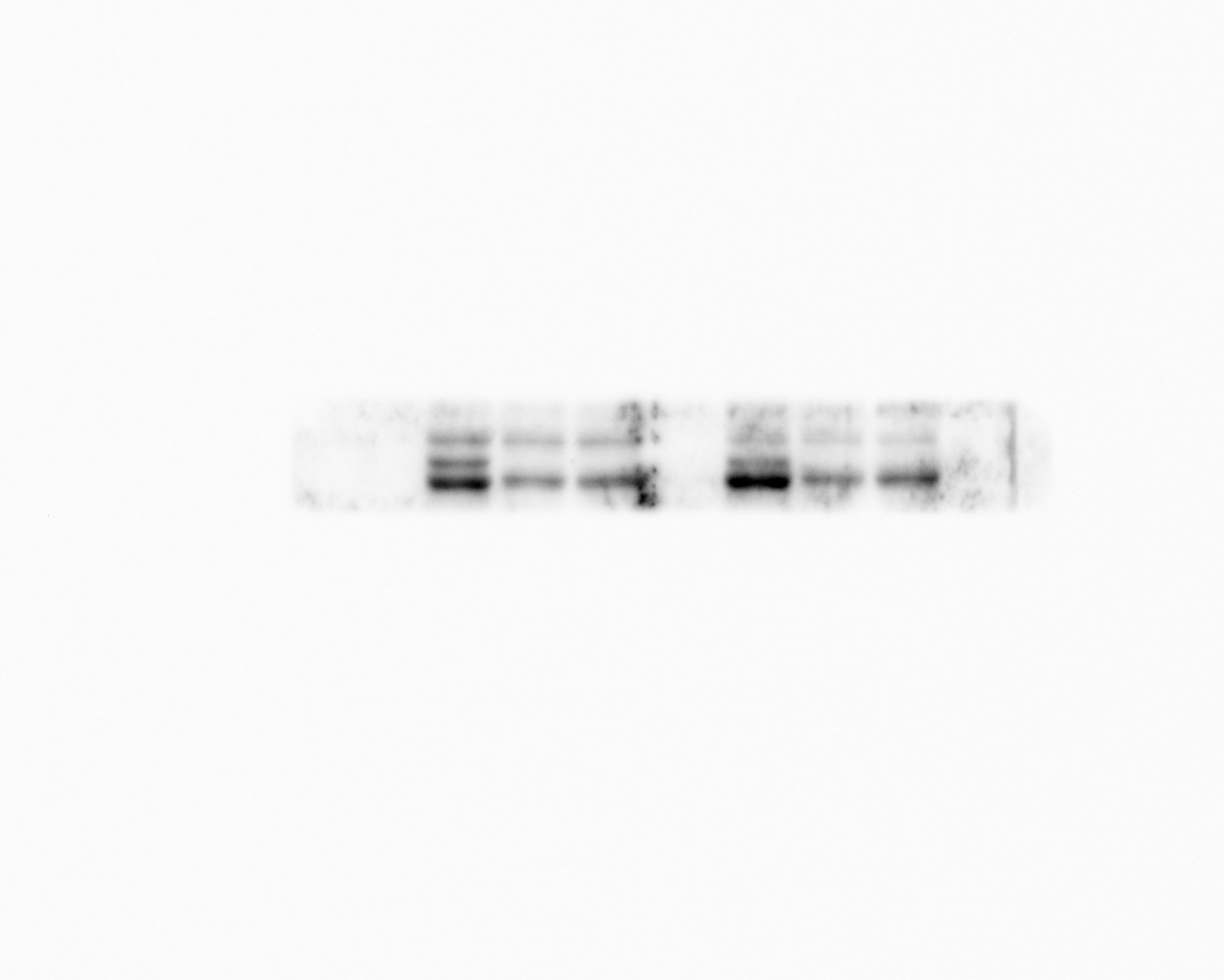

Supplement: Figure 3—source data 2. [file elife-101888-fig3-data2.zip › Figure 3F/MCF7/FRMD8.jpg]

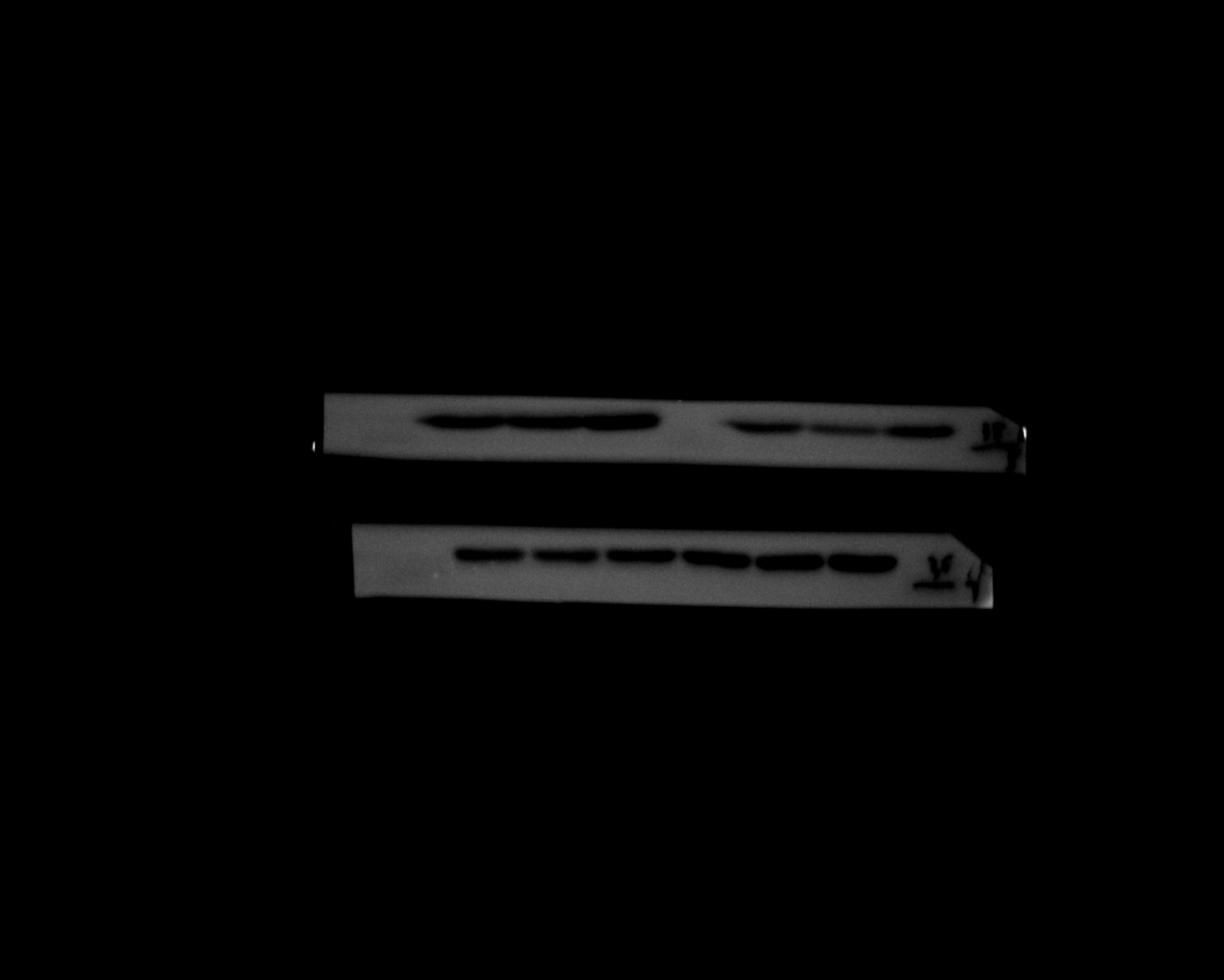

Supplement: Figure 3—source data 2. [file elife-101888-fig3-data2.zip › Figure 3F/MCF7/GAPDH.jpg]

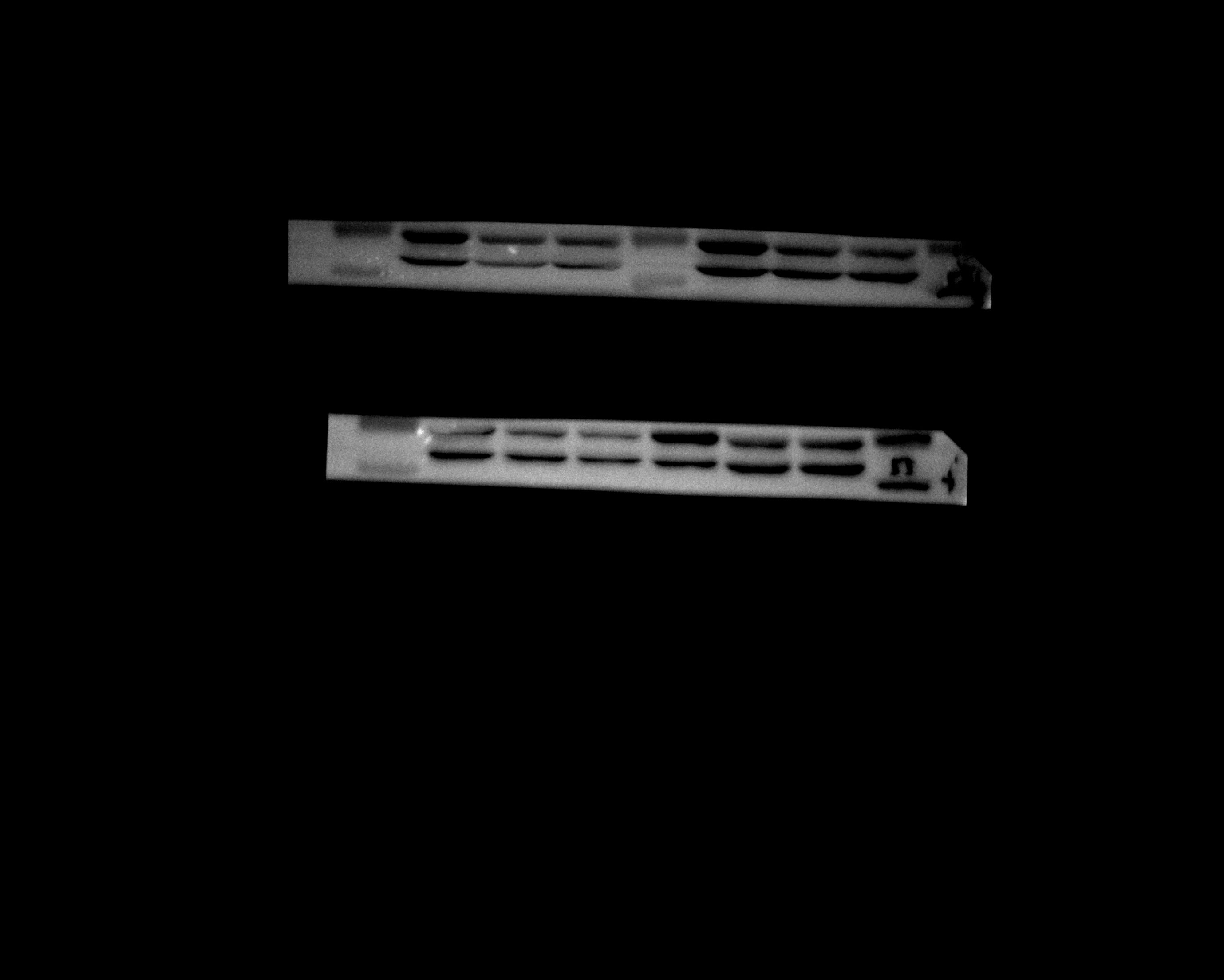

Supplement: Figure 3—source data 2. [file elife-101888-fig3-data2.zip › Figure 3F/T47D/ERα.jpg]

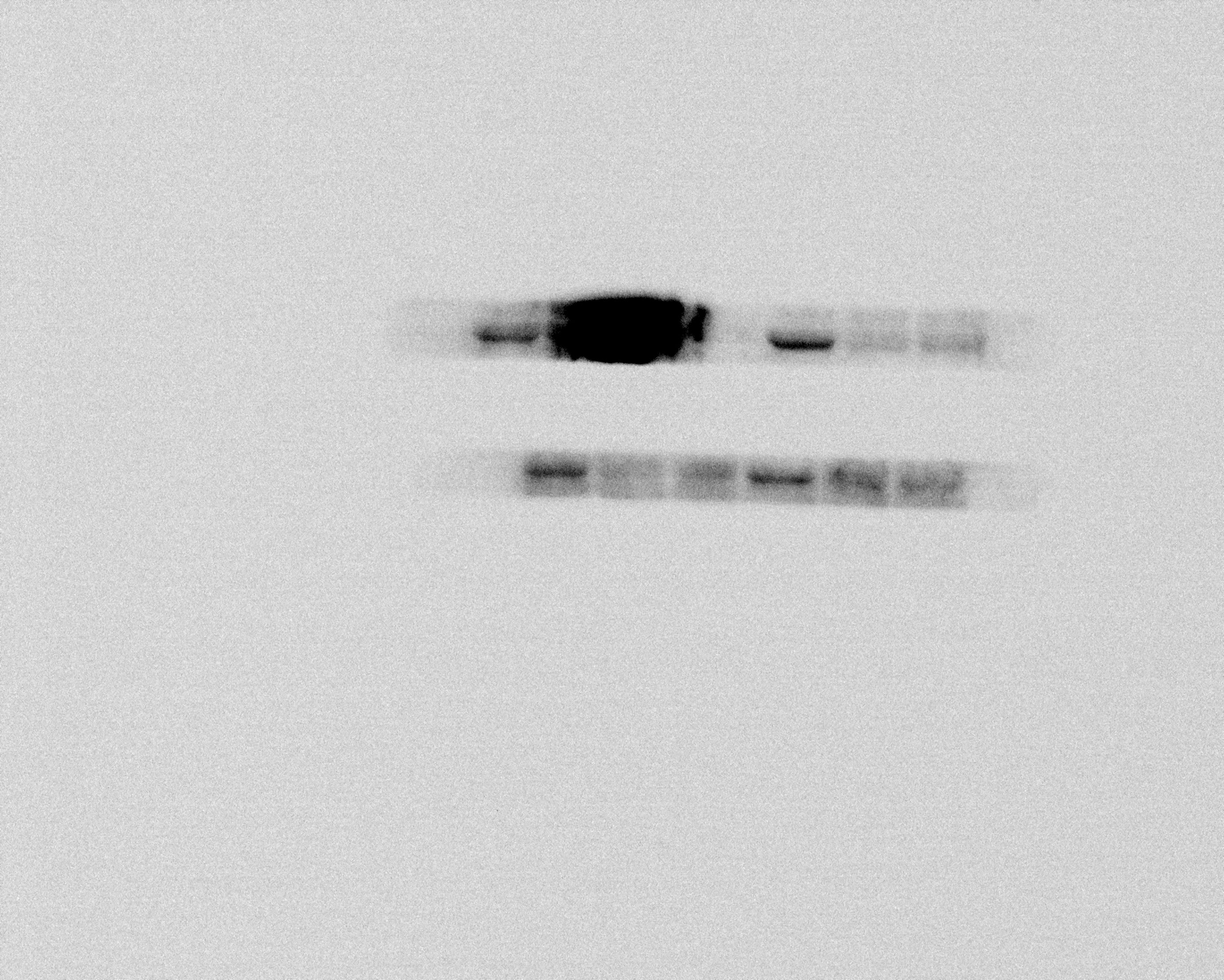

Supplement: Figure 3—source data 2. [file elife-101888-fig3-data2.zip › Figure 3F/T47D/FRMD8.jpg]

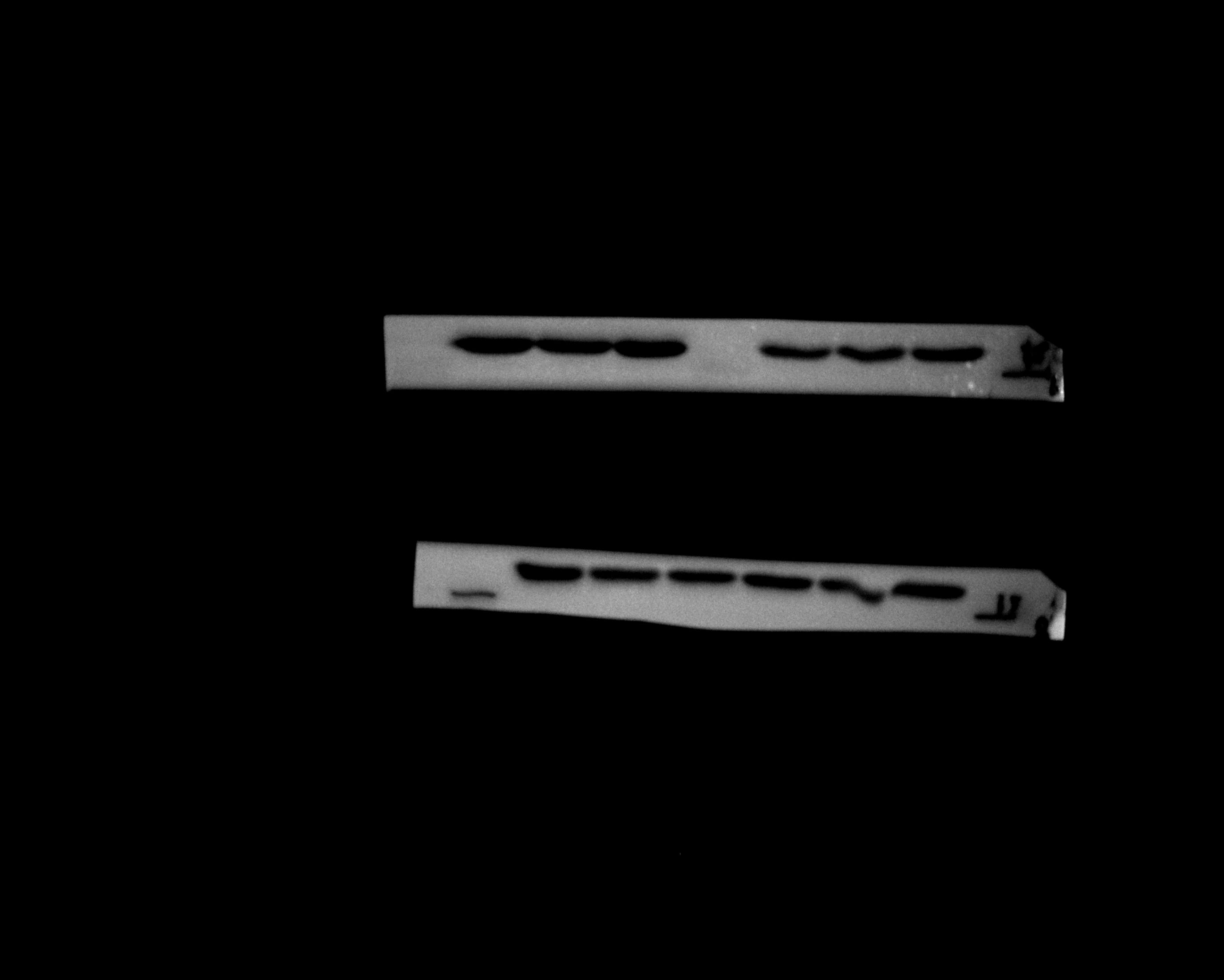

Supplement: Figure 3—source data 2. [file elife-101888-fig3-data2.zip › Figure 3F/T47D/GAPDH.jpg]

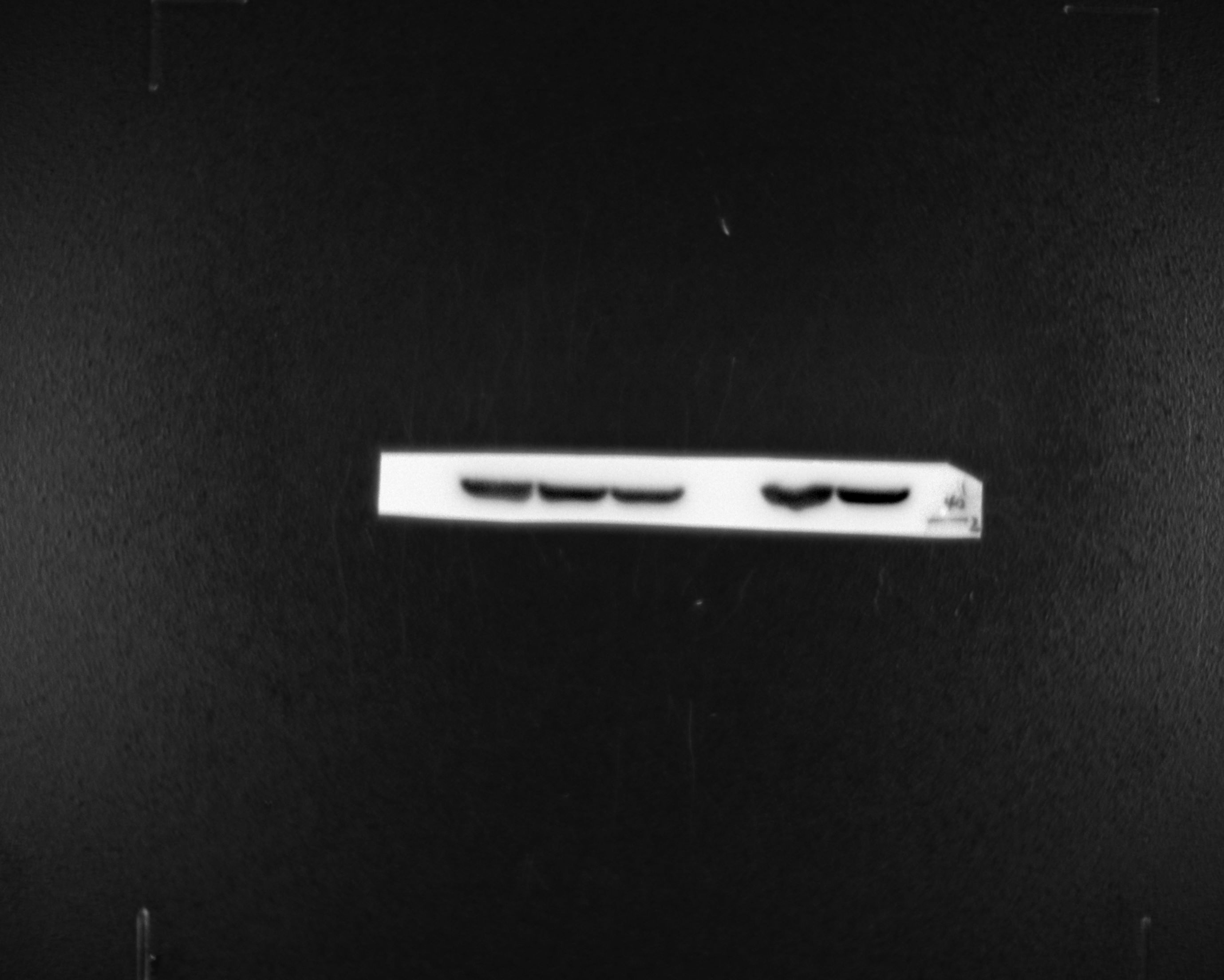

Supplement: Figure 3—source data 2. [file elife-101888-fig3-data2.zip › Figure 3G/MCF7/Actin.jpg]

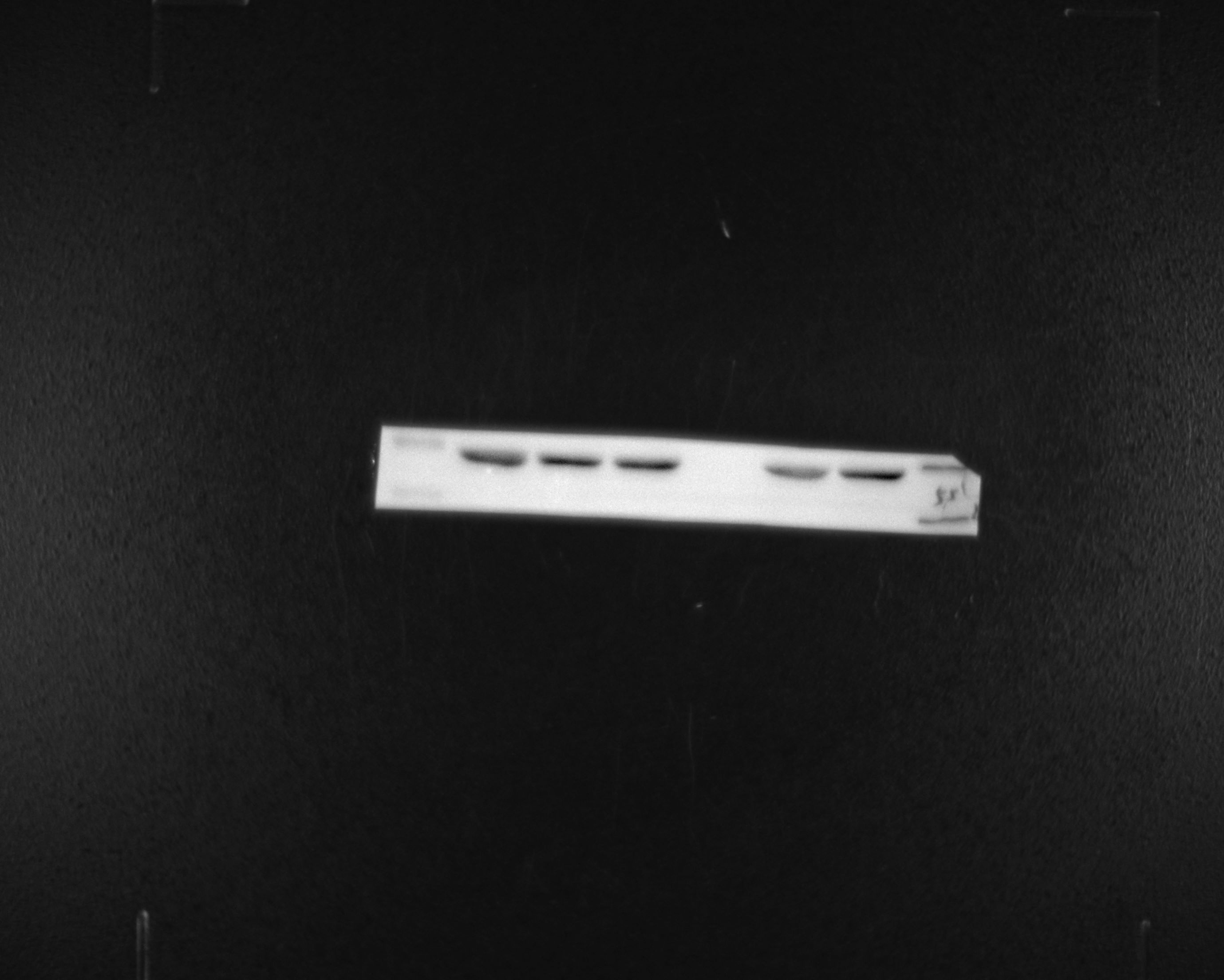

Supplement: Figure 3—source data 2. [file elife-101888-fig3-data2.zip › Figure 3G/MCF7/ERα.jpg]

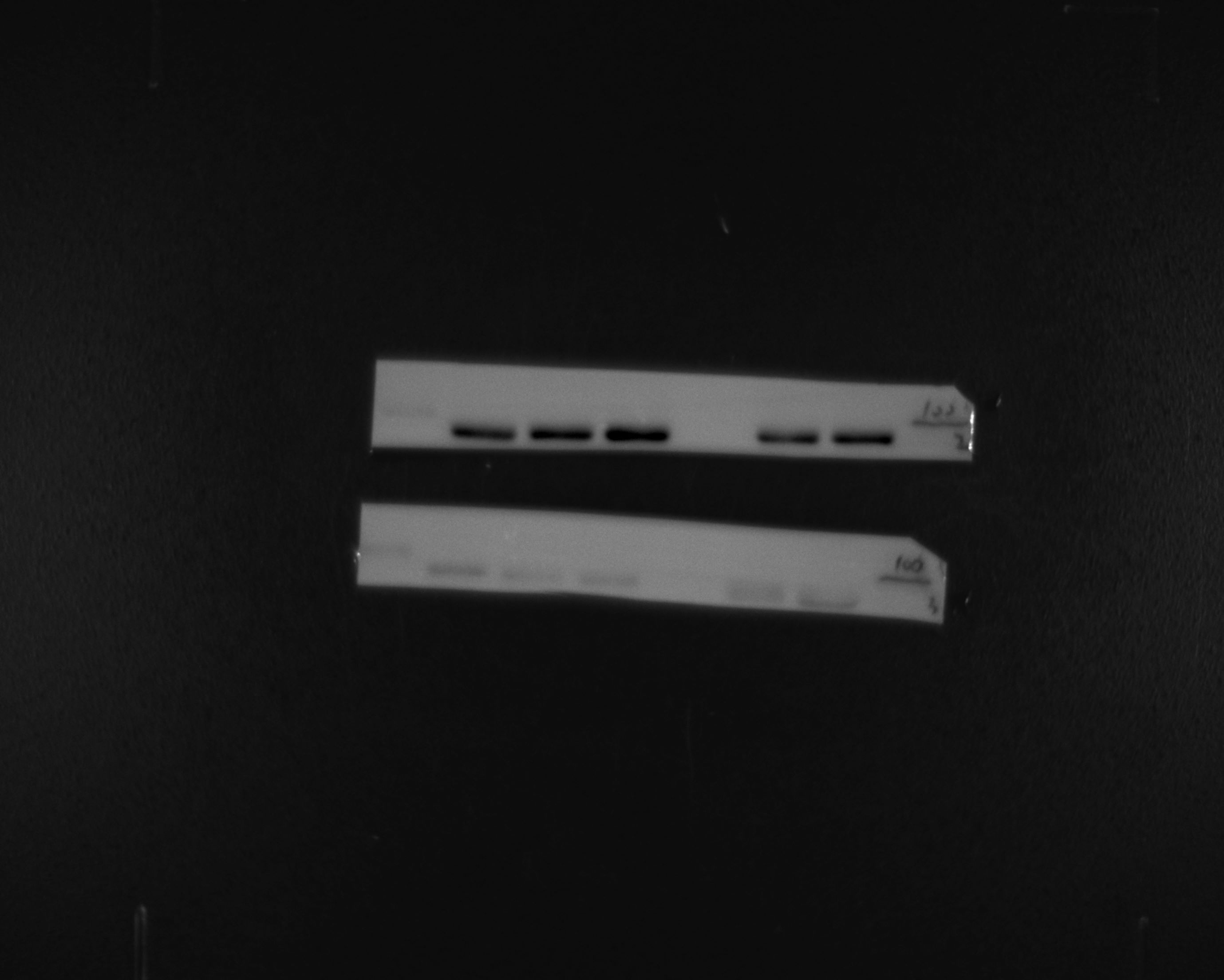

Supplement: Figure 3—source data 2. [file elife-101888-fig3-data2.zip › Figure 3G/MCF7/FOXO3A.jpg]

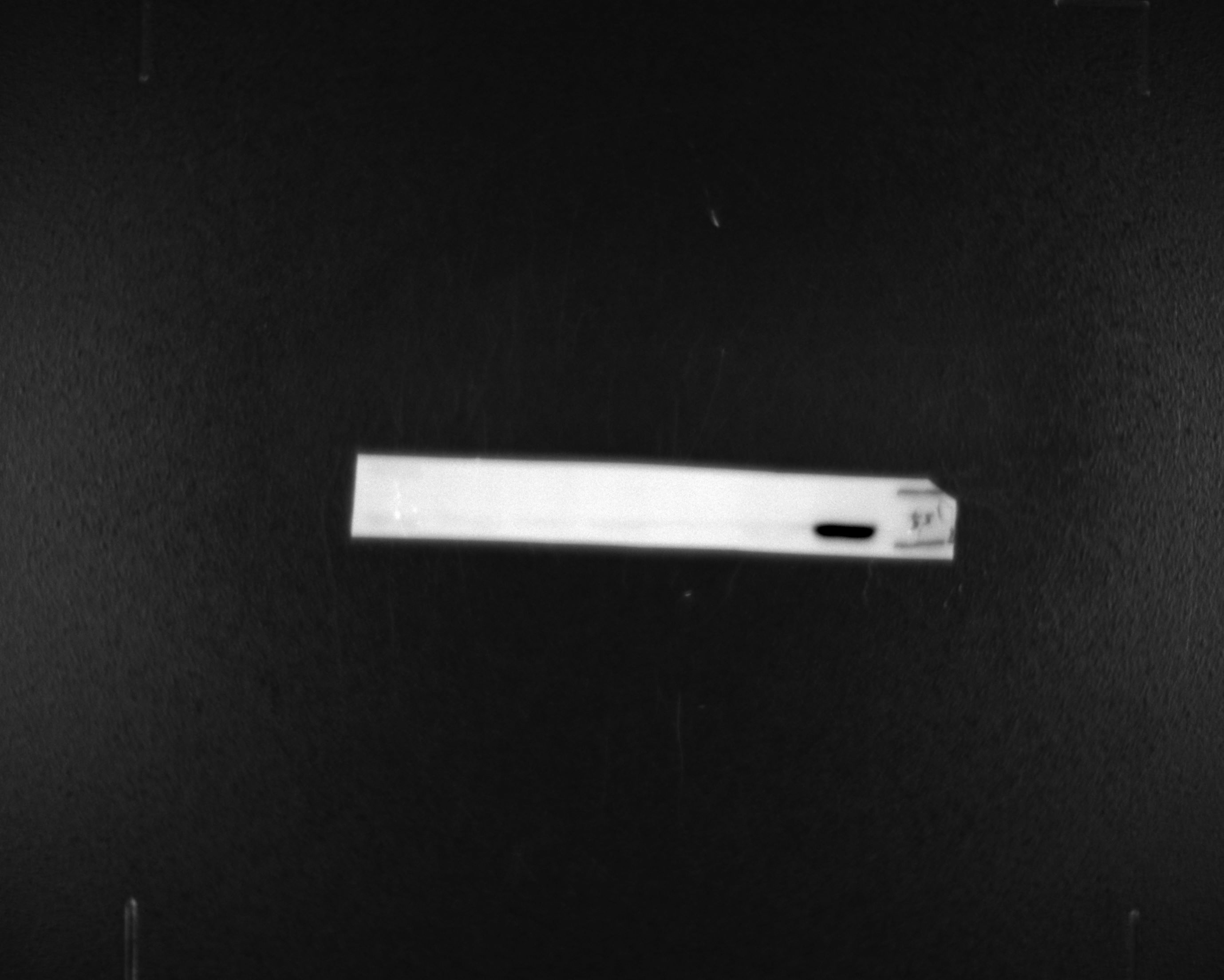

Supplement: Figure 3—source data 2. [file elife-101888-fig3-data2.zip › Figure 3G/MCF7/FRMD8.jpg]

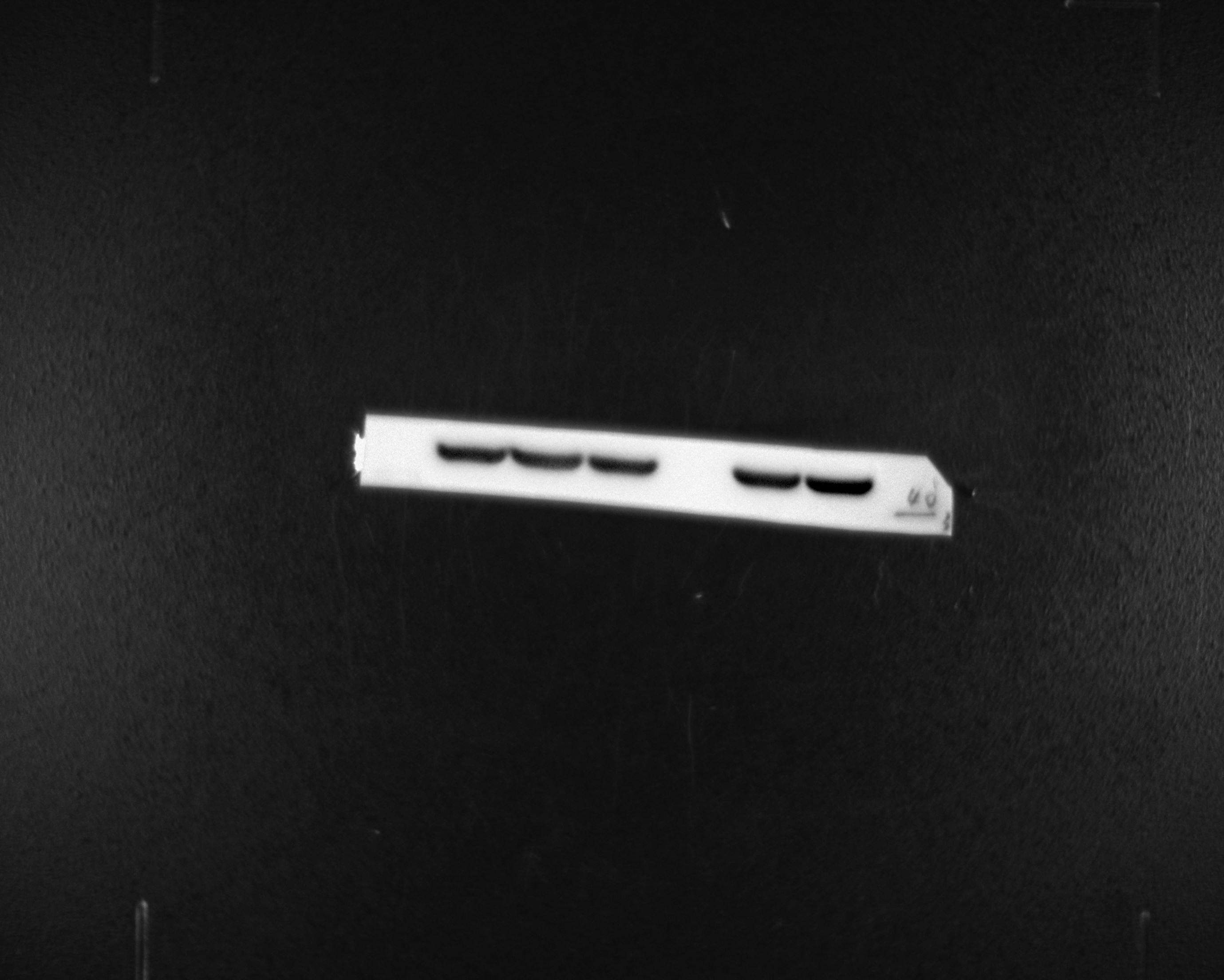

Supplement: Figure 3—source data 2. [file elife-101888-fig3-data2.zip › Figure 3G/T47D/Actin.jpg]

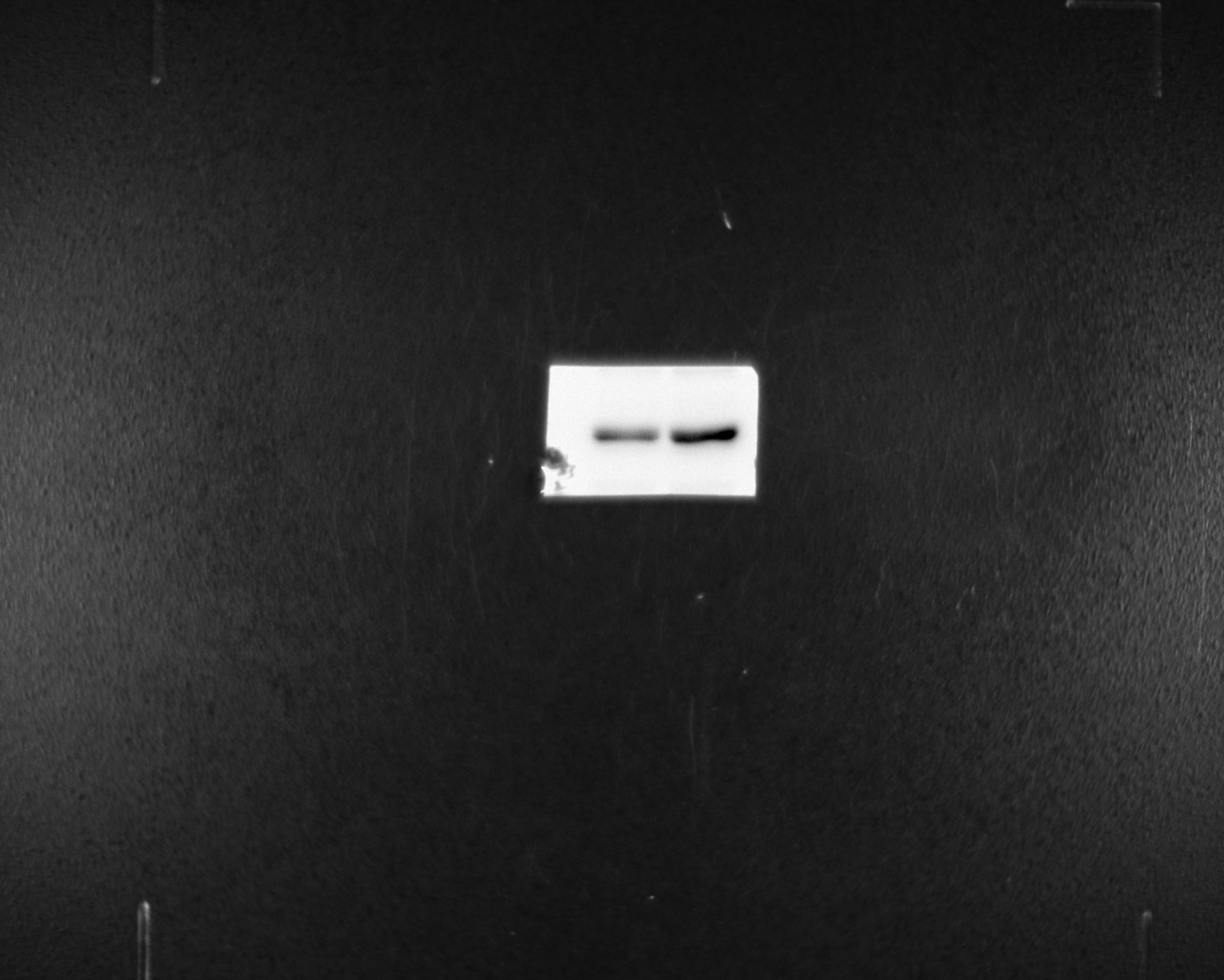

Supplement: Figure 3—source data 2. [file elife-101888-fig3-data2.zip › Figure 3G/T47D/ERα.jpg]

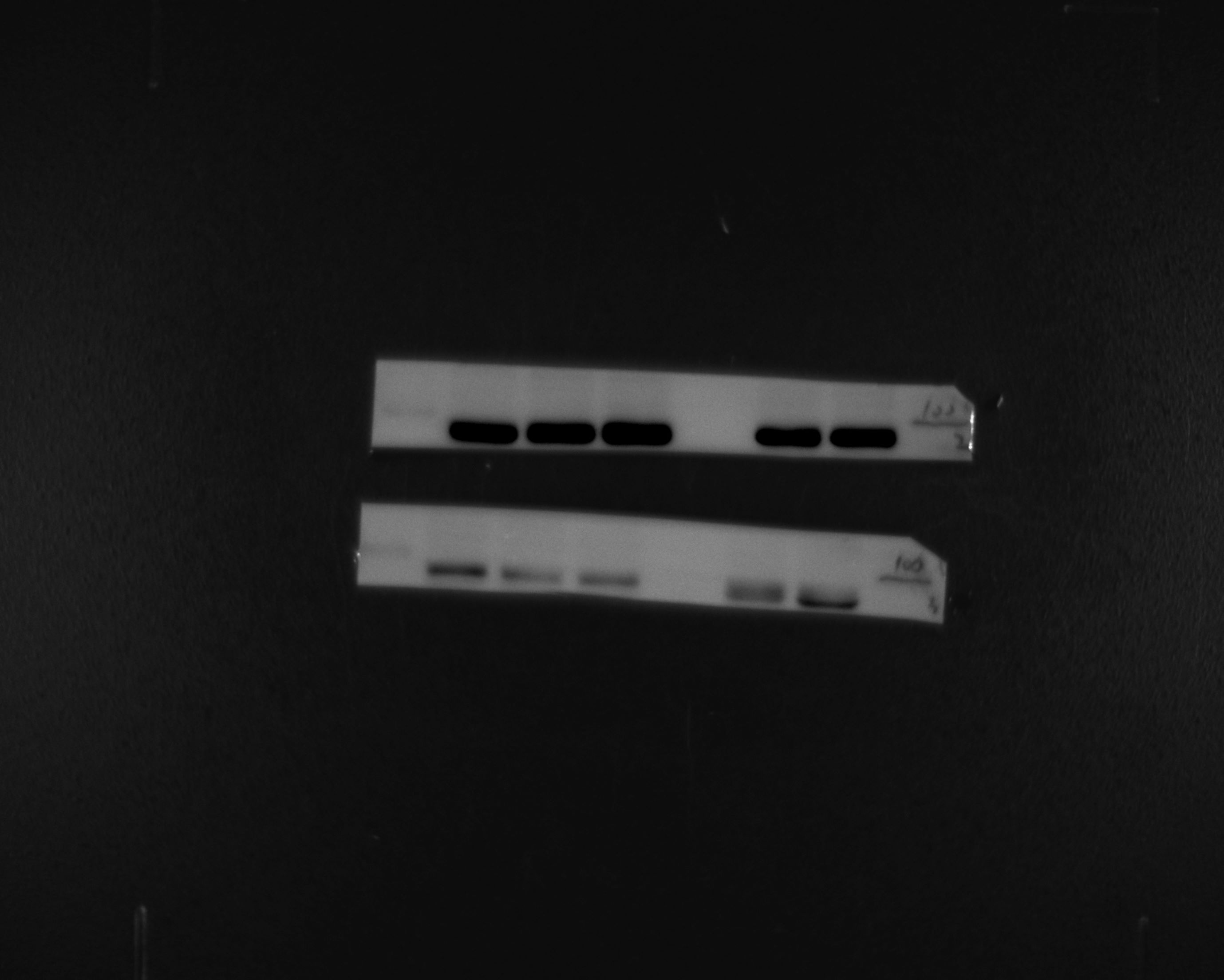

Supplement: Figure 3—source data 2. [file elife-101888-fig3-data2.zip › Figure 3G/T47D/FOXO3A.jpg]

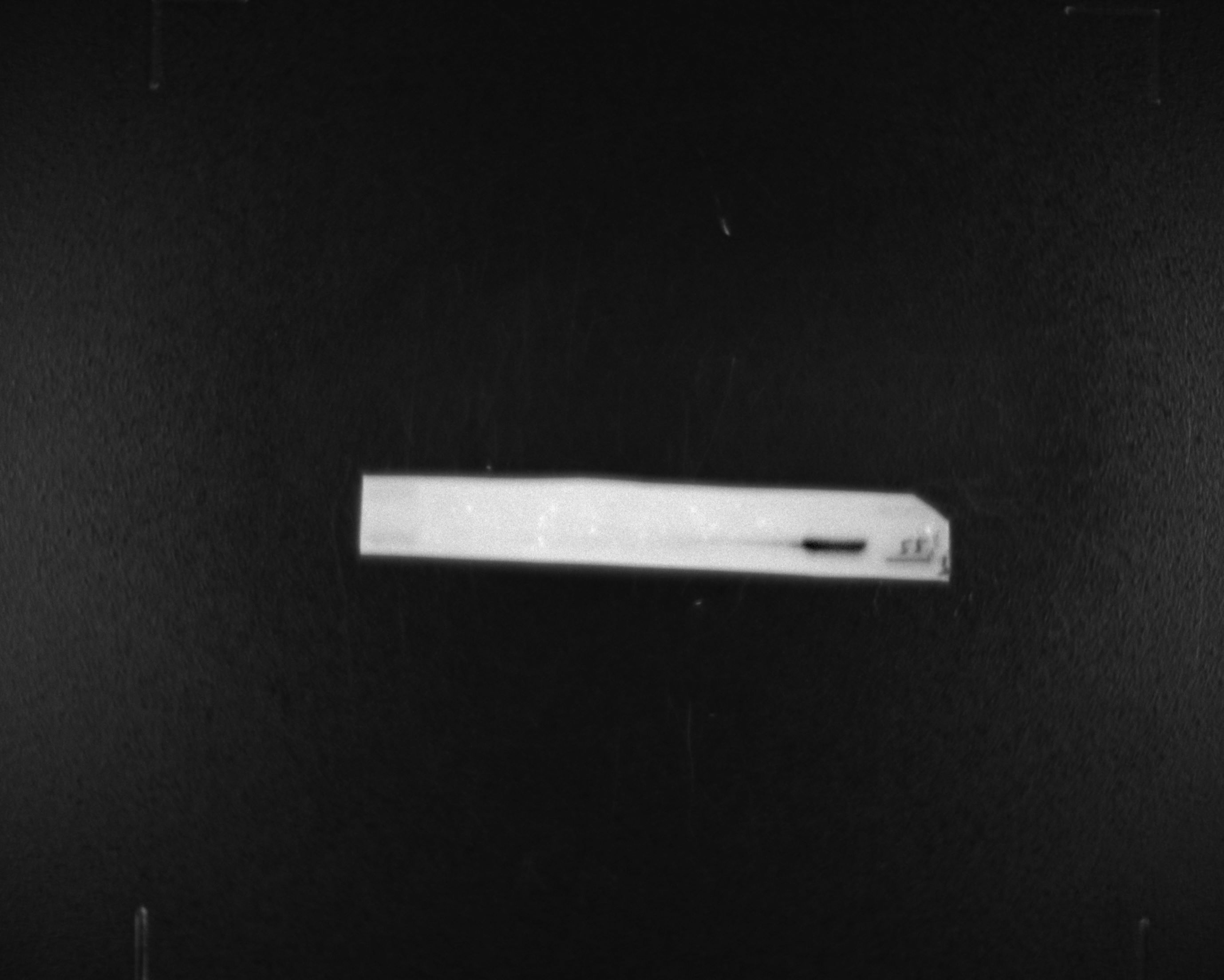

Supplement: Figure 3—source data 2. [file elife-101888-fig3-data2.zip › Figure 3G/T47D/FRMD8.jpg]

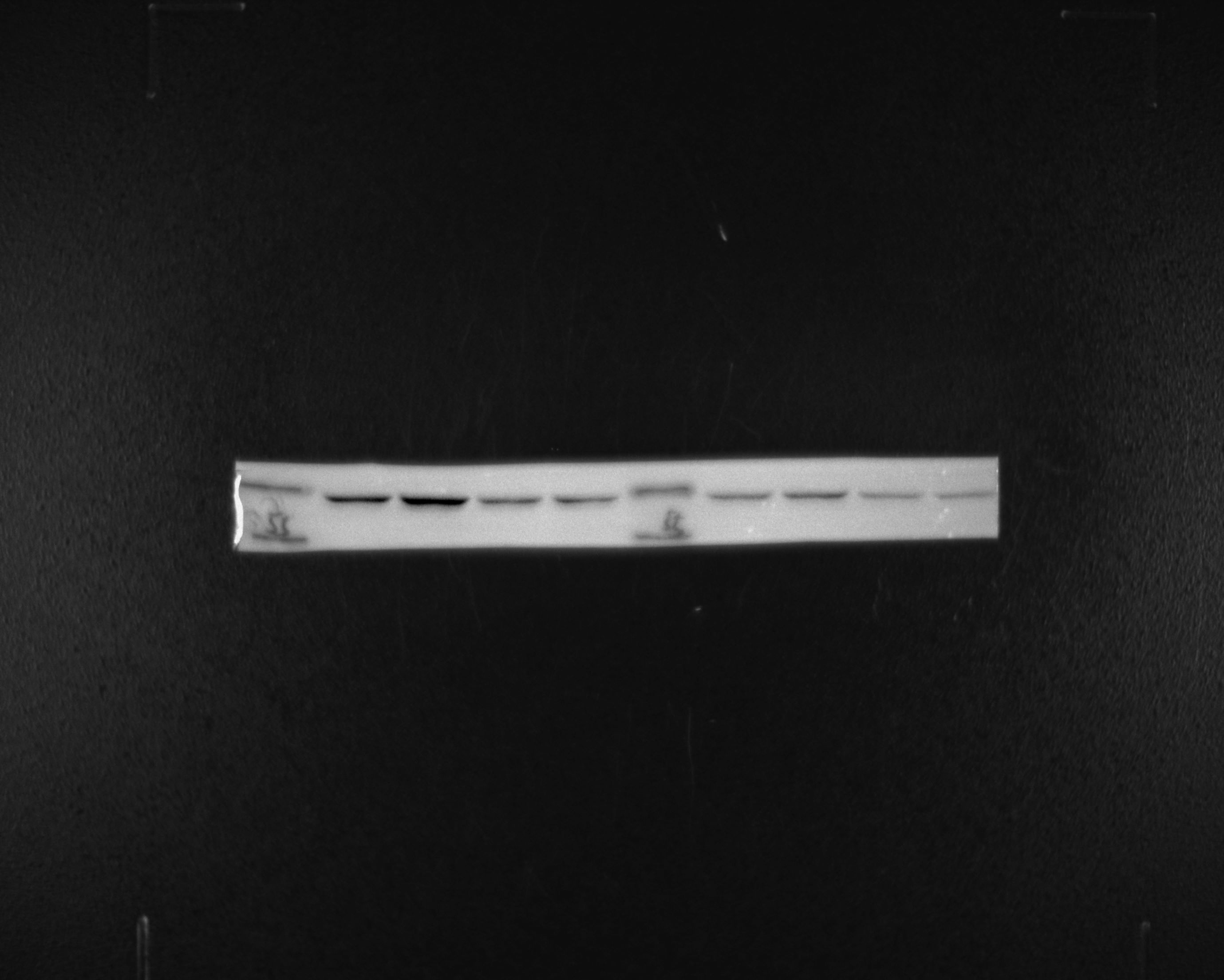

Supplement: Figure 3—source data 2. [file elife-101888-fig3-data2.zip › Figure 3H/ERα.jpg]

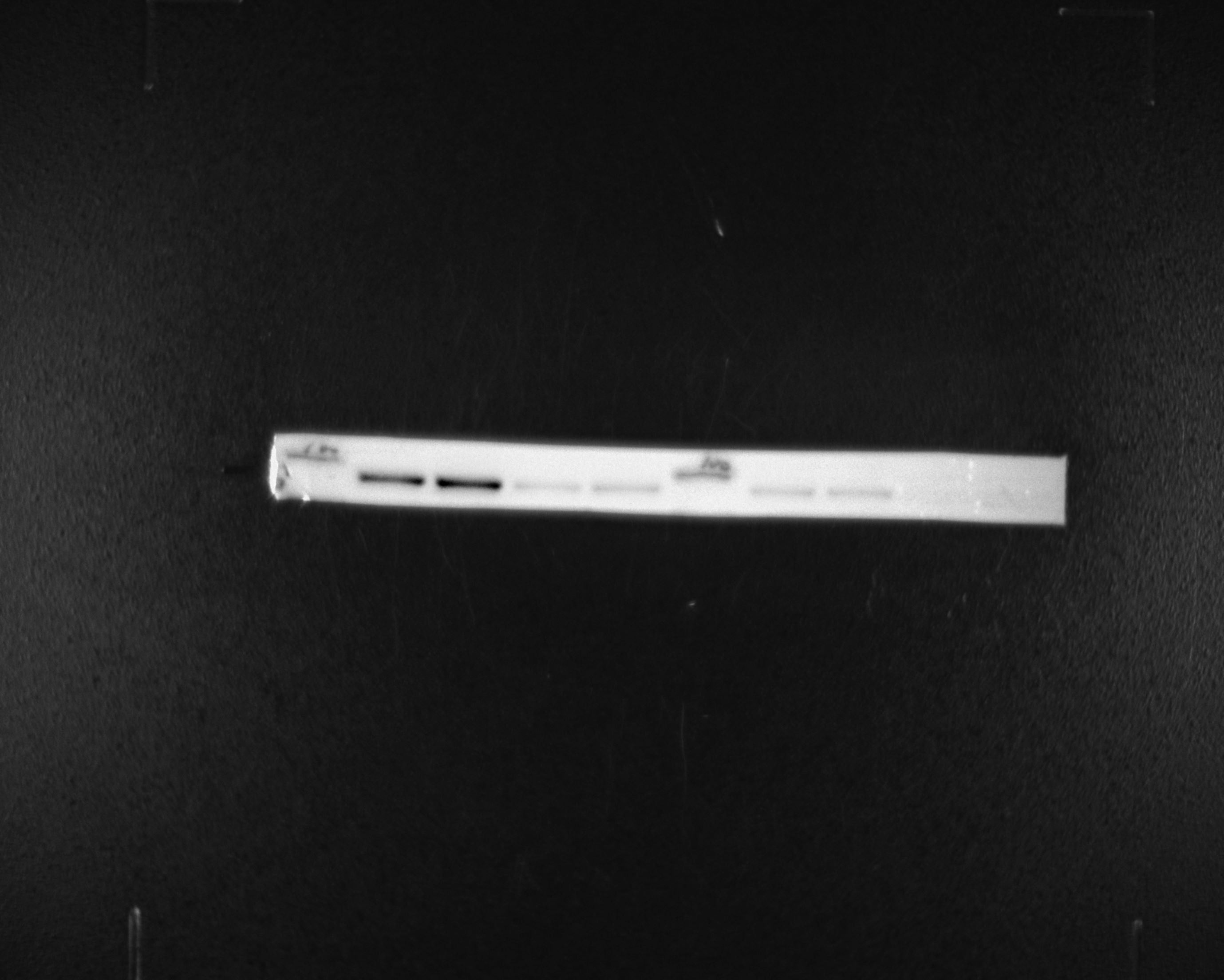

Supplement: Figure 3—source data 2. [file elife-101888-fig3-data2.zip › Figure 3H/FOXO3A.jpg]

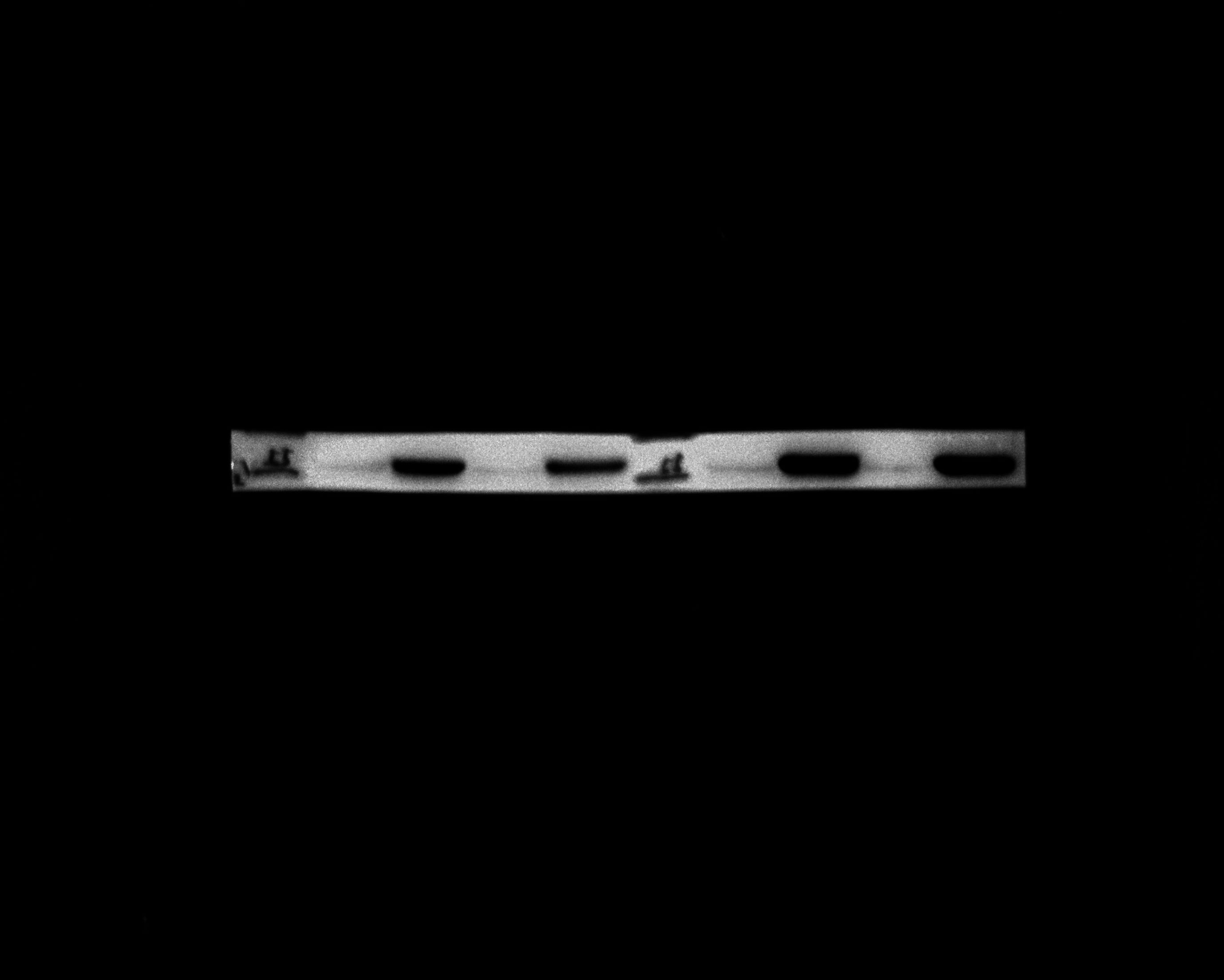

Supplement: Figure 3—source data 2. [file elife-101888-fig3-data2.zip › Figure 3H/FRMD8.jpg]

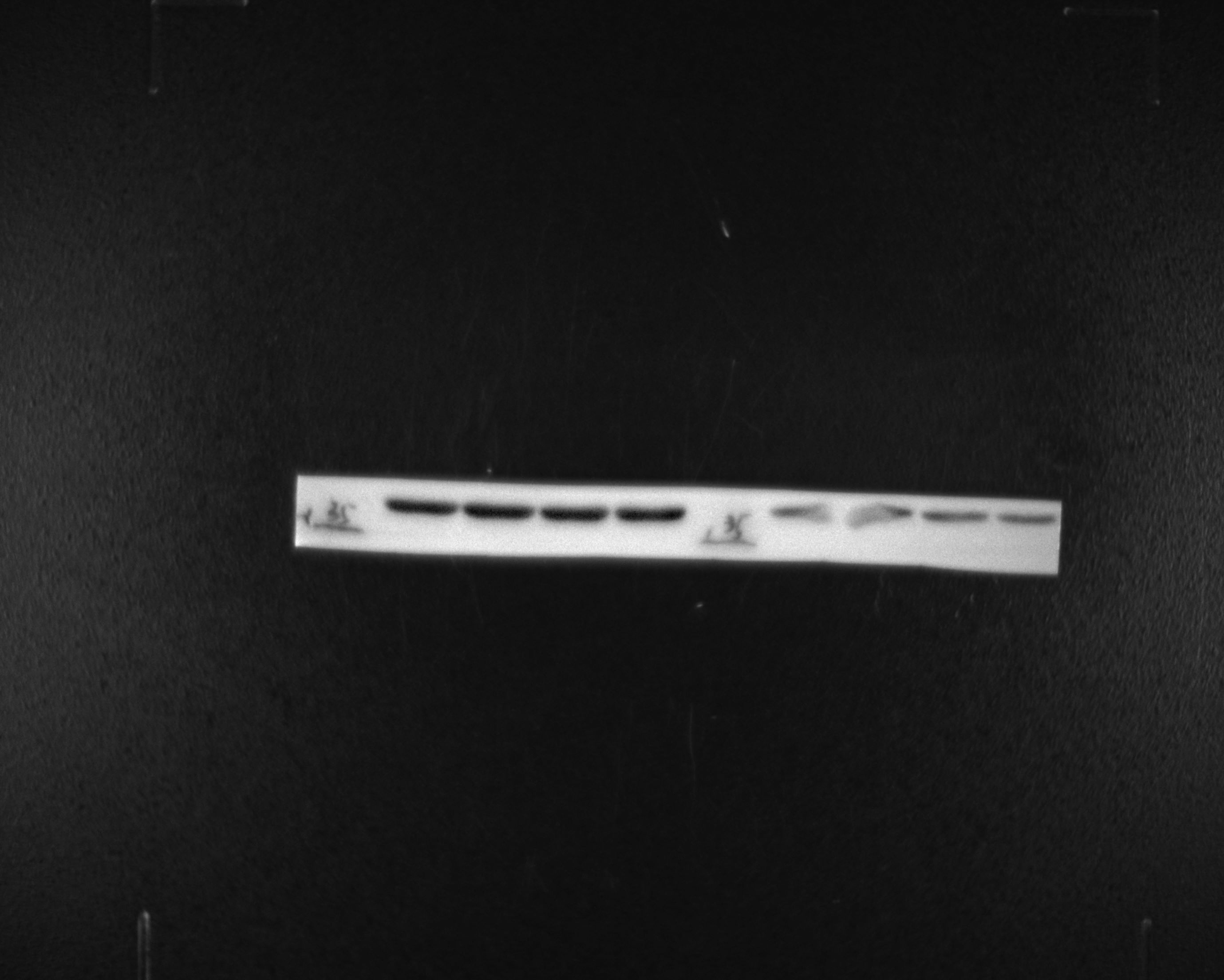

Supplement: Figure 3—source data 2. [file elife-101888-fig3-data2.zip › Figure 3H/GAPDH.jpg]

**Figure 4-source data:** Unedited western blot pictures for figure 4.

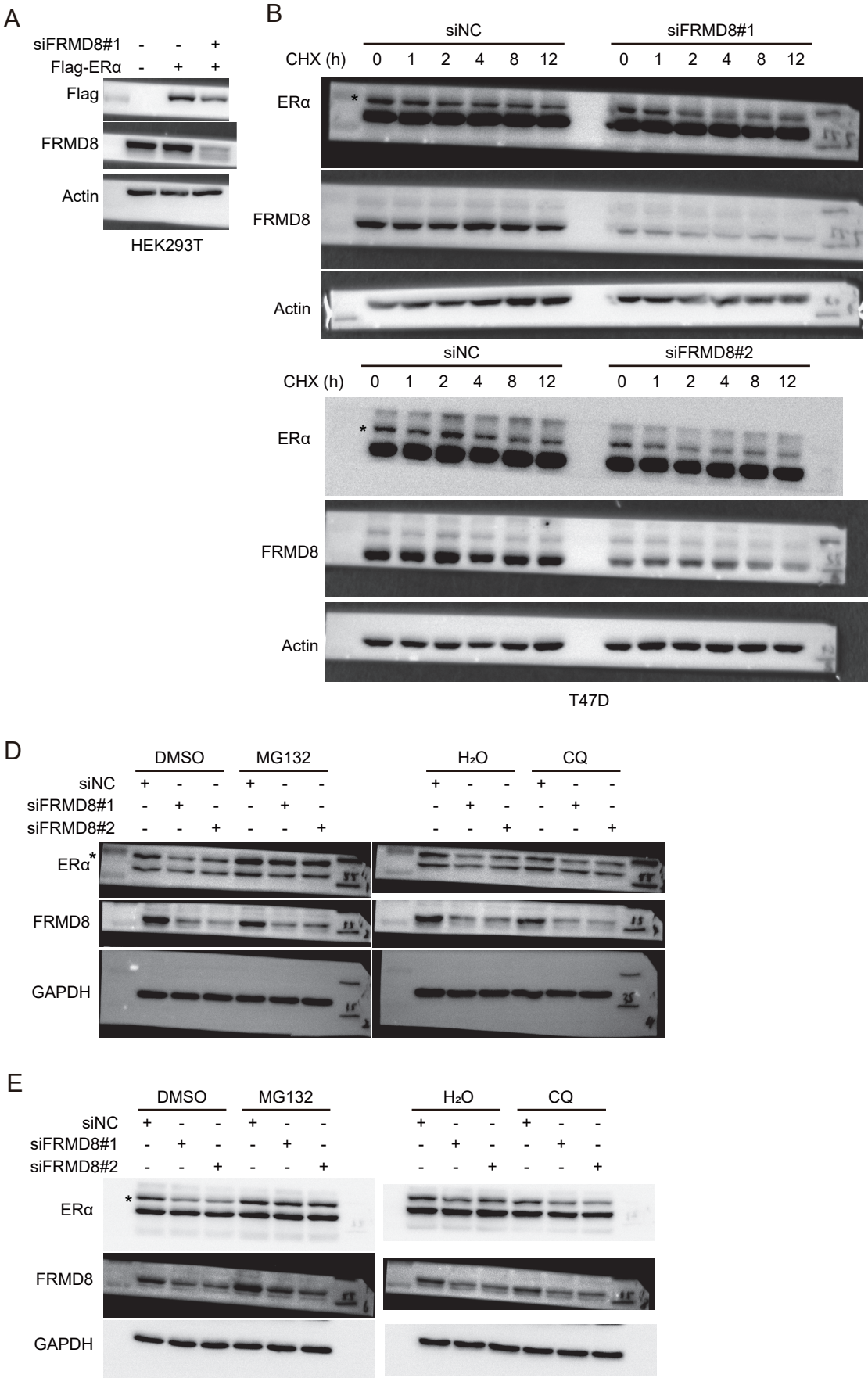

Supplement: Figure 4—source data 1. [file elife-101888-fig4-data1.pdf]

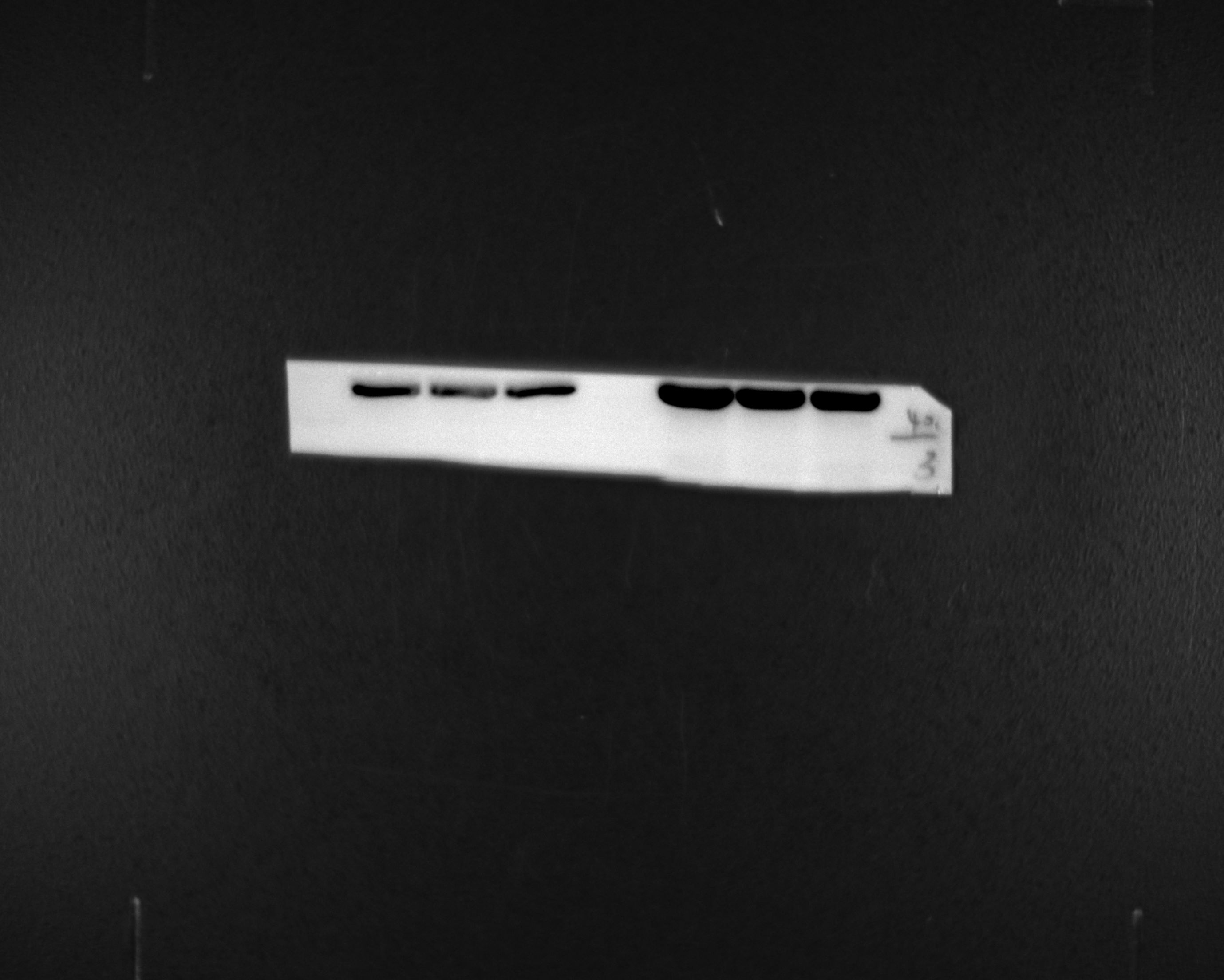

Supplement: Figure 4—source data 2. [file elife-101888-fig4-data2.zip › Figure 4A/Actin.jpg]

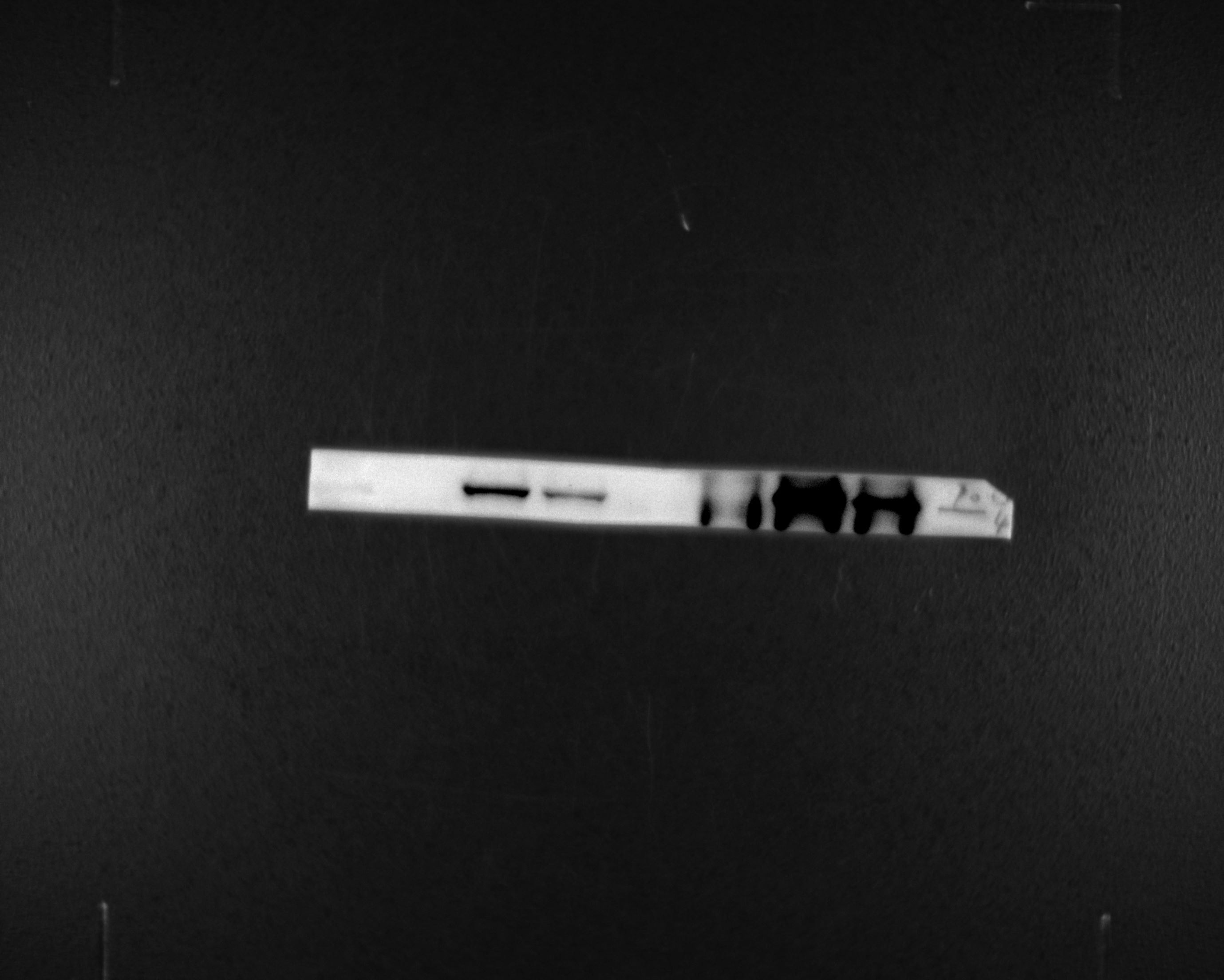

Supplement: Figure 4—source data 2. [file elife-101888-fig4-data2.zip › Figure 4A/Flag.jpg]

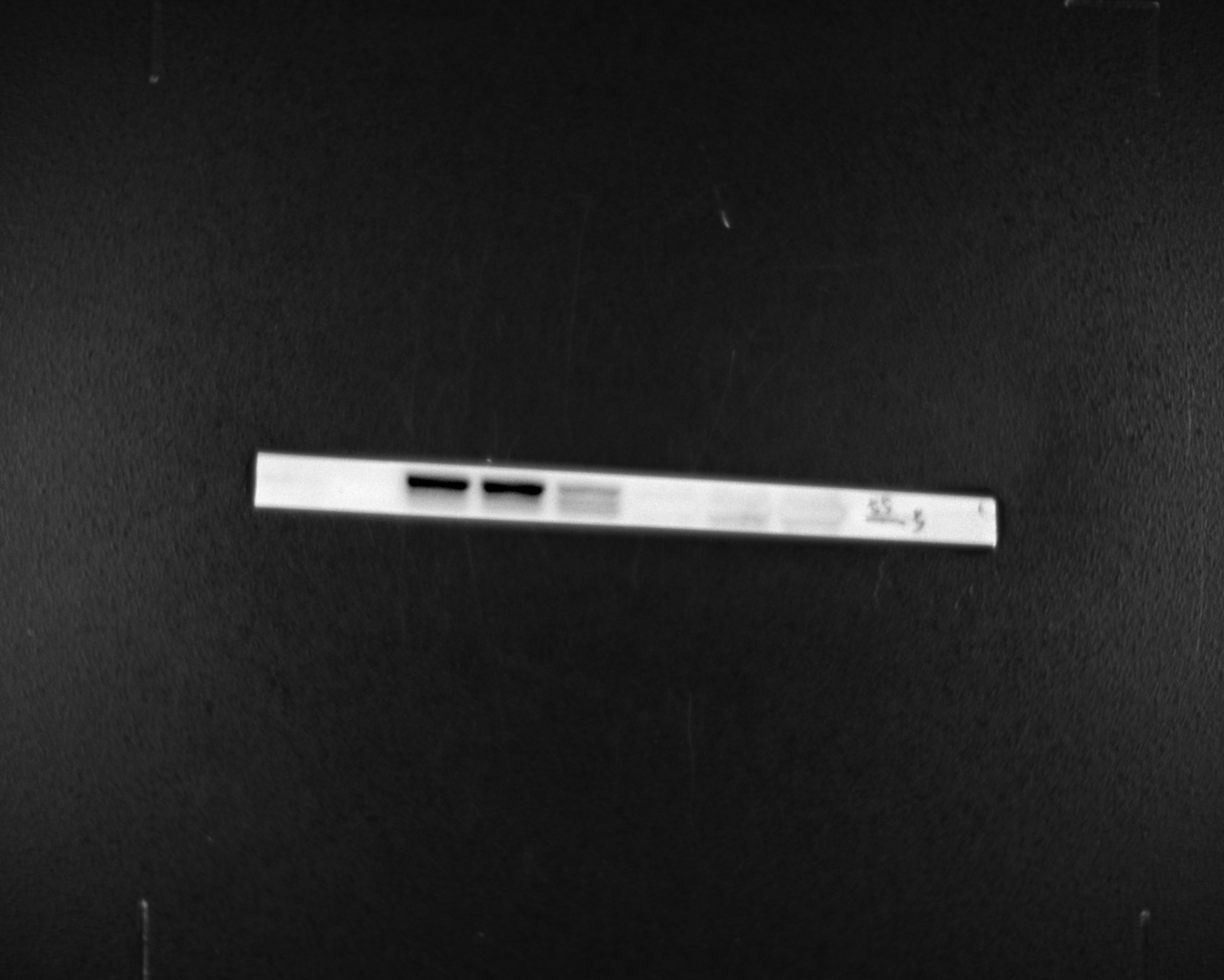

Supplement: Figure 4—source data 2. [file elife-101888-fig4-data2.zip › Figure 4A/FRMD8.jpg]

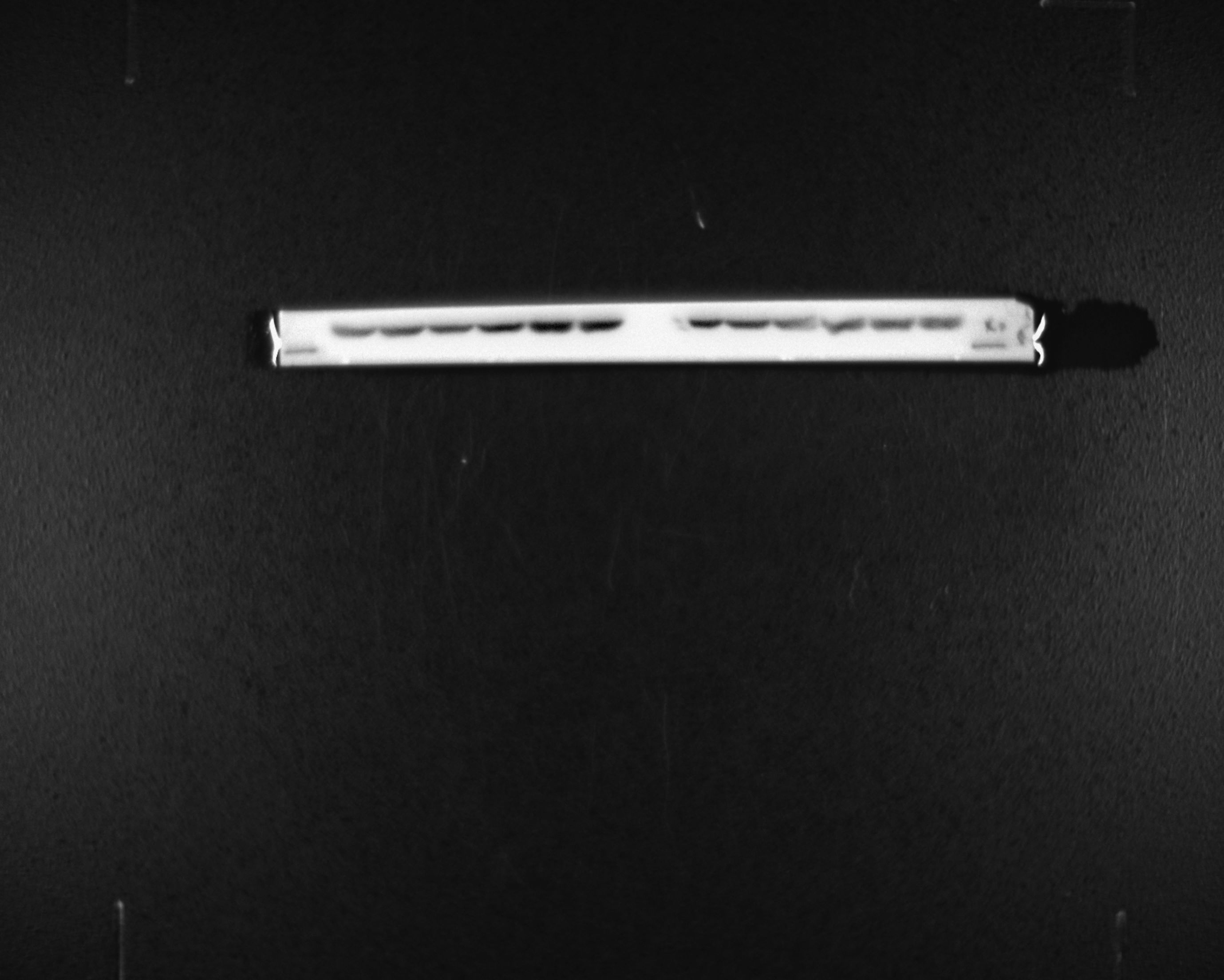

Supplement: Figure 4—source data 2. [file elife-101888-fig4-data2.zip › Figure 4B/Actin 1.jpg]

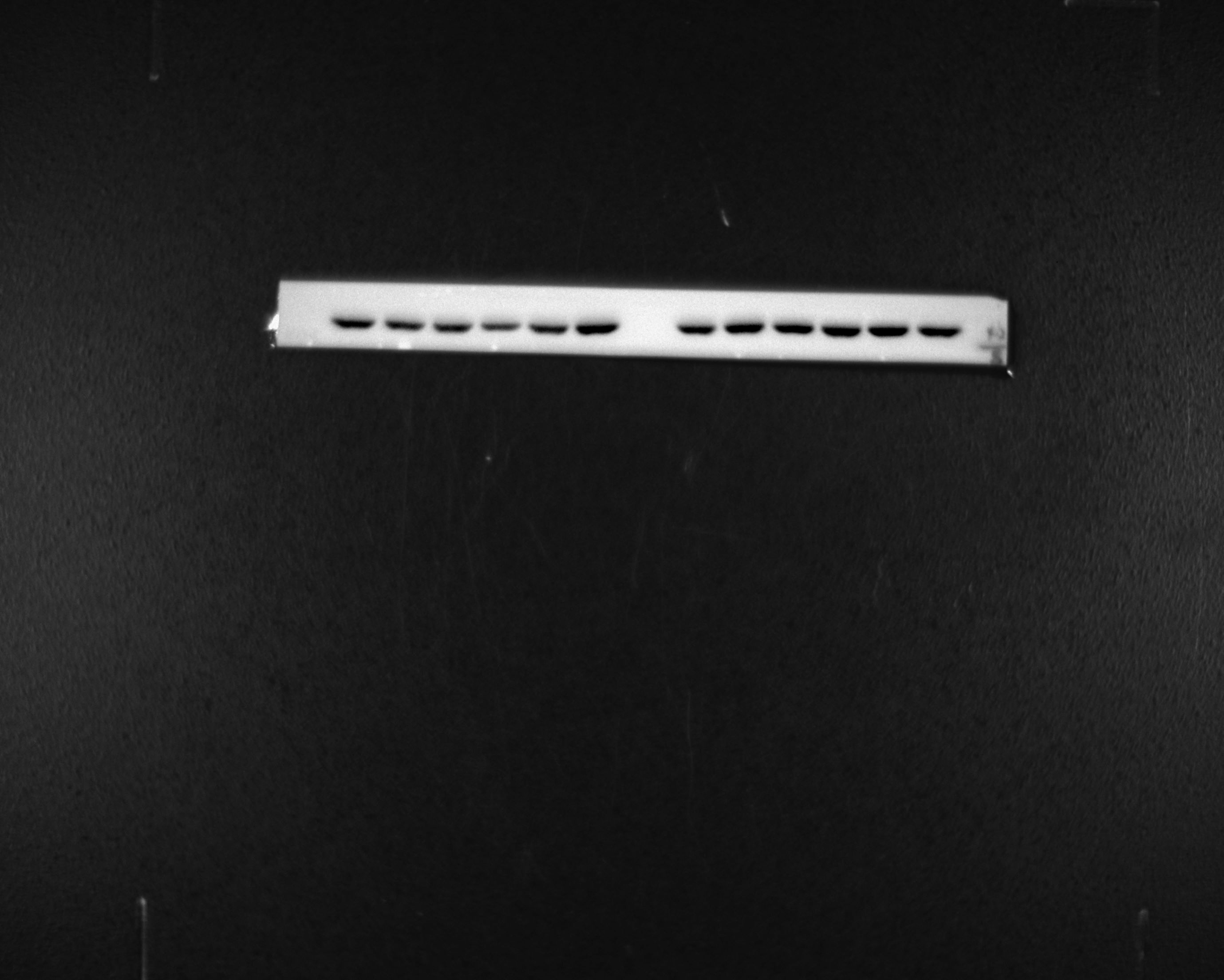

Supplement: Figure 4—source data 2. [file elife-101888-fig4-data2.zip › Figure 4B/Actin 2.jpg]

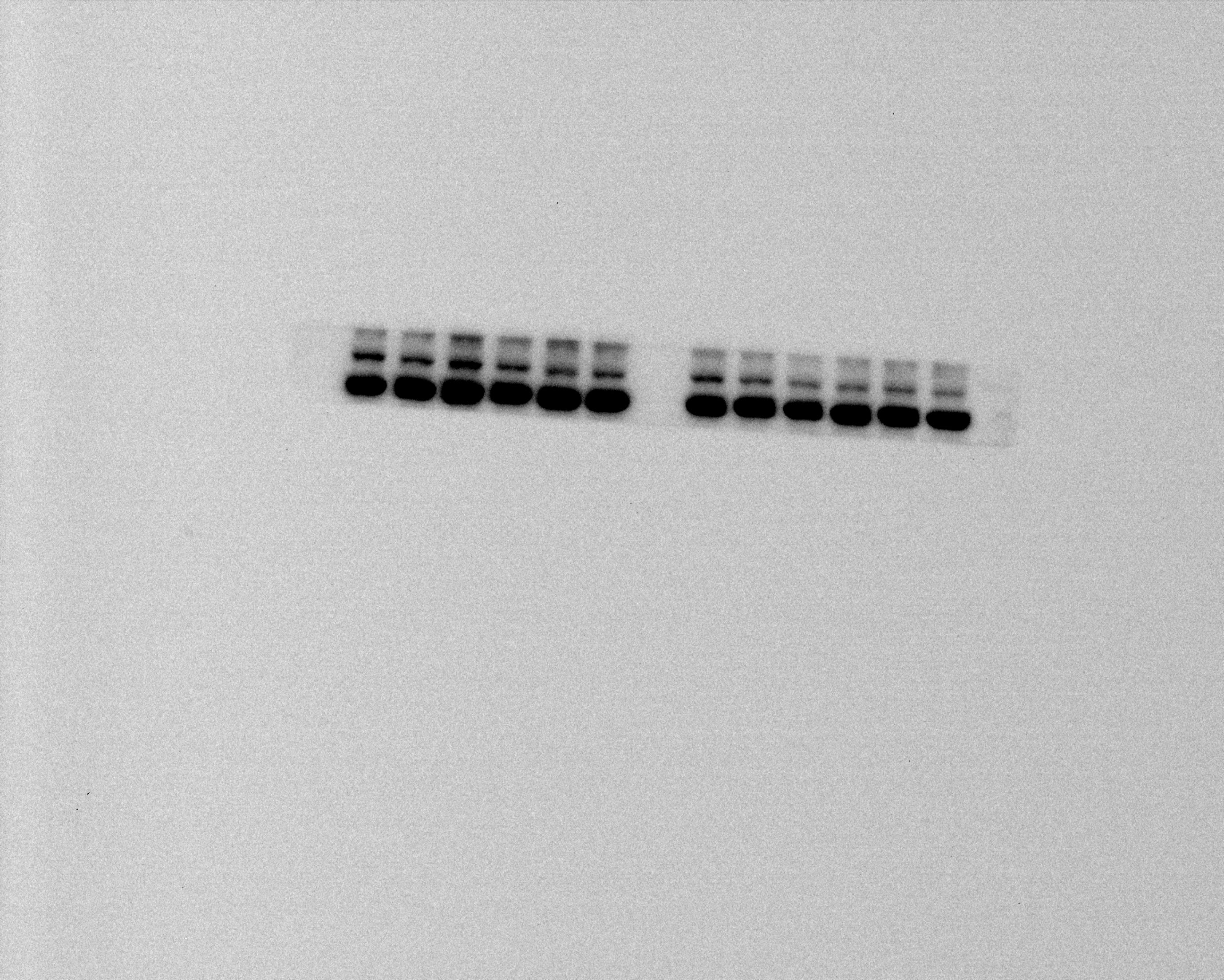

Supplement: Figure 4—source data 2. [file elife-101888-fig4-data2.zip › Figure 4B/ERα 2.jpg]

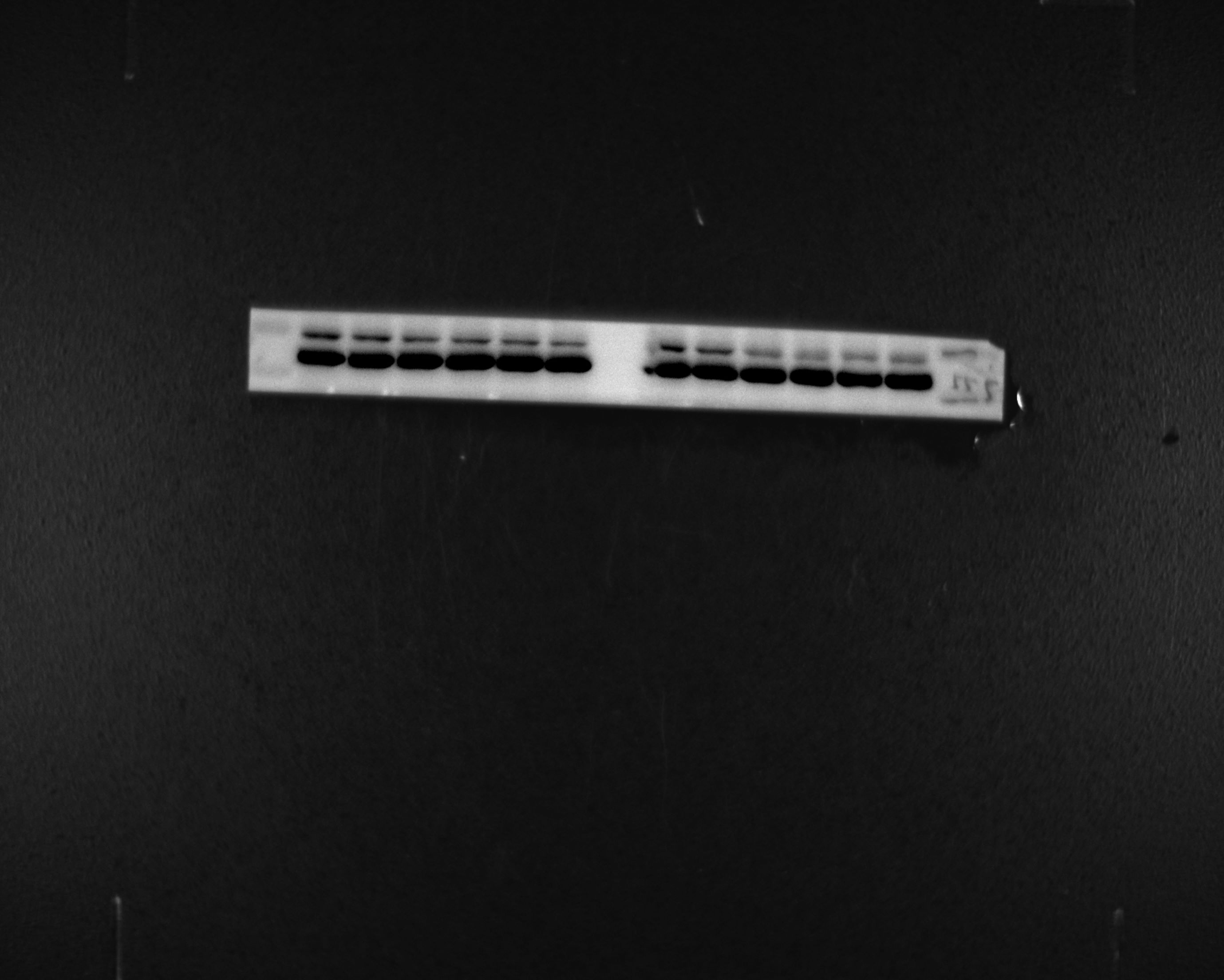

Supplement: Figure 4—source data 2. [file elife-101888-fig4-data2.zip › Figure 4B/ERα 1.jpg]

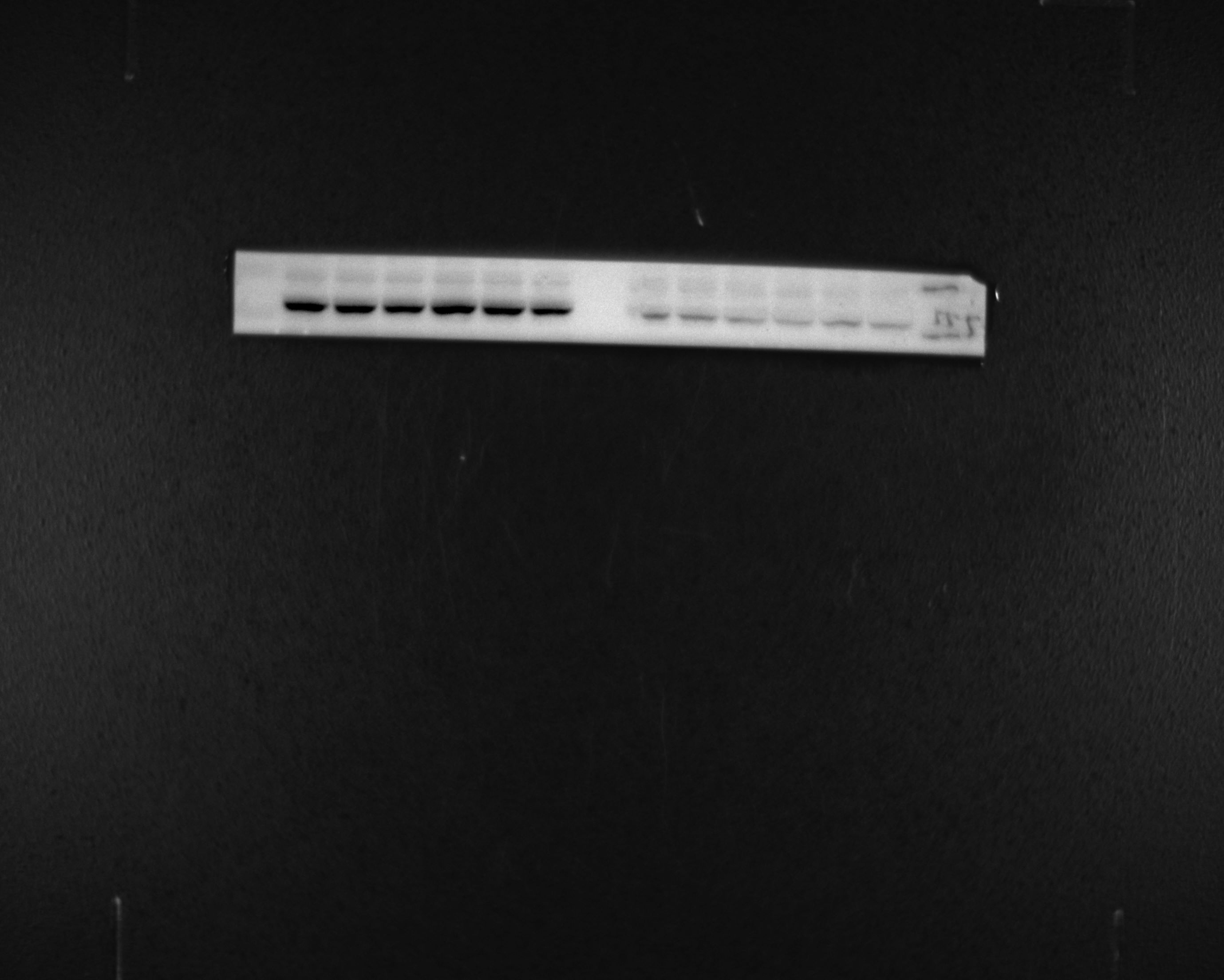

Supplement: Figure 4—source data 2. [file elife-101888-fig4-data2.zip › Figure 4B/FRMD8 1.jpg]

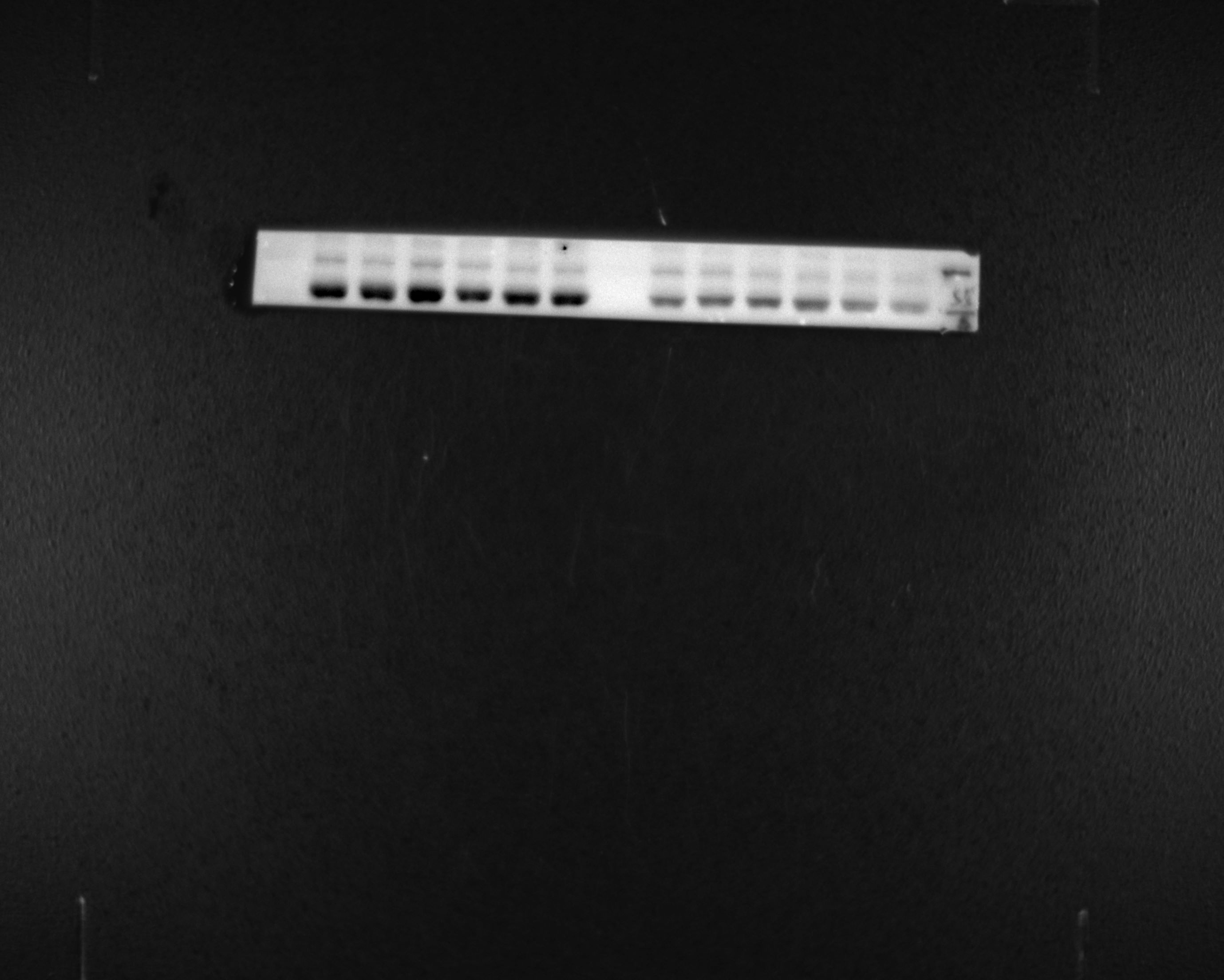

Supplement: Figure 4—source data 2. [file elife-101888-fig4-data2.zip › Figure 4B/FRMD8 2.jpg]

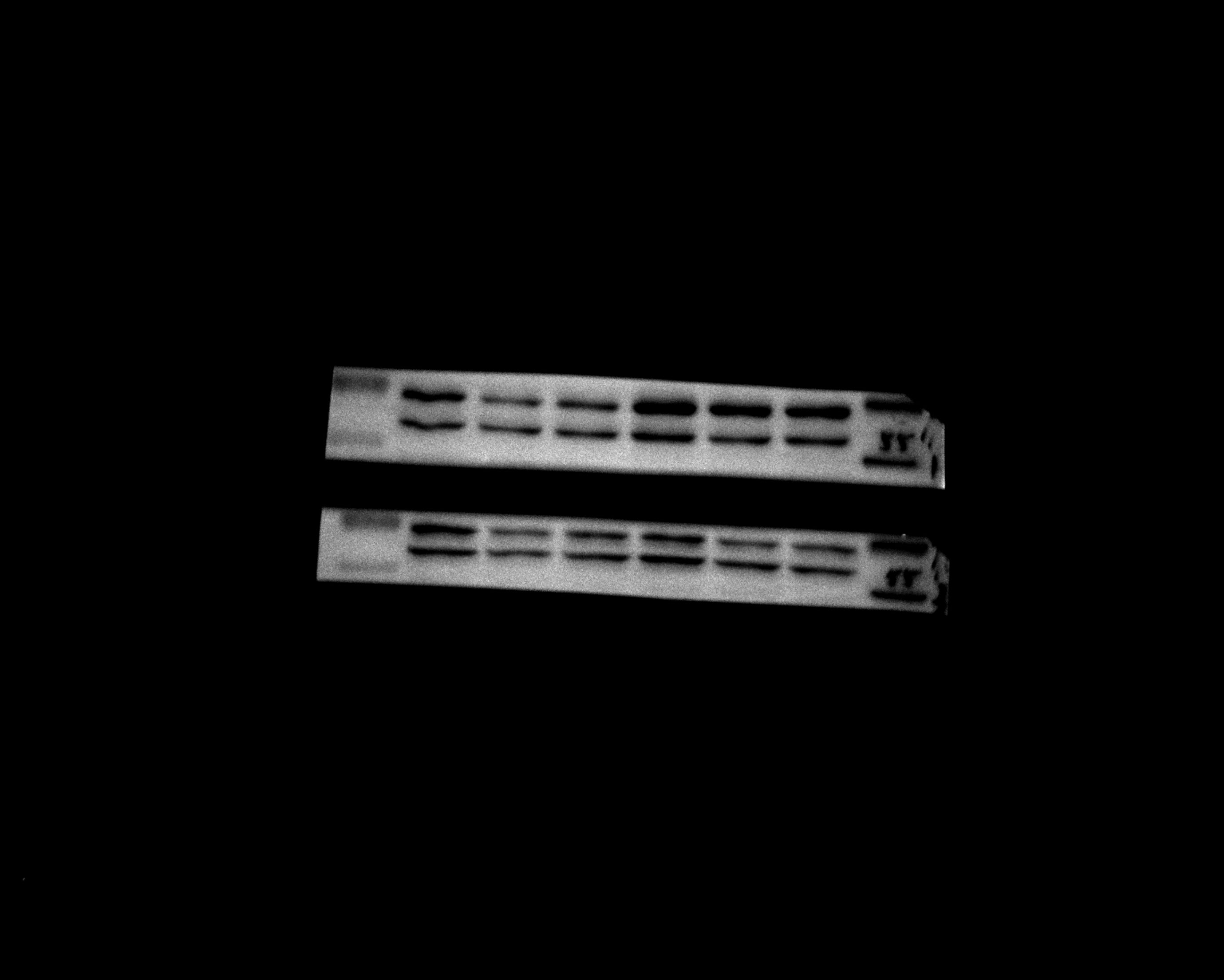

Supplement: Figure 4—source data 2. [file elife-101888-fig4-data2.zip › Figure 4D/ERα.jpg]

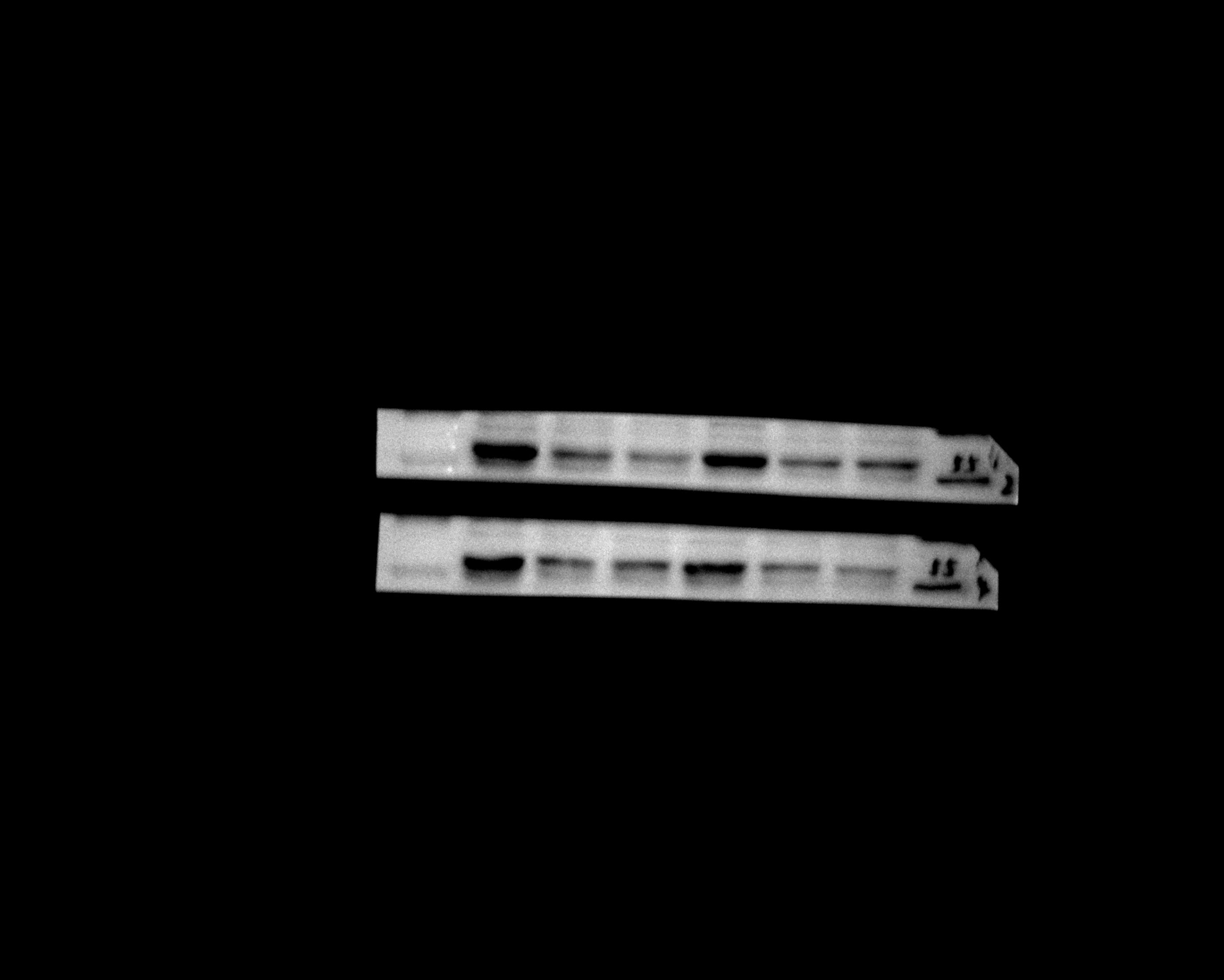

Supplement: Figure 4—source data 2. [file elife-101888-fig4-data2.zip › Figure 4D/FRMD8.jpg]

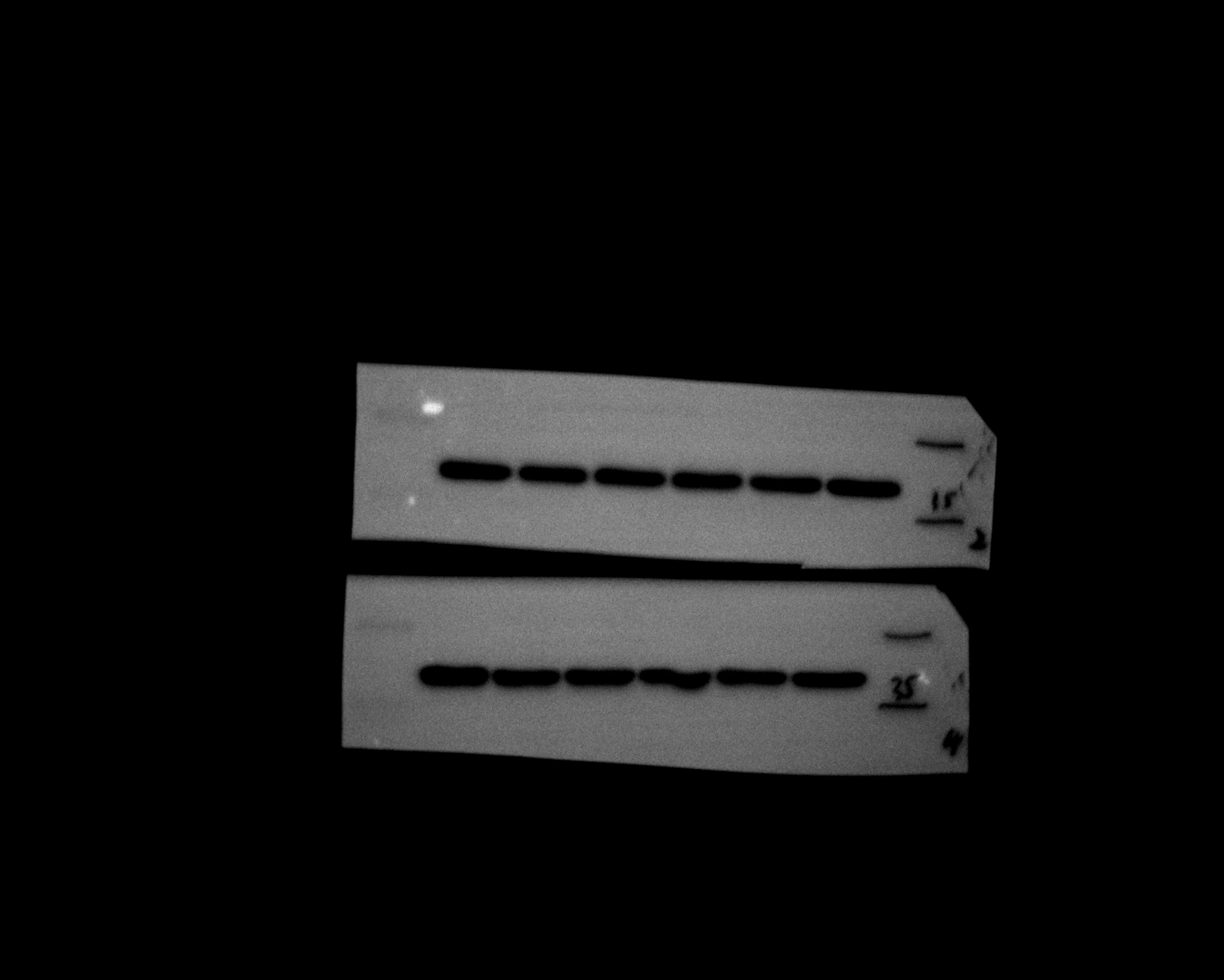

Supplement: Figure 4—source data 2. [file elife-101888-fig4-data2.zip › Figure 4D/GAPDH.jpg]

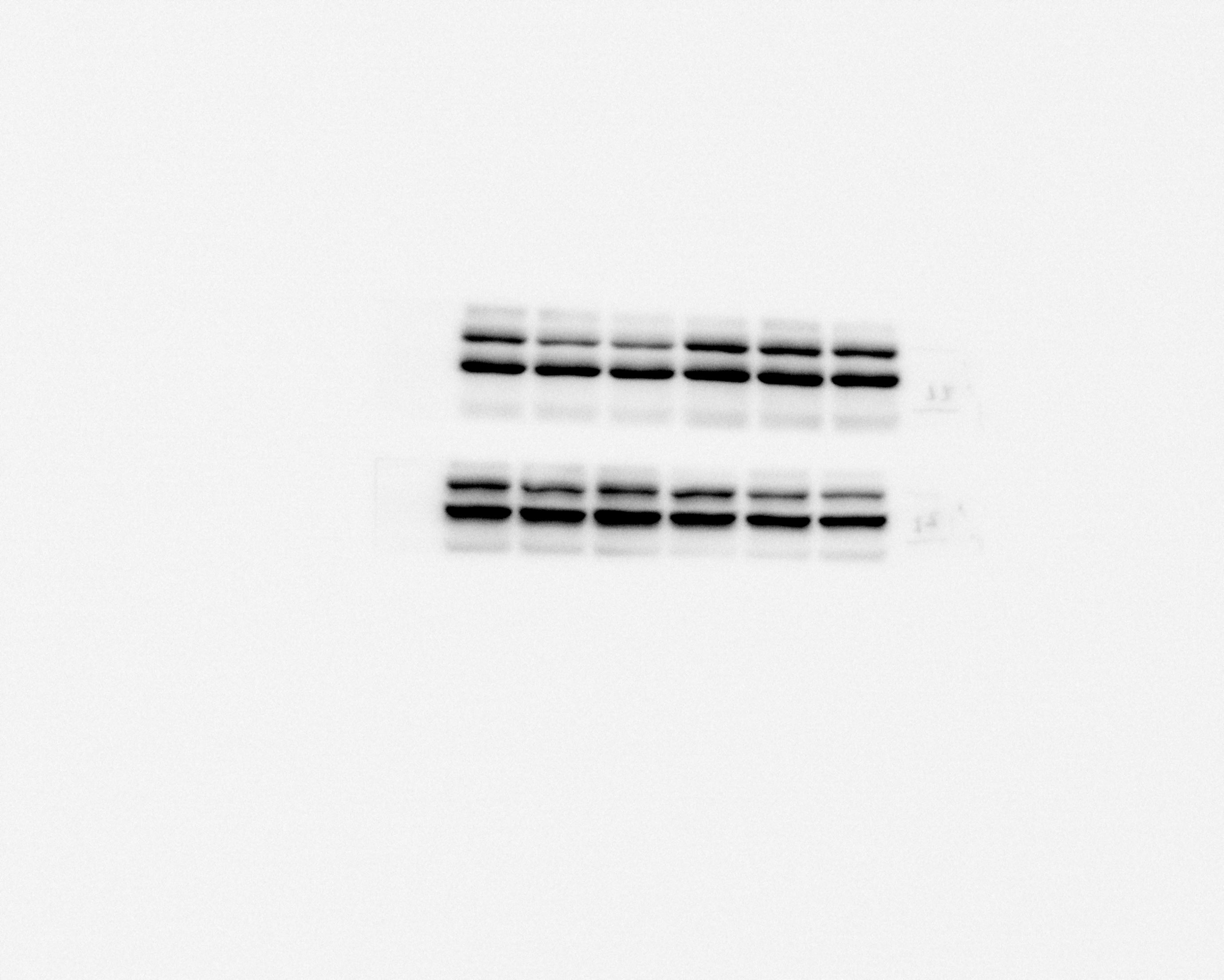

Supplement: Figure 4—source data 2. [file elife-101888-fig4-data2.zip › Figure 4E/ERα.jpg]

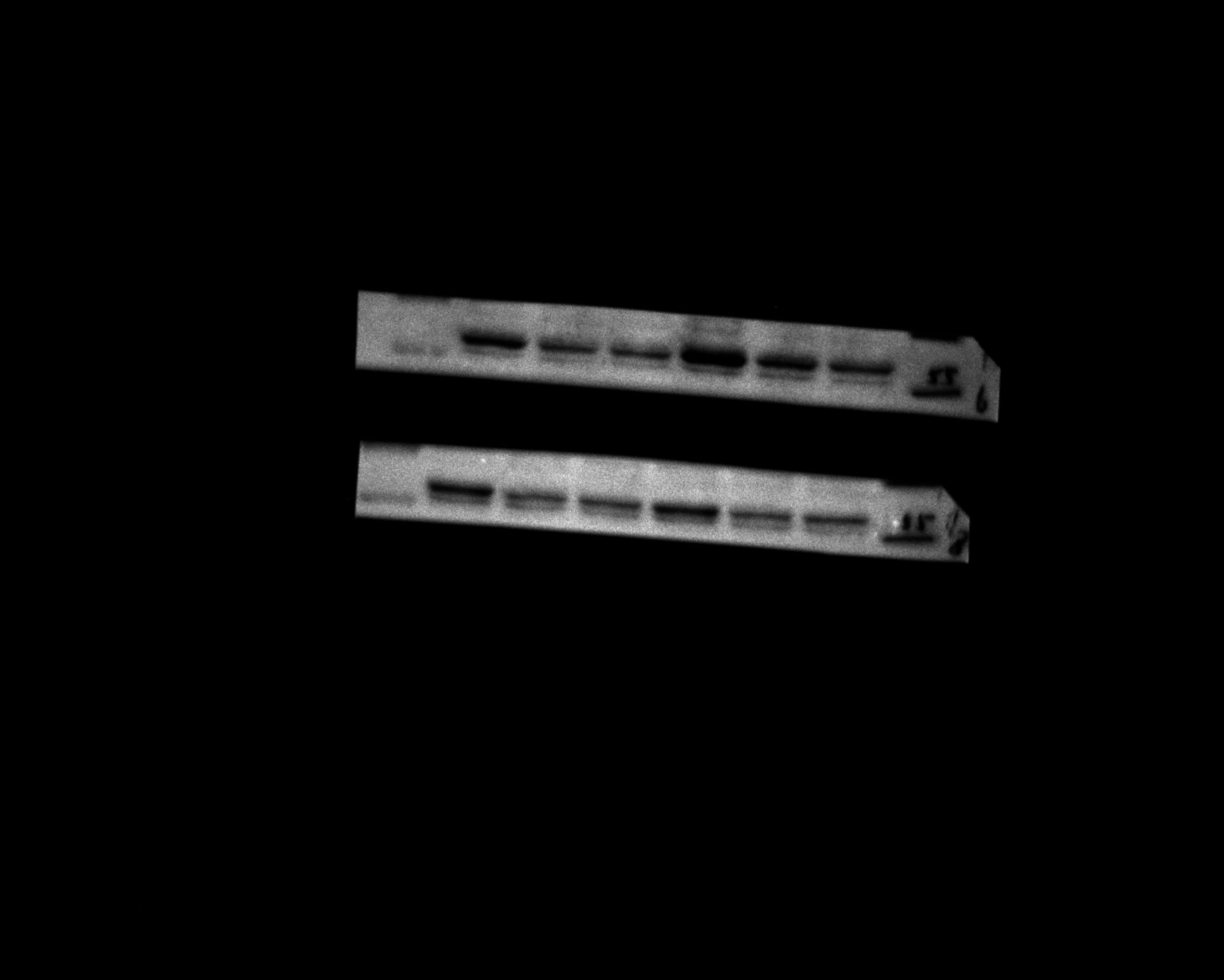

Supplement: Figure 4—source data 2. [file elife-101888-fig4-data2.zip › Figure 4E/FRMD8.jpg]

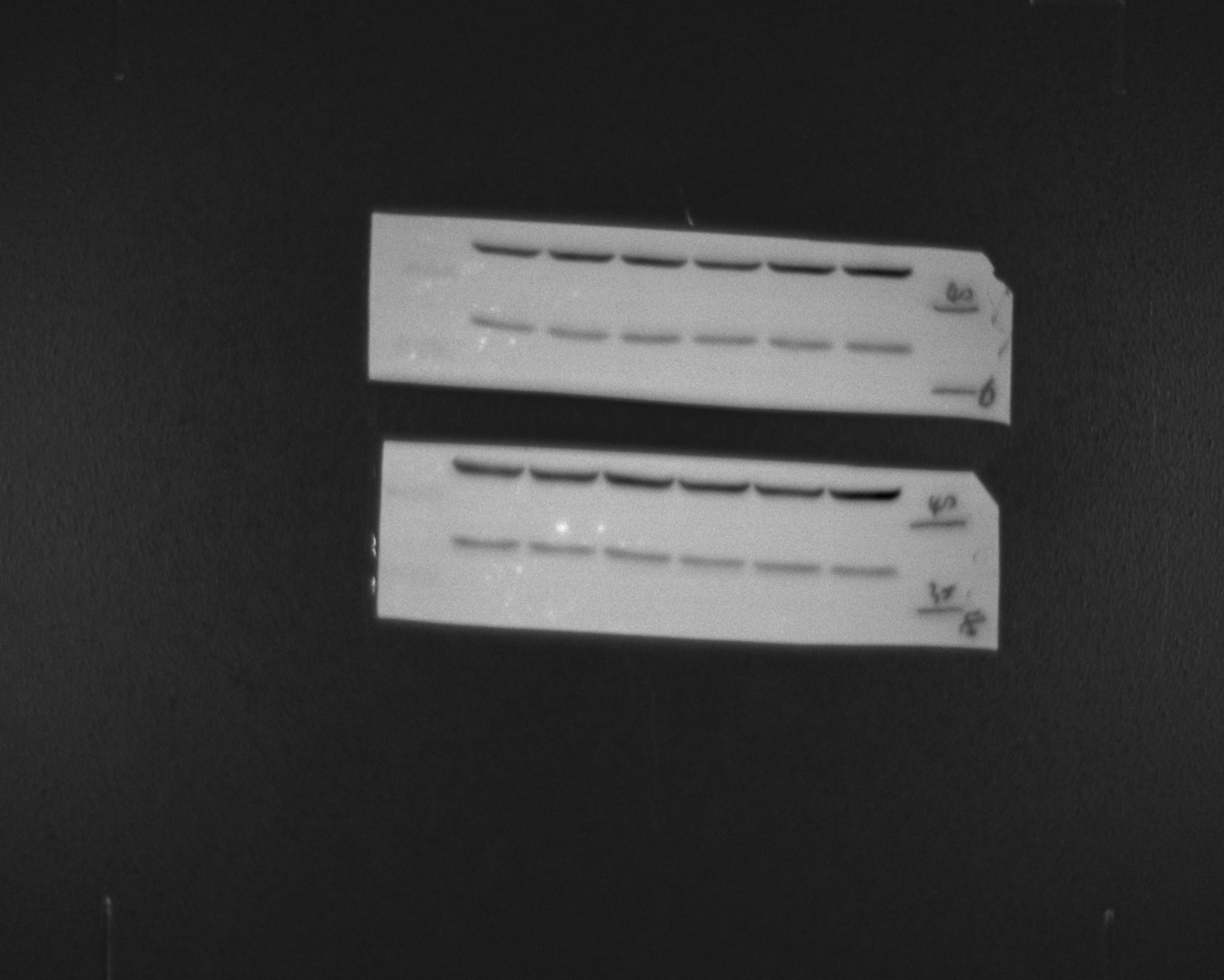

Supplement: Figure 4—source data 2. [file elife-101888-fig4-data2.zip › Figure 4E/GAPDH 1.jpg]

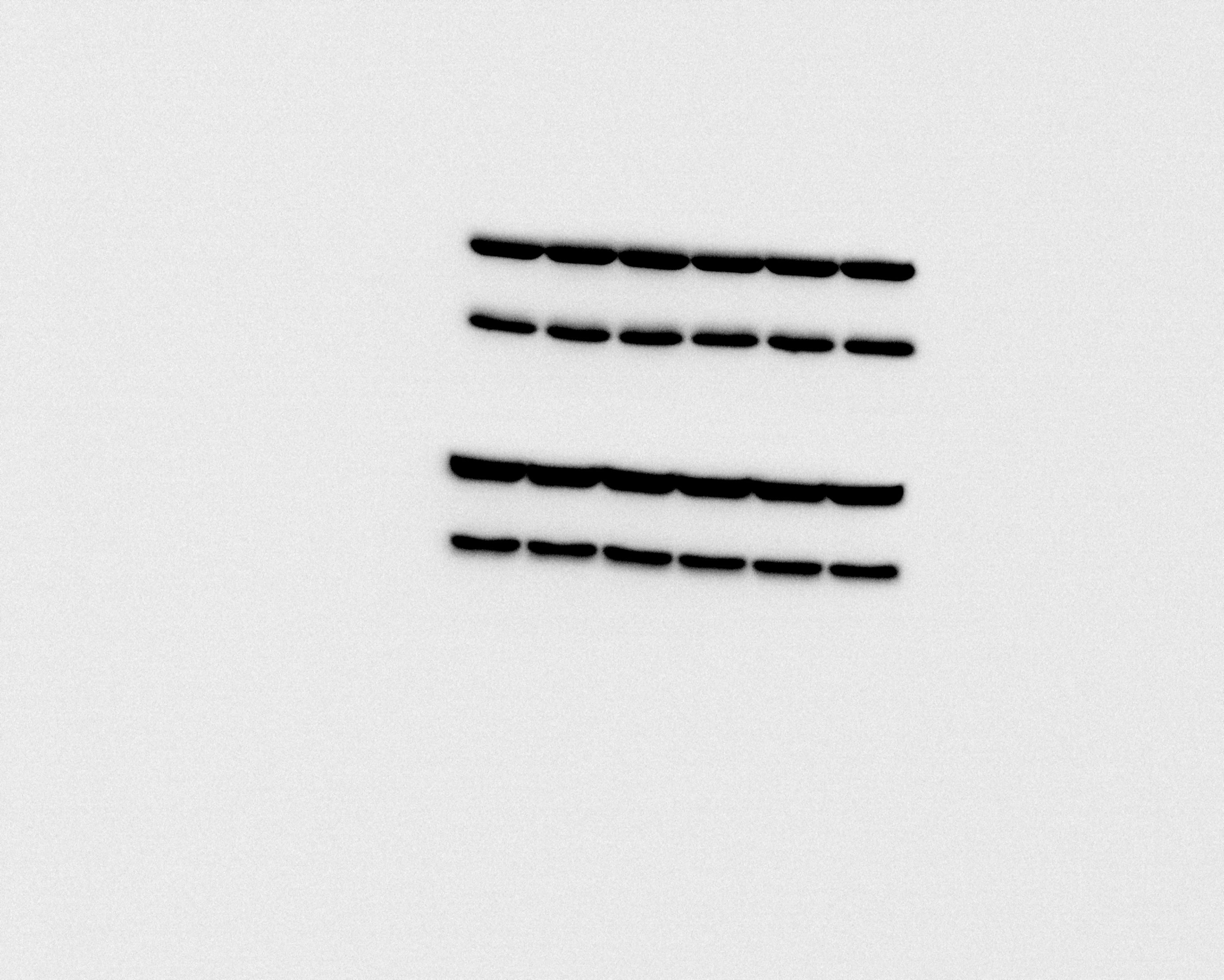

Supplement: Figure 4—source data 2. [file elife-101888-fig4-data2.zip › Figure 4E/GAPDH 2.jpg]

**Figure 5-source data:** Unedited western blot pictures for figure 5.

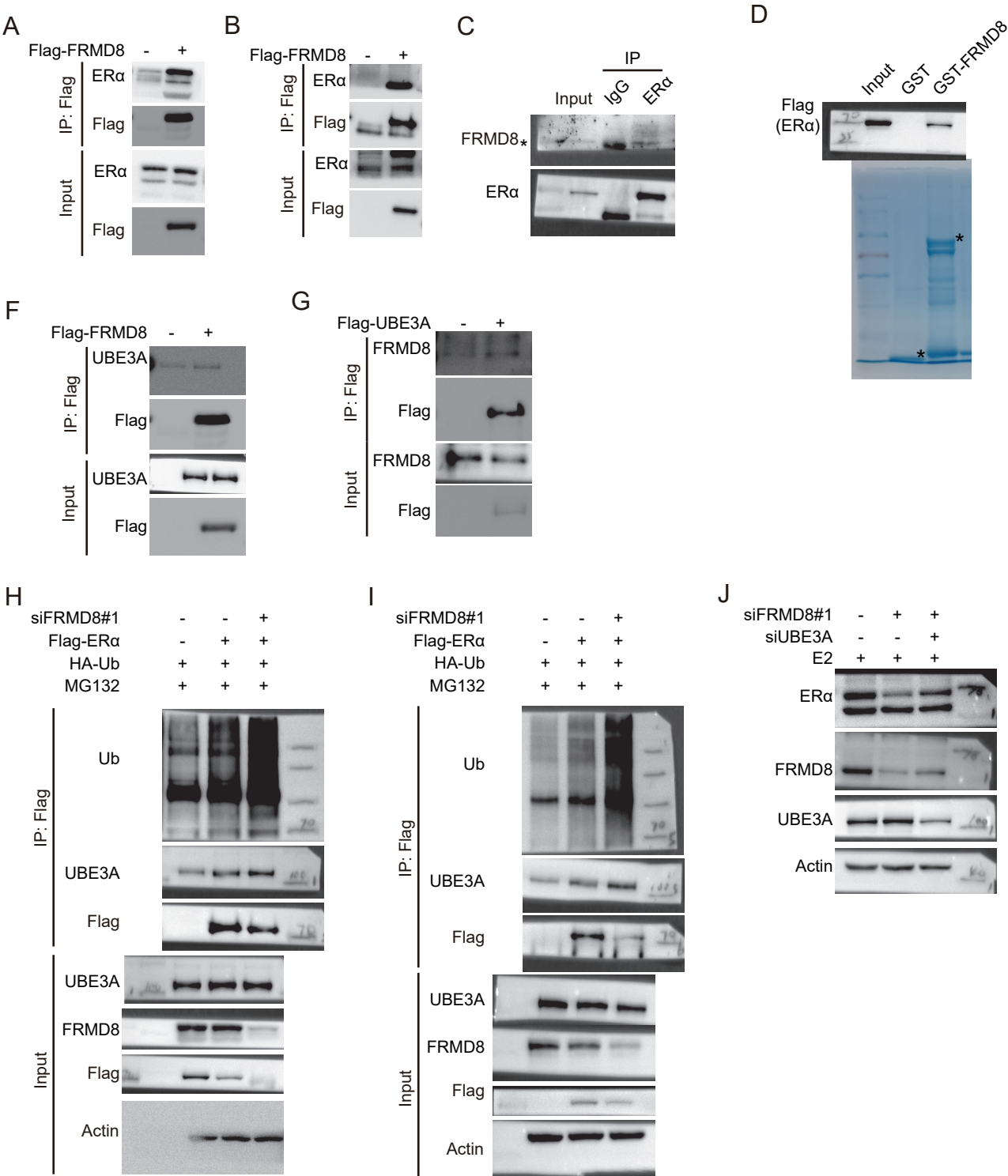

Supplement: Figure 5—source data 1. [file elife-101888-fig5-data1.pdf]

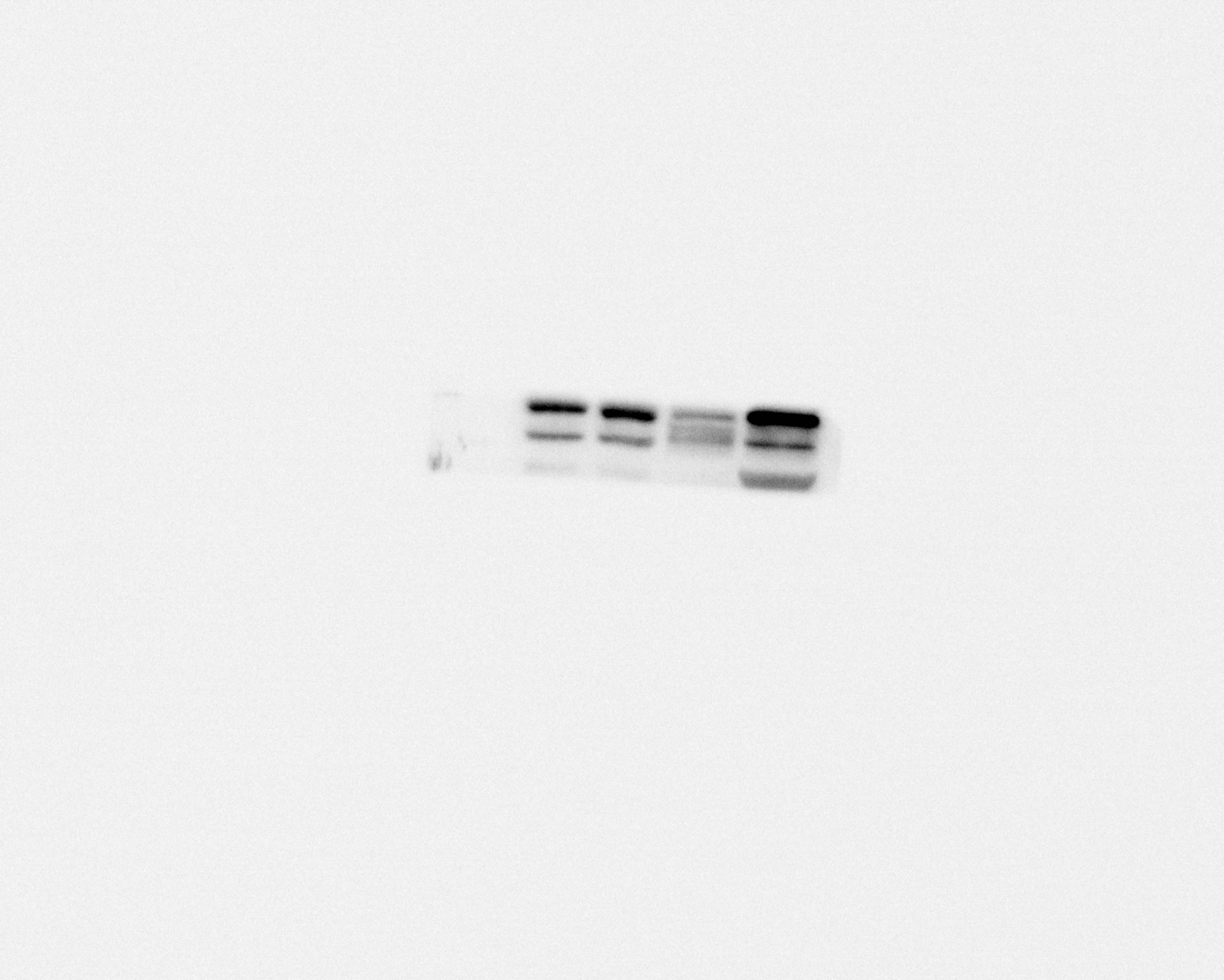

Supplement: Figure 5—source data 2. [file elife-101888-fig5-data2.zip › Figure 5A/ERα.jpg]

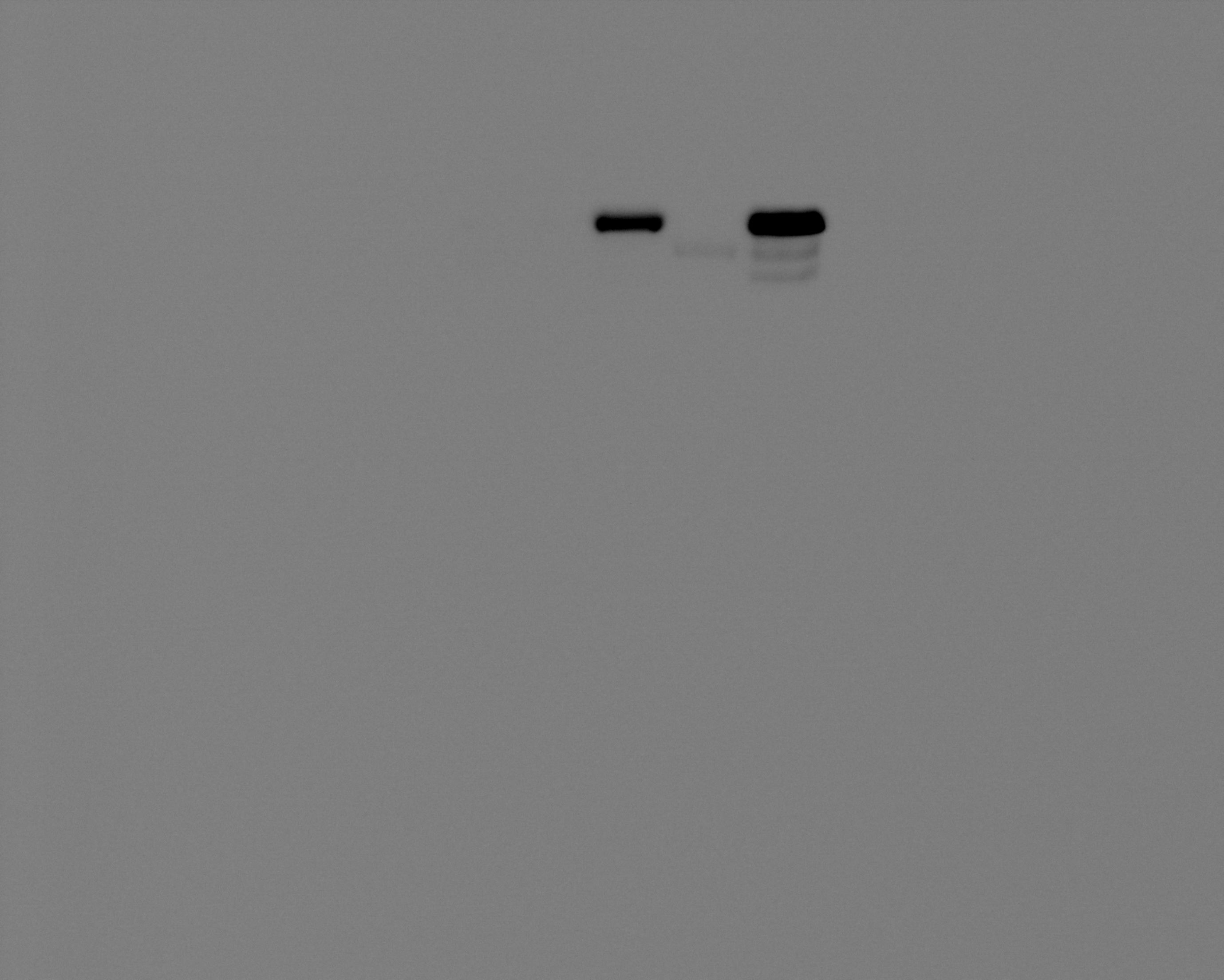

Supplement: Figure 5—source data 2. [file elife-101888-fig5-data2.zip › Figure 5A/Flag.jpg]

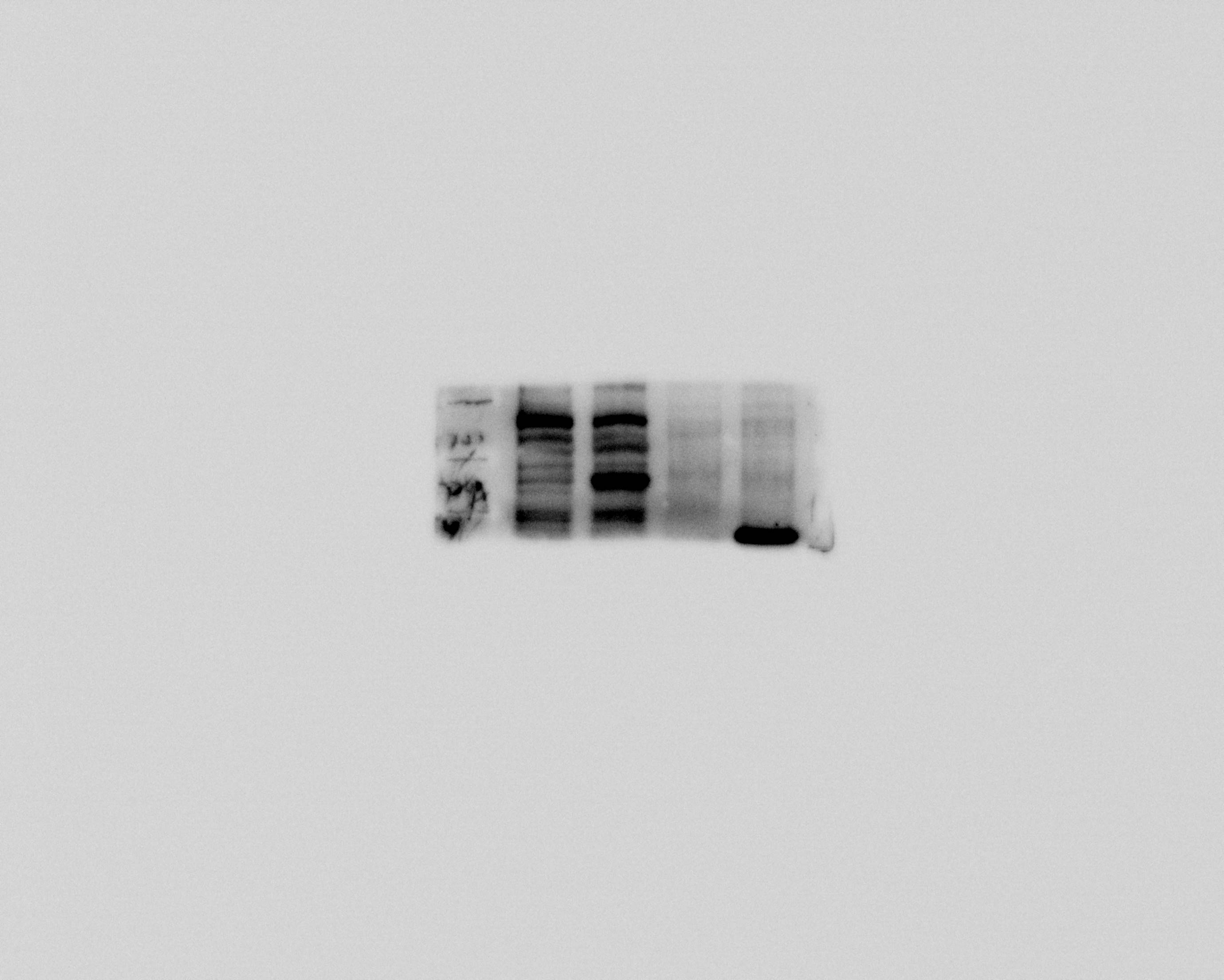

Supplement: Figure 5—source data 2. [file elife-101888-fig5-data2.zip › Figure 5B/ERα.jpg]

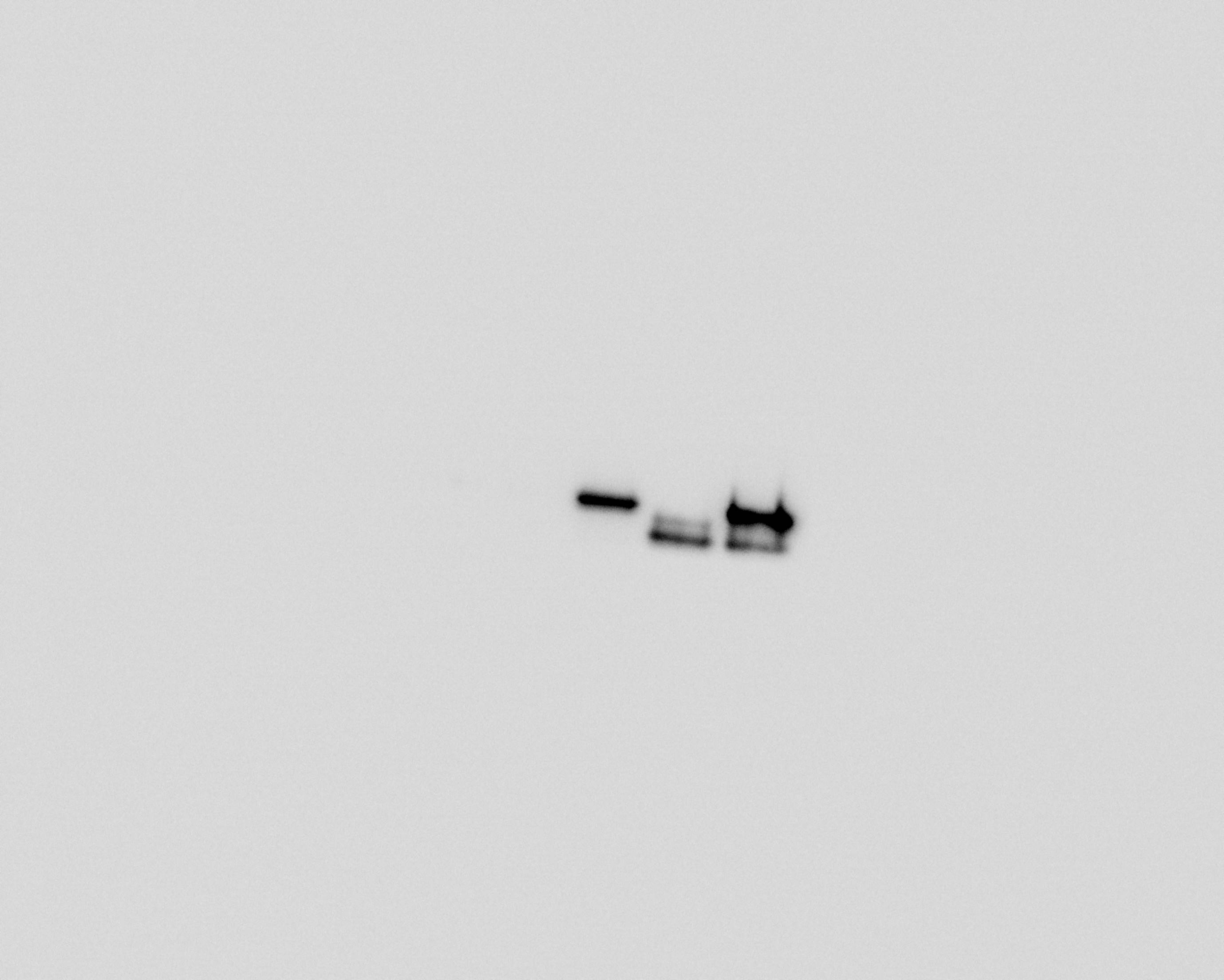

Supplement: Figure 5—source data 2. [file elife-101888-fig5-data2.zip › Figure 5B/Flag.jpg]

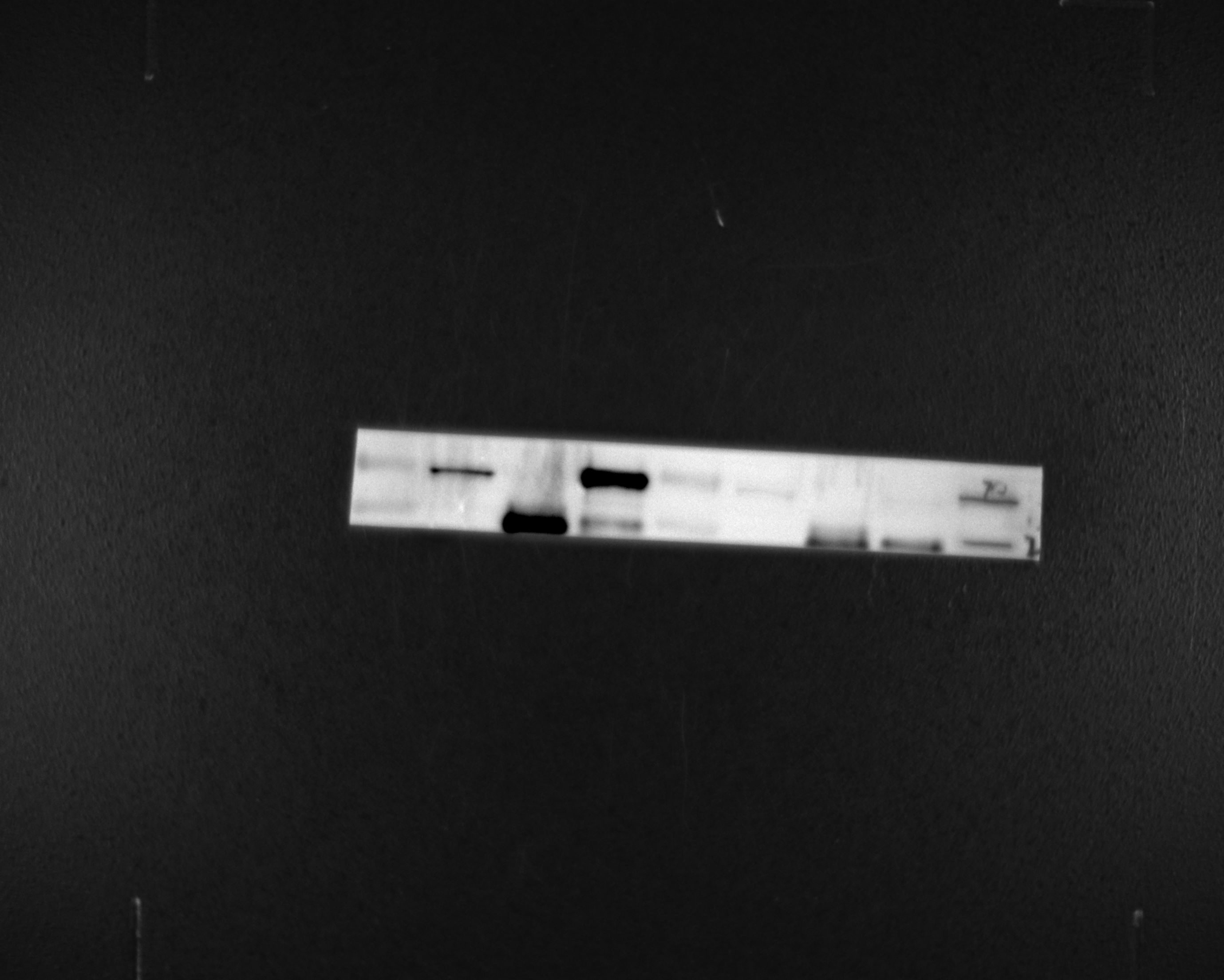

Supplement: Figure 5—source data 2. [file elife-101888-fig5-data2.zip › Figure 5C/ERα.jpg]

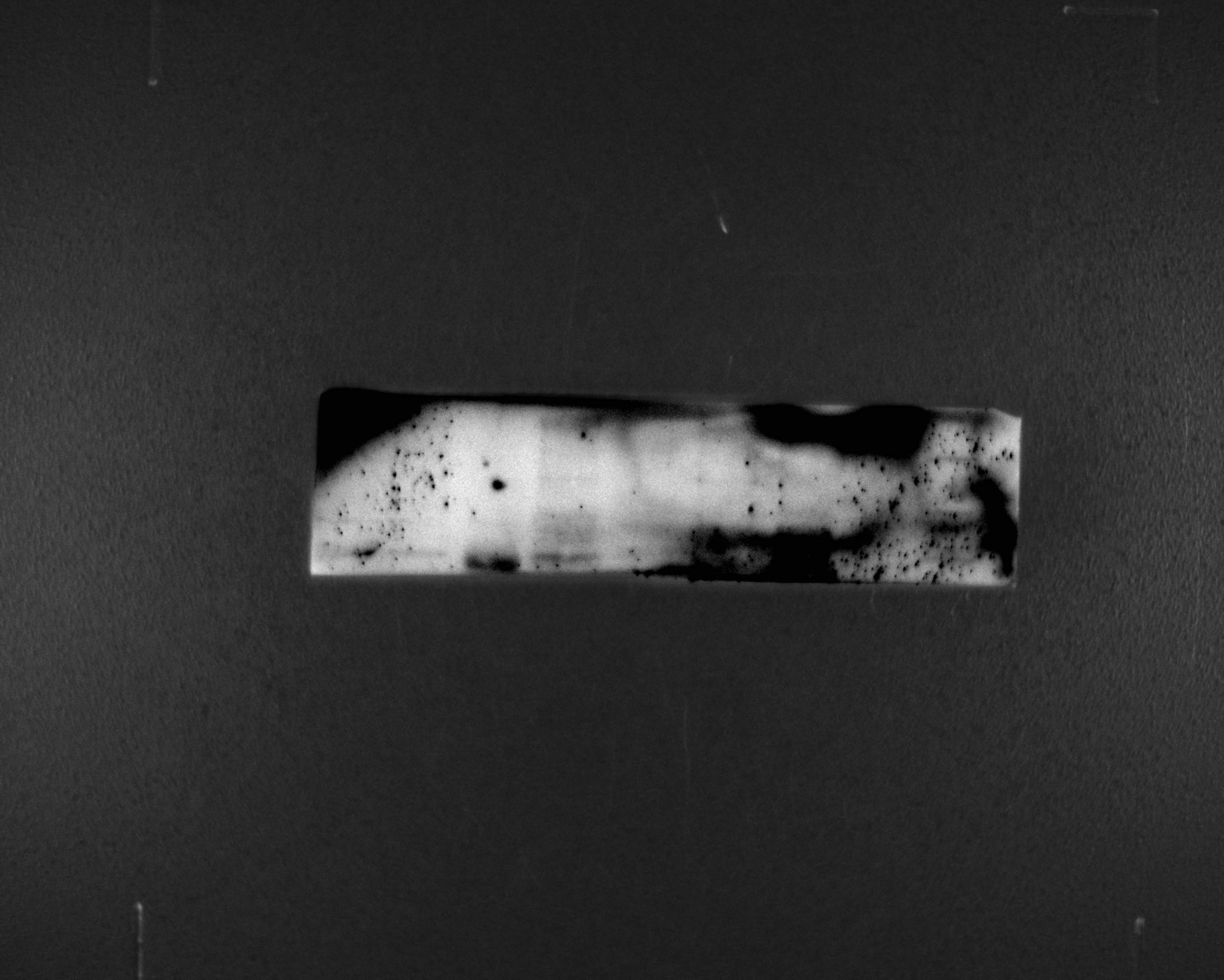

Supplement: Figure 5—source data 2. [file elife-101888-fig5-data2.zip › Figure 5C/FRMD8.jpg]

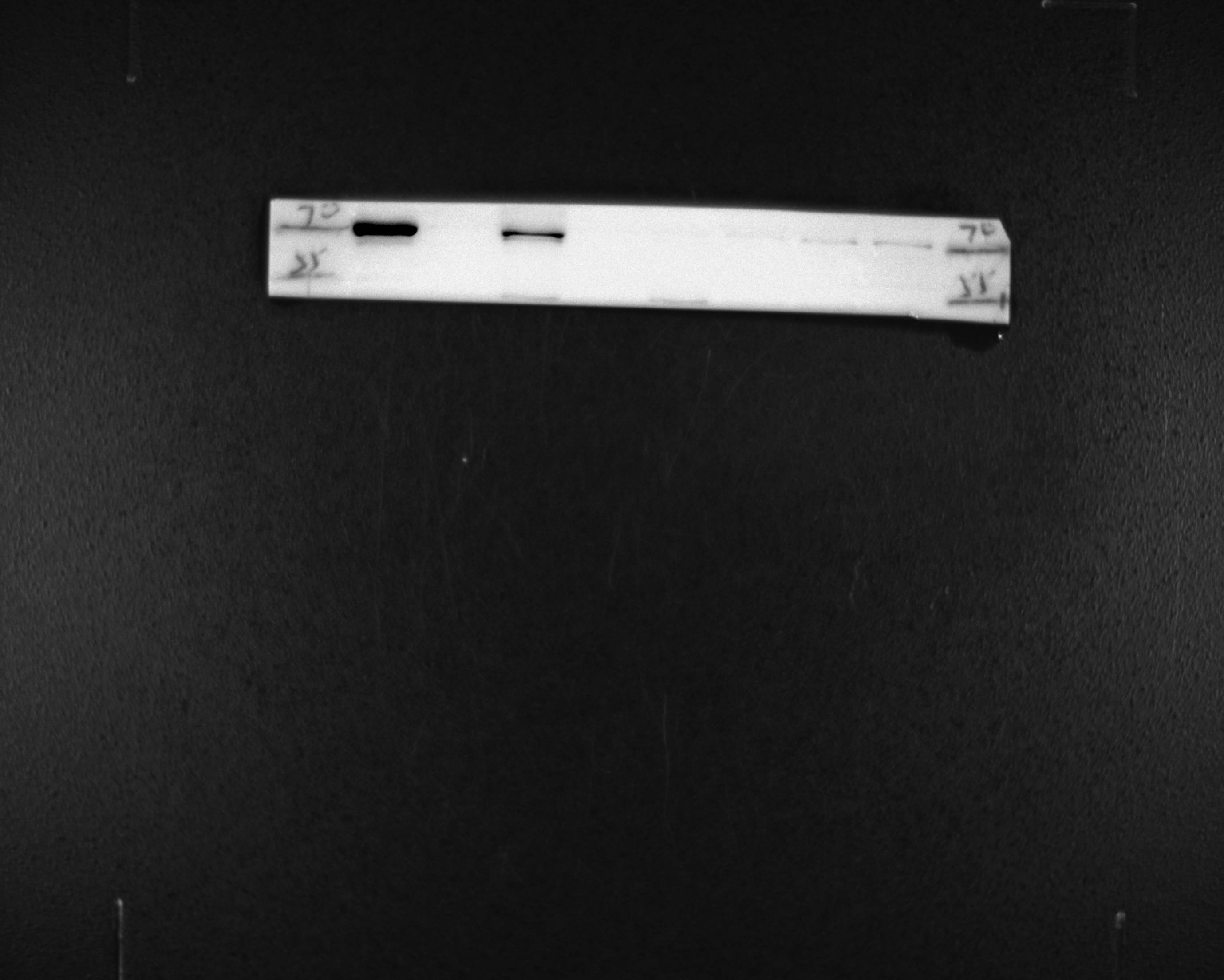

Supplement: Figure 5—source data 2. [file elife-101888-fig5-data2.zip › Figure 5D/Flag.jpg]

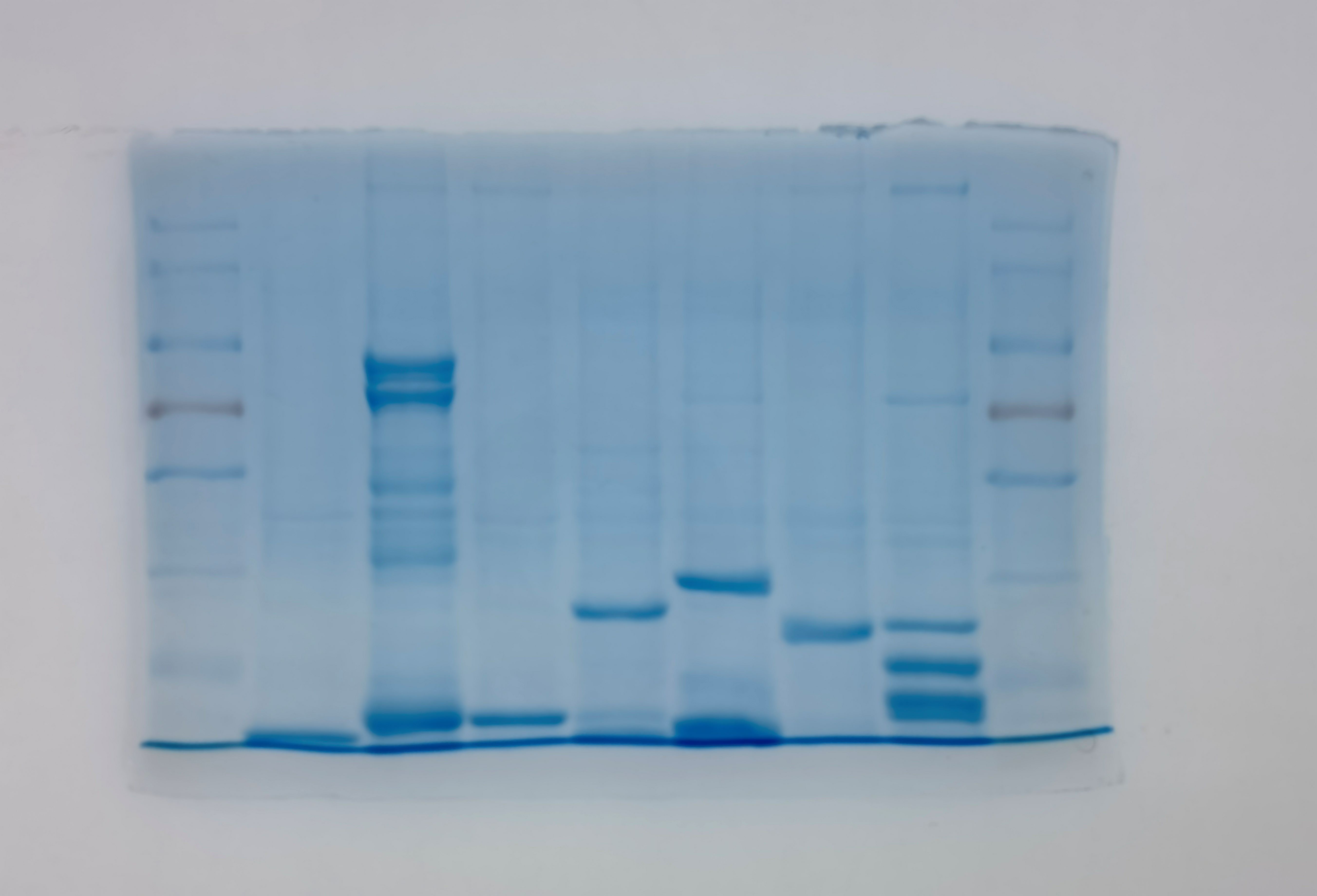

Supplement: Figure 5—source data 2. [file elife-101888-fig5-data2.zip › Figure 5D/GST-F8.jpg]

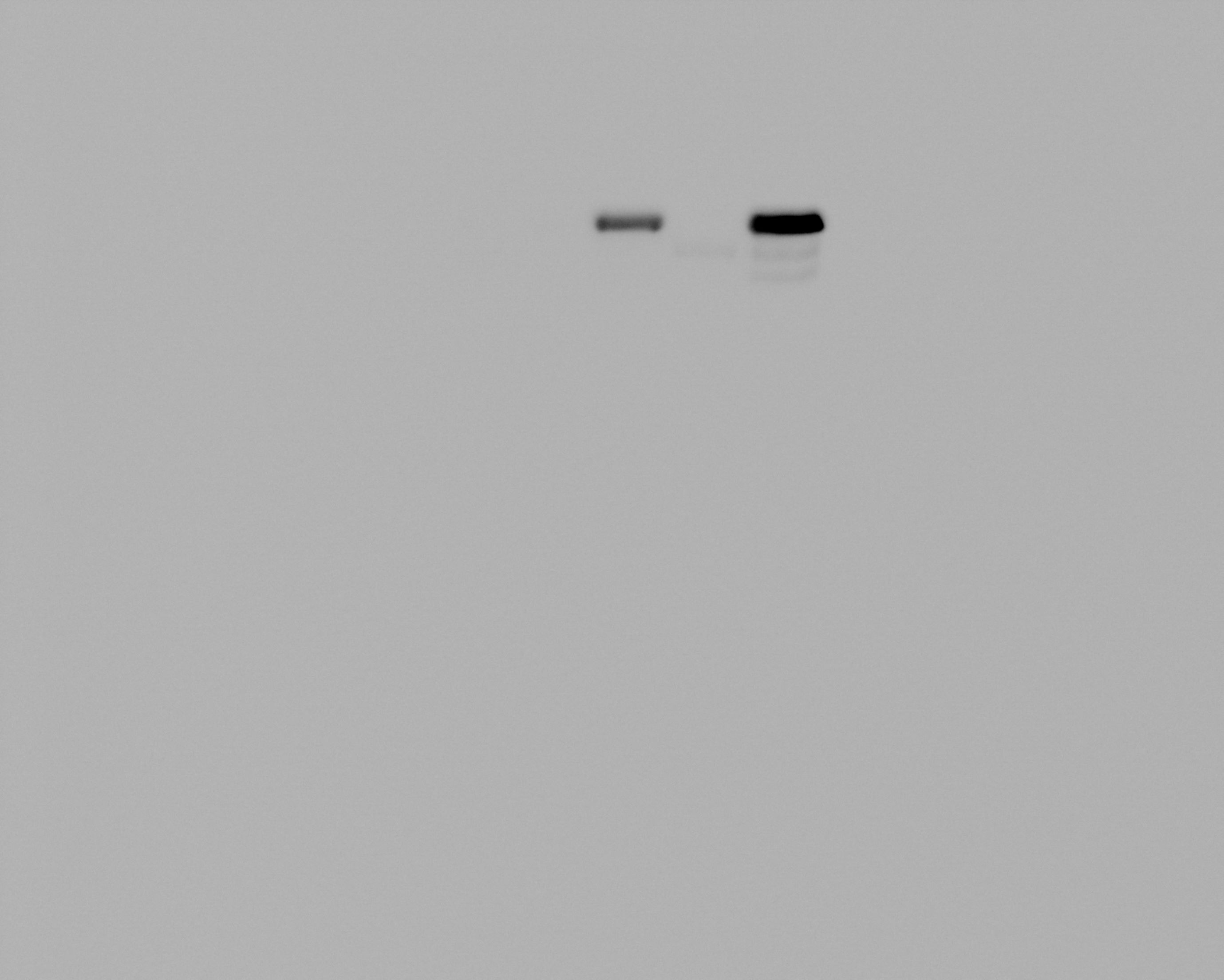

Supplement: Figure 5—source data 2. [file elife-101888-fig5-data2.zip › Figure 5F/Flag.jpg]

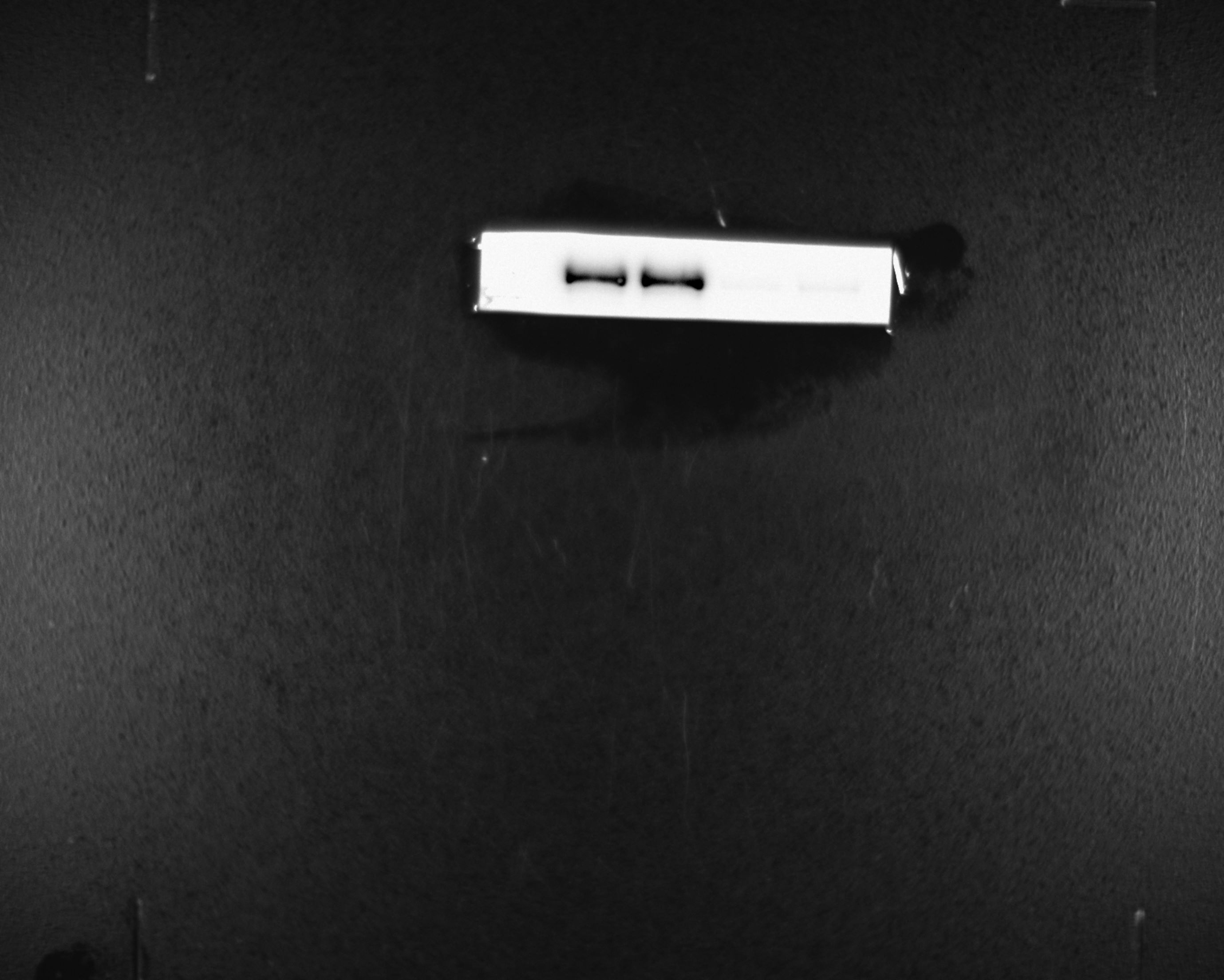

Supplement: Figure 5—source data 2. [file elife-101888-fig5-data2.zip › Figure 5F/UBE3A input.jpg]

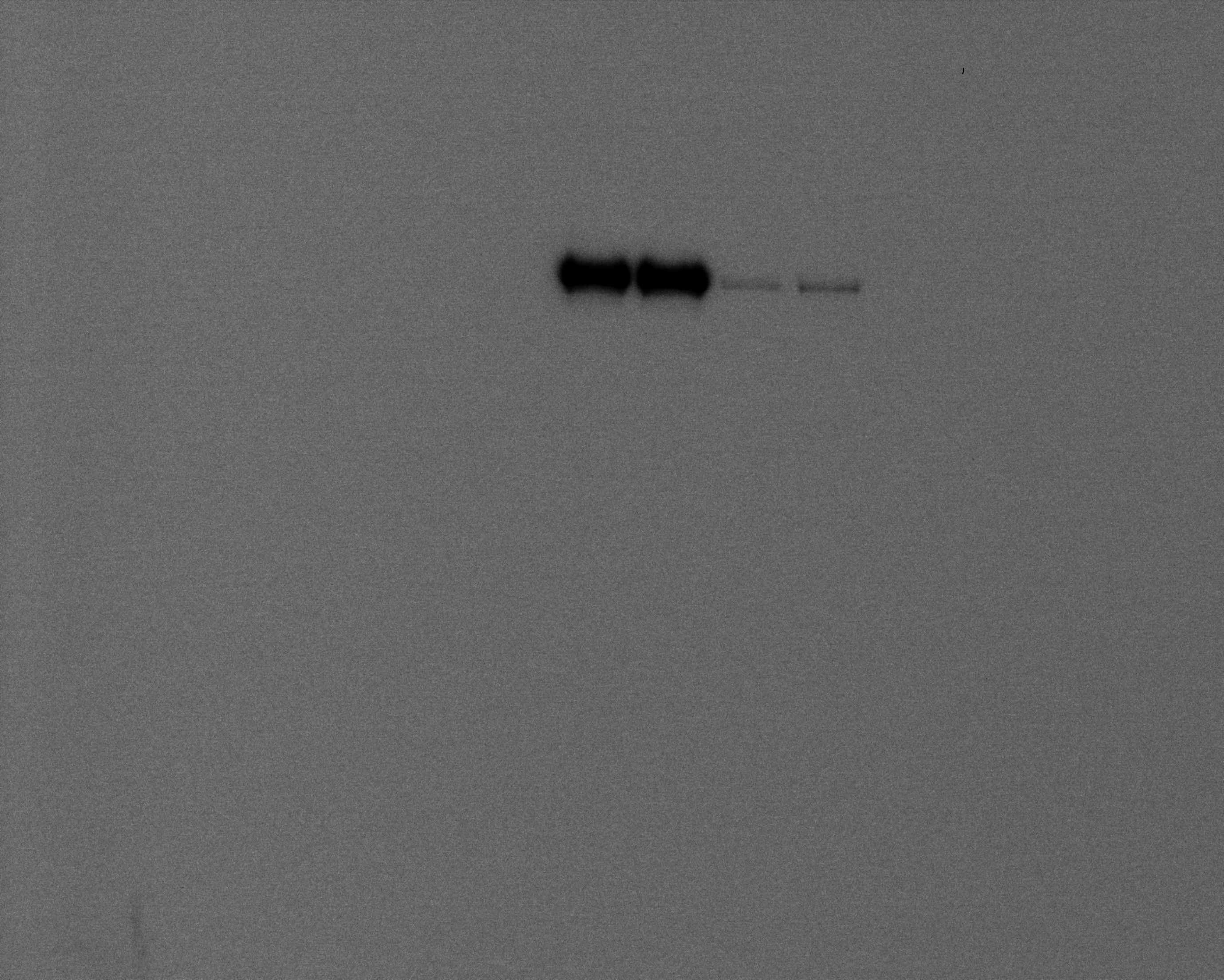

Supplement: Figure 5—source data 2. [file elife-101888-fig5-data2.zip › Figure 5F/UBE3A IP.jpg]

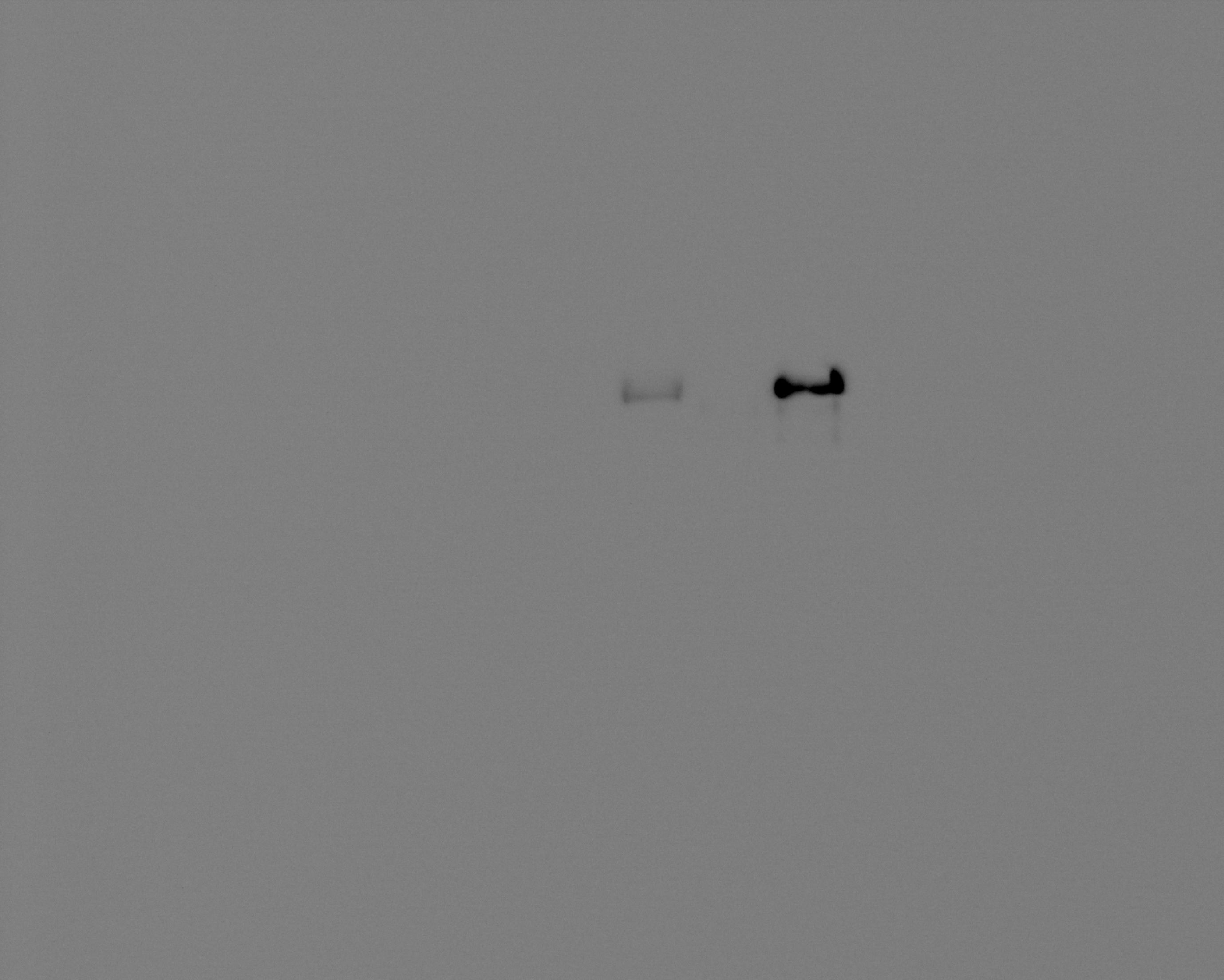

Supplement: Figure 5—source data 2. [file elife-101888-fig5-data2.zip › Figure 5G/Flag.jpg]

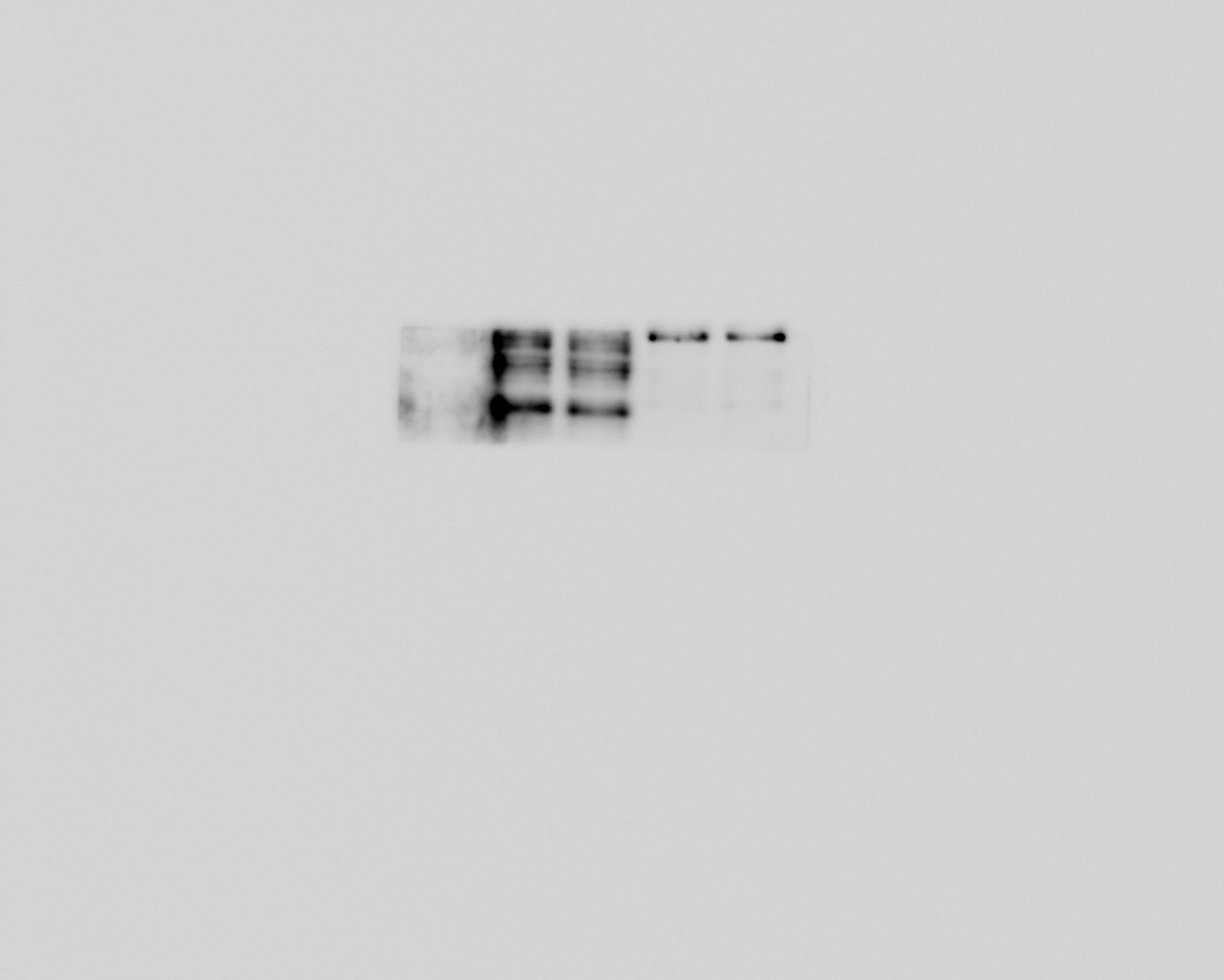

Supplement: Figure 5—source data 2. [file elife-101888-fig5-data2.zip › Figure 5G/FRMD8 input.jpg]

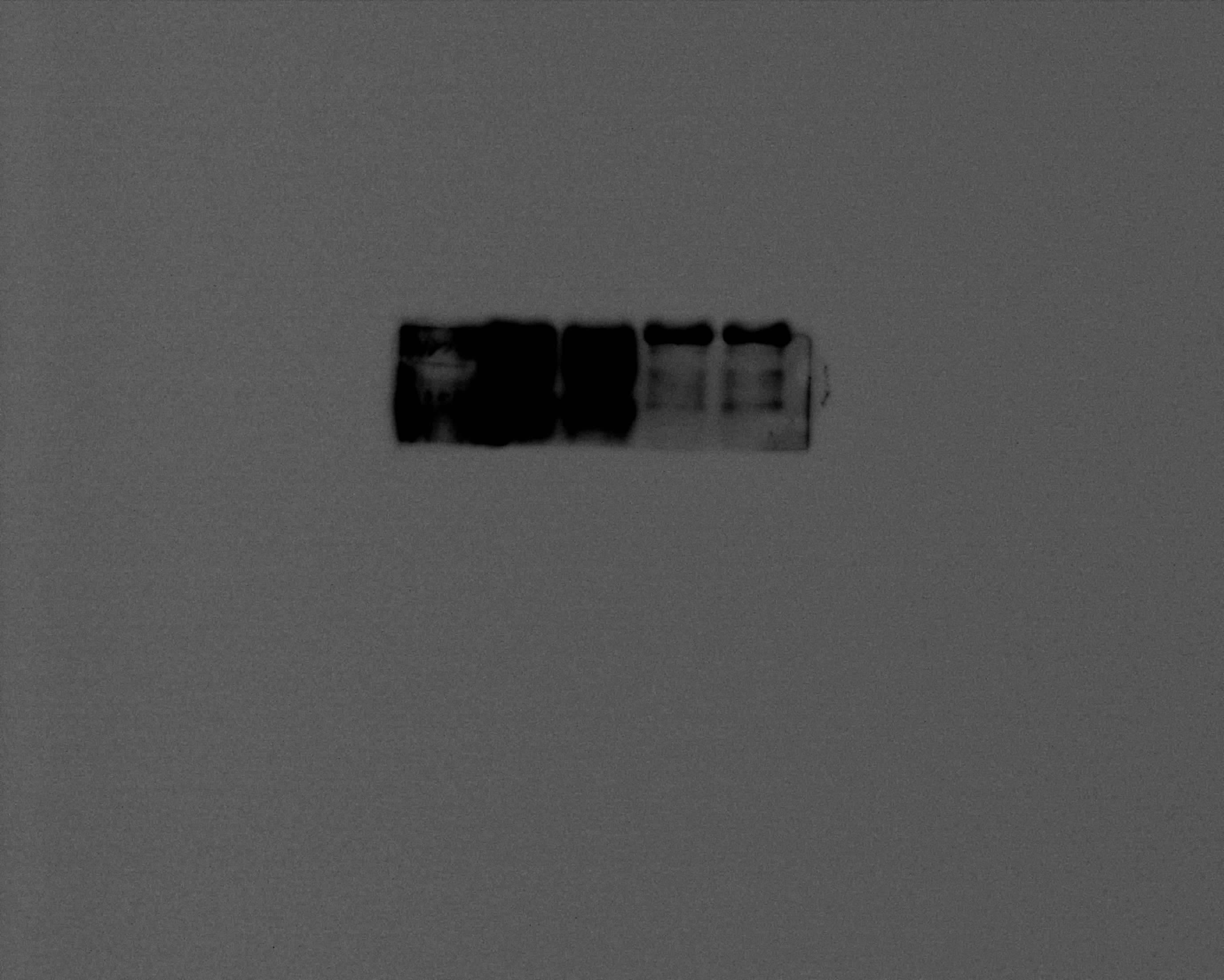

Supplement: Figure 5—source data 2. [file elife-101888-fig5-data2.zip › Figure 5G/FRMD8 IP.jpg]

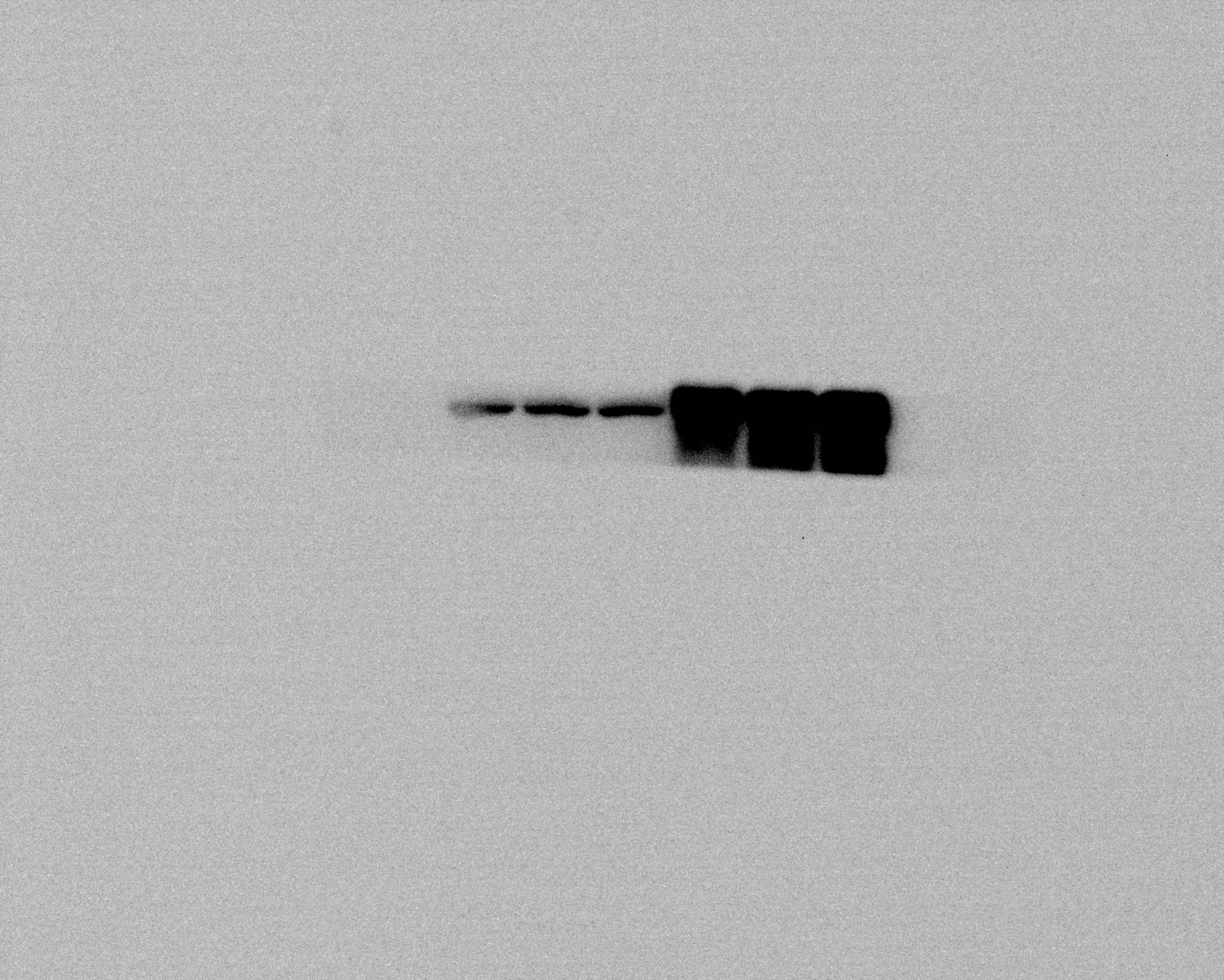

Supplement: Figure 5—source data 2. [file elife-101888-fig5-data2.zip › Figure 5H/Actin.jpg]

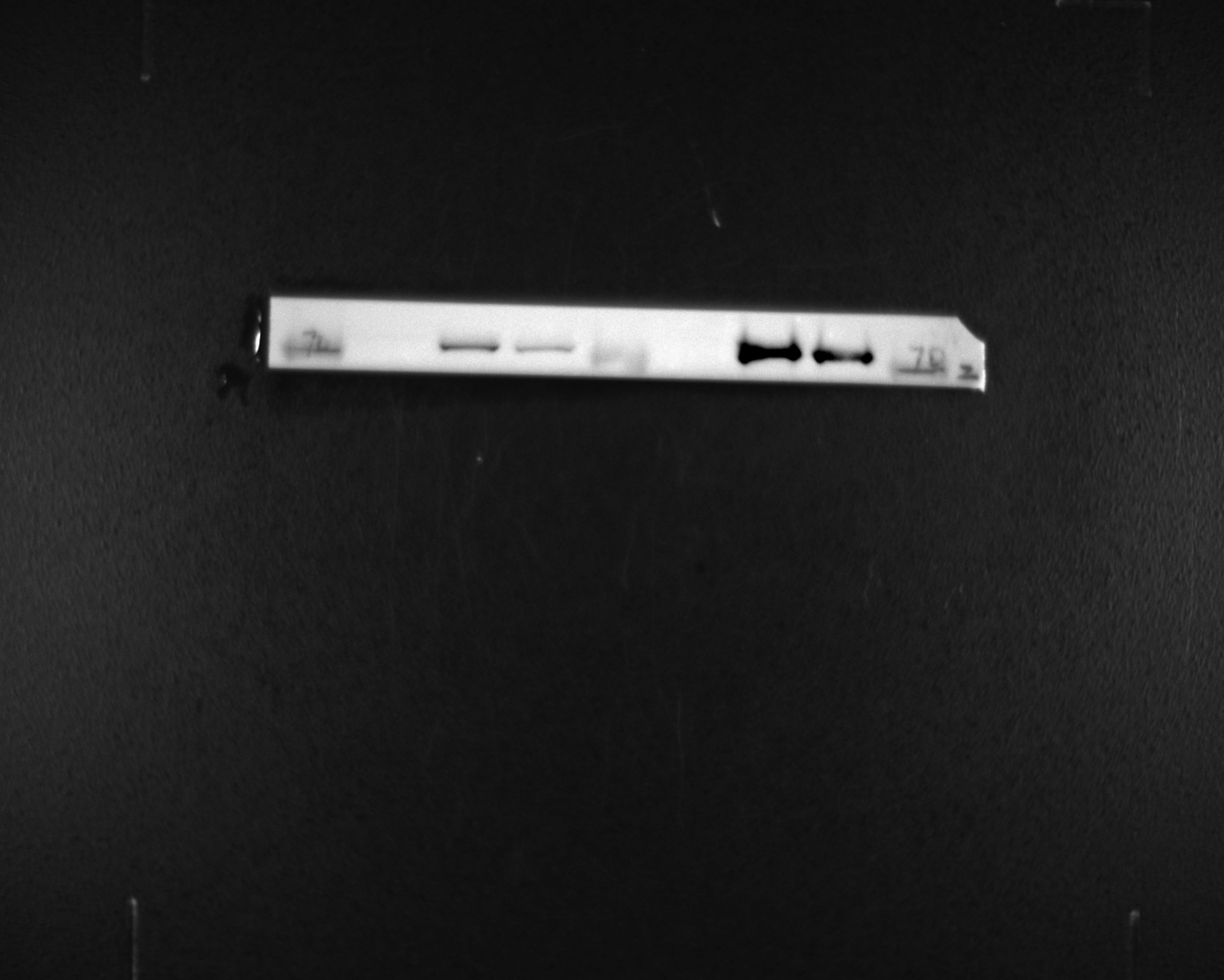

Supplement: Figure 5—source data 2. [file elife-101888-fig5-data2.zip › Figure 5H/Flag.jpg]

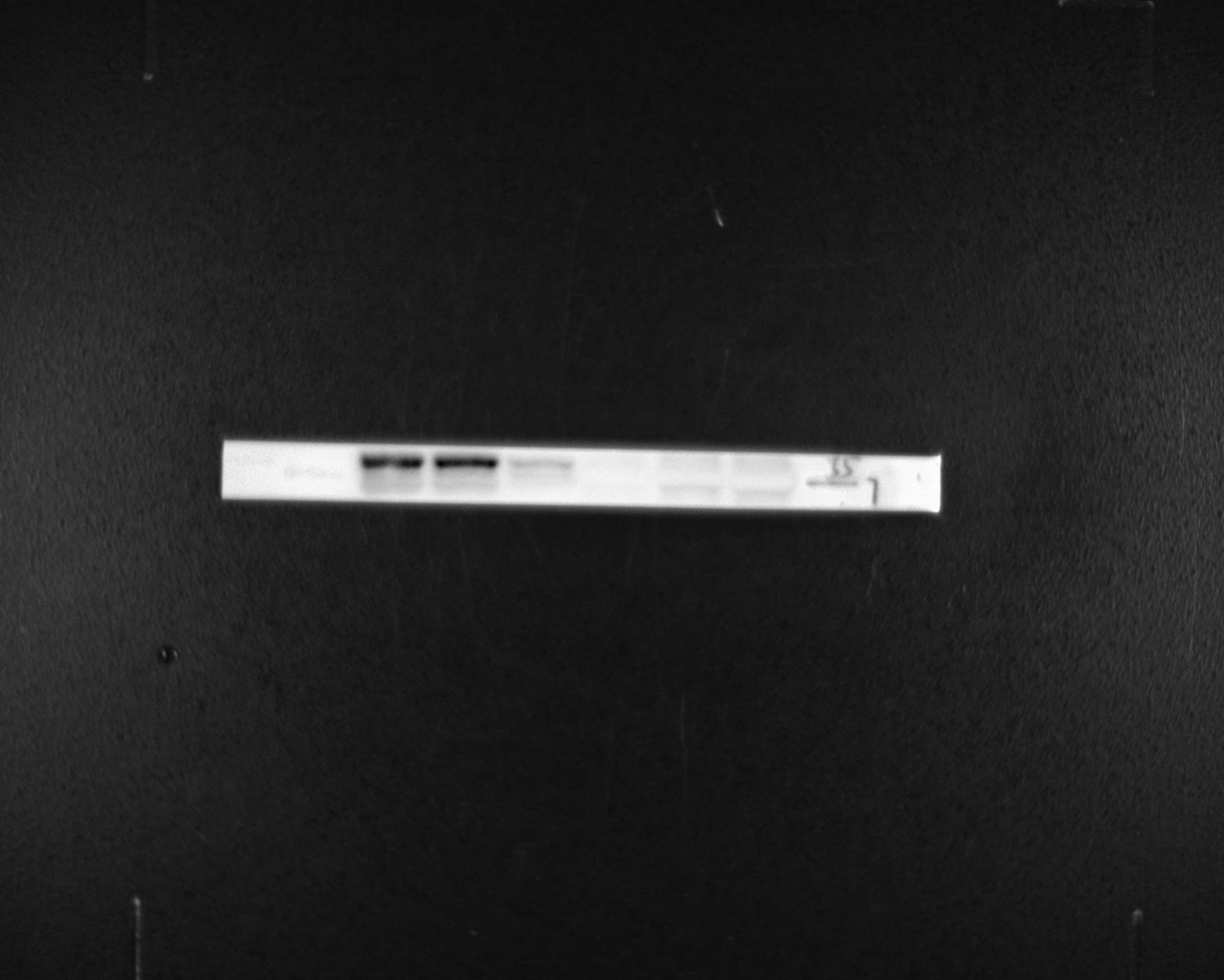

Supplement: Figure 5—source data 2. [file elife-101888-fig5-data2.zip › Figure 5H/FRMD8.jpg]

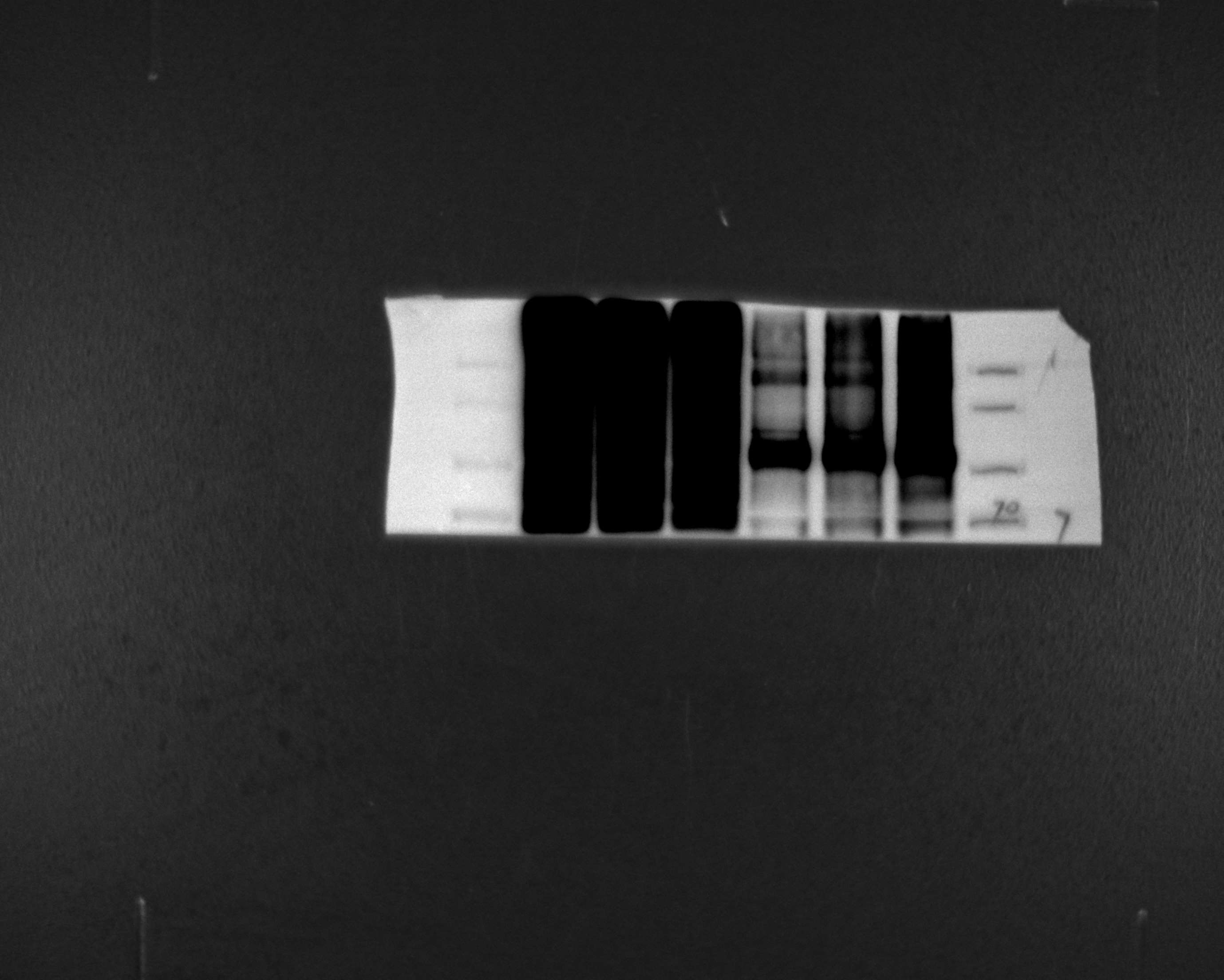

Supplement: Figure 5—source data 2. [file elife-101888-fig5-data2.zip › Figure 5H/Ub.jpg]

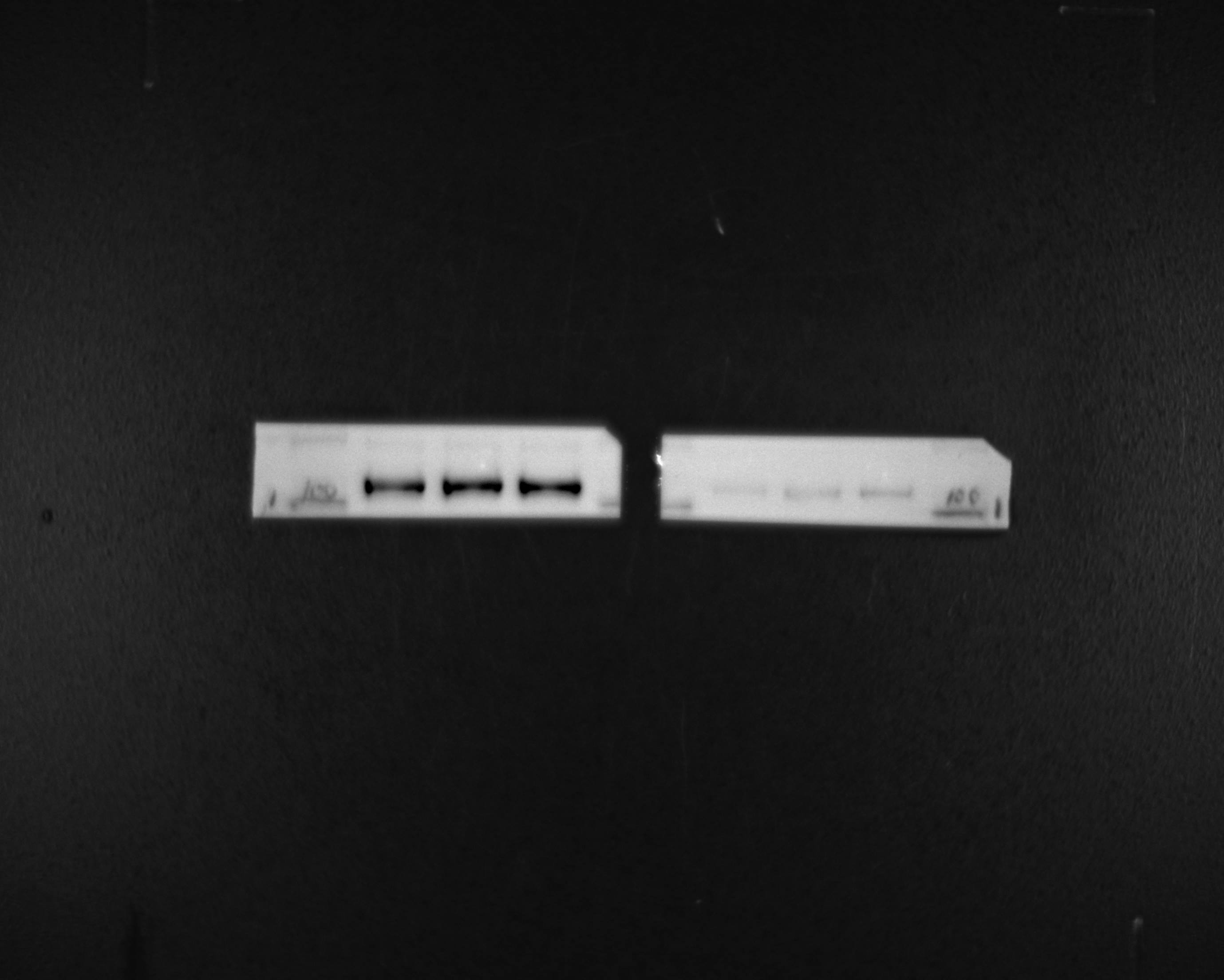

Supplement: Figure 5—source data 2. [file elife-101888-fig5-data2.zip › Figure 5H/UBE3A input.jpg]

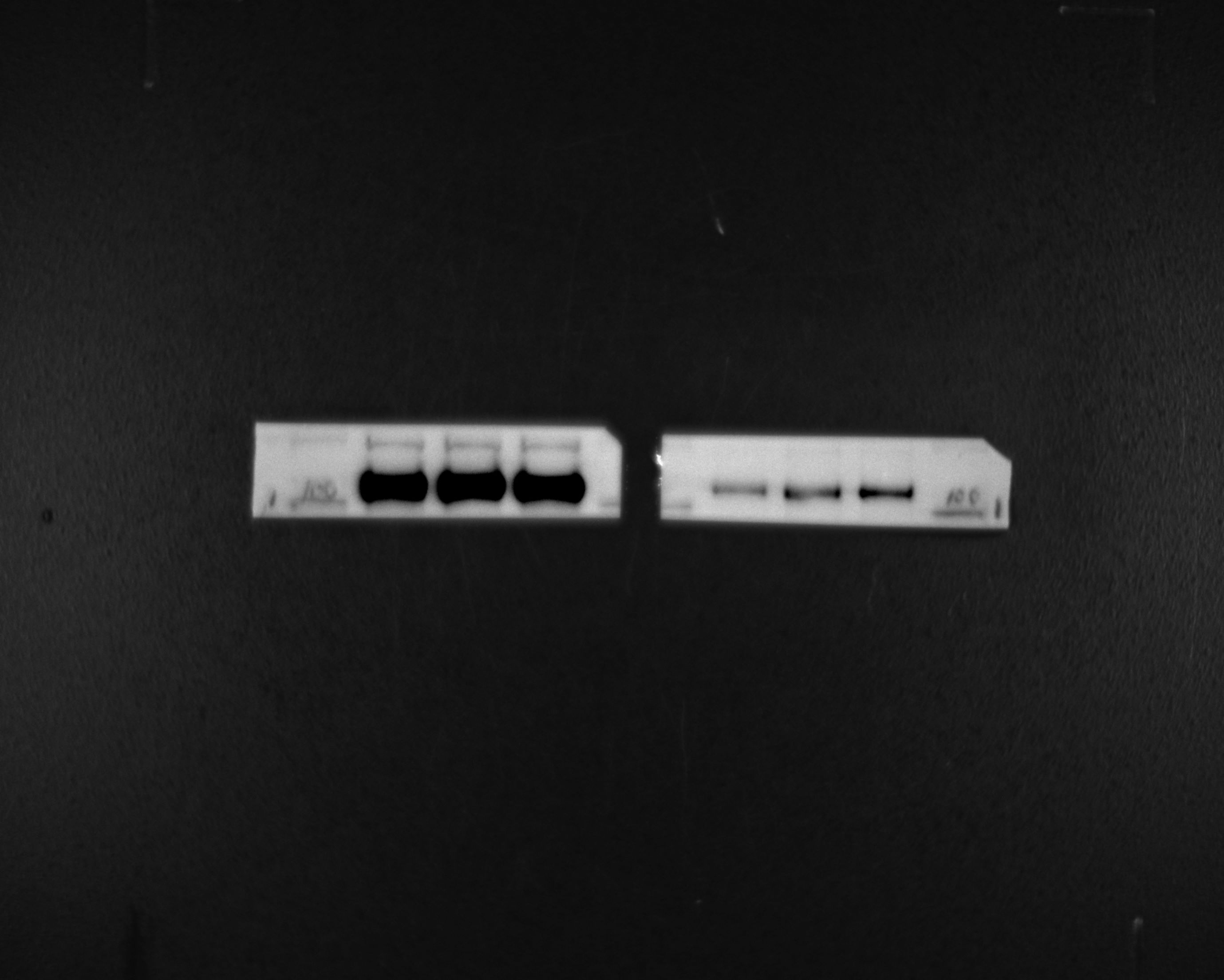

Supplement: Figure 5—source data 2. [file elife-101888-fig5-data2.zip › Figure 5H/UBE3A IP.jpg]

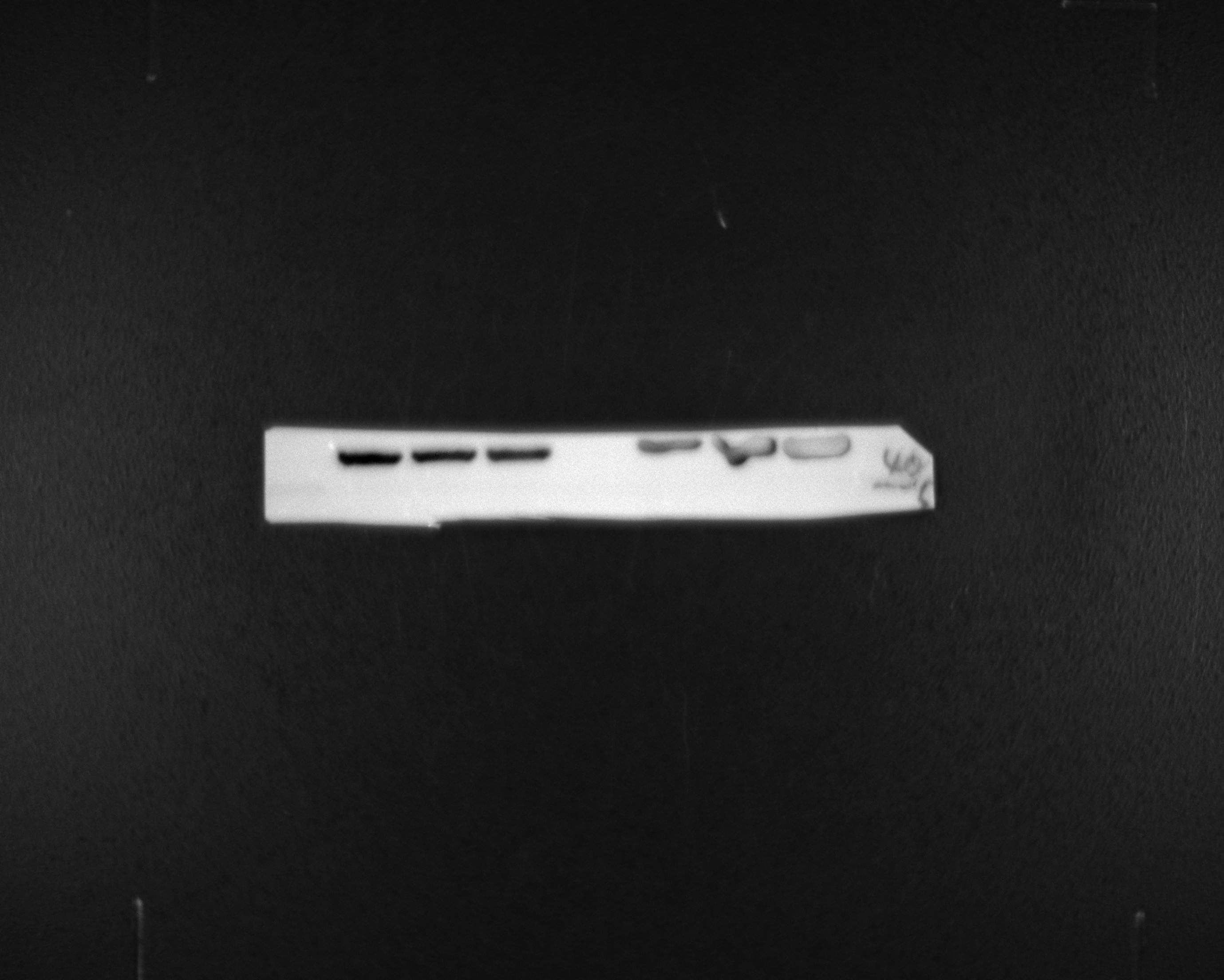

Supplement: Figure 5—source data 2. [file elife-101888-fig5-data2.zip › Figure 5I/Actin.jpg]

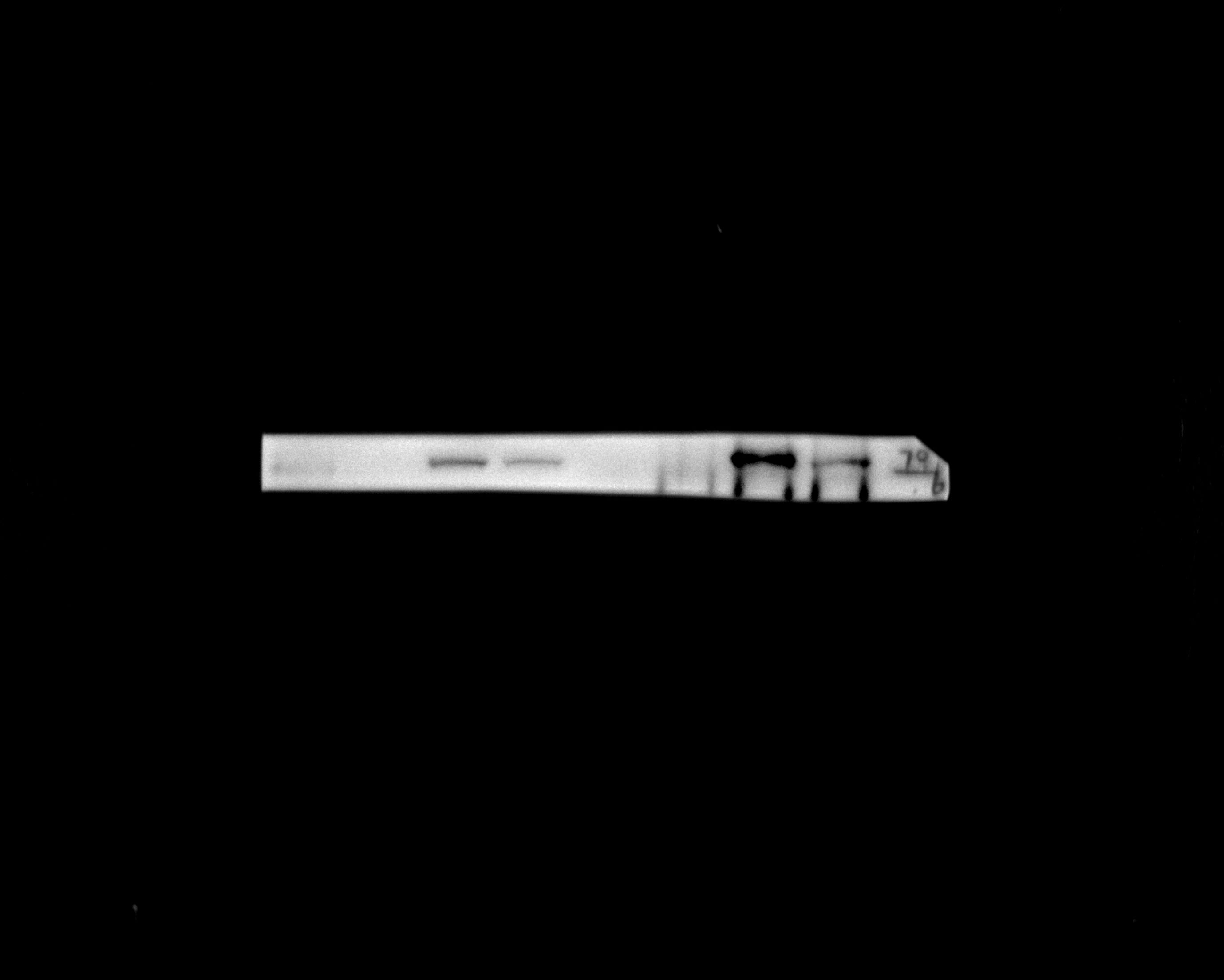

Supplement: Figure 5—source data 2. [file elife-101888-fig5-data2.zip › Figure 5I/Flag.jpg]

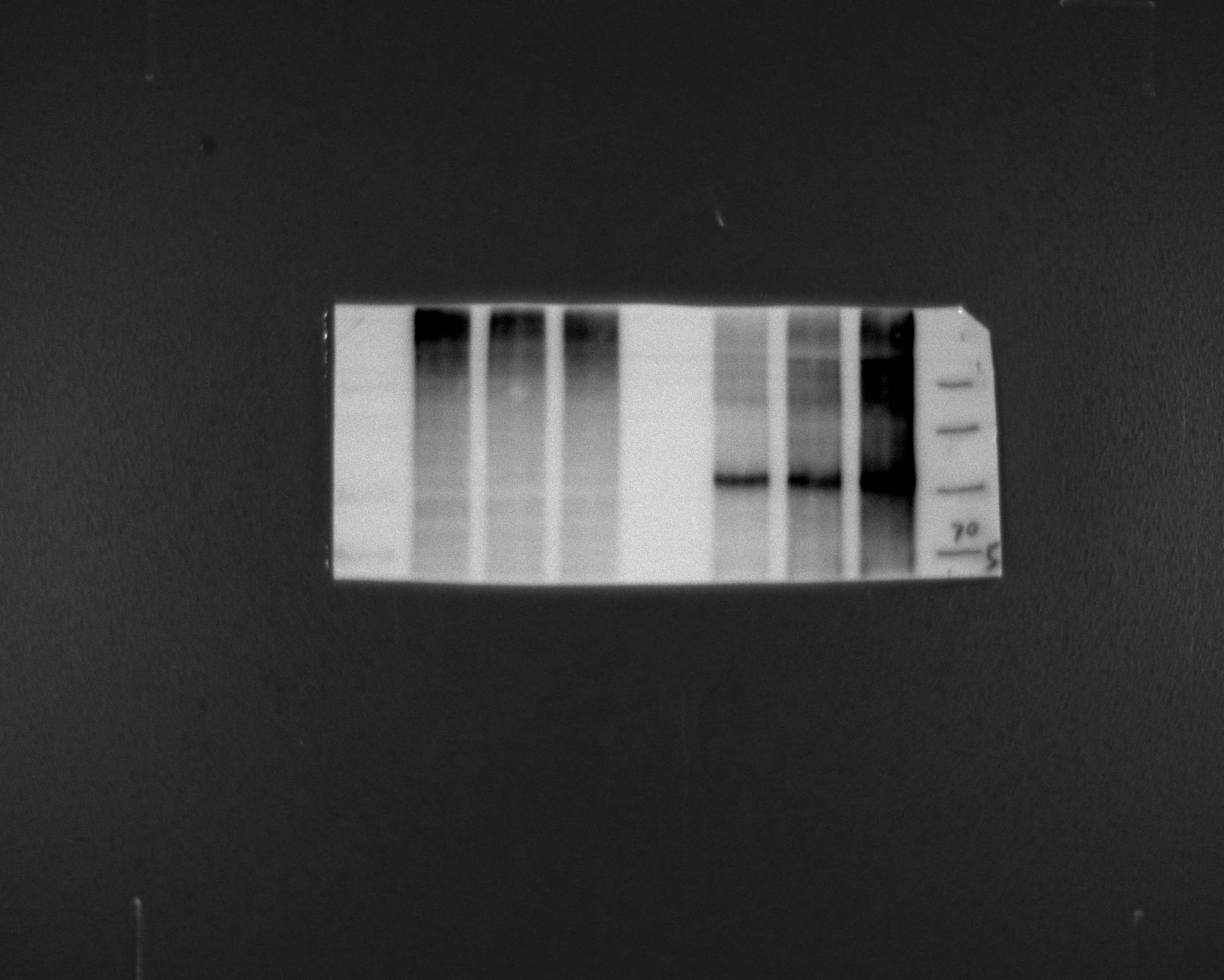

Supplement: Figure 5—source data 2. [file elife-101888-fig5-data2.zip › Figure 5I/Ub.jpg]

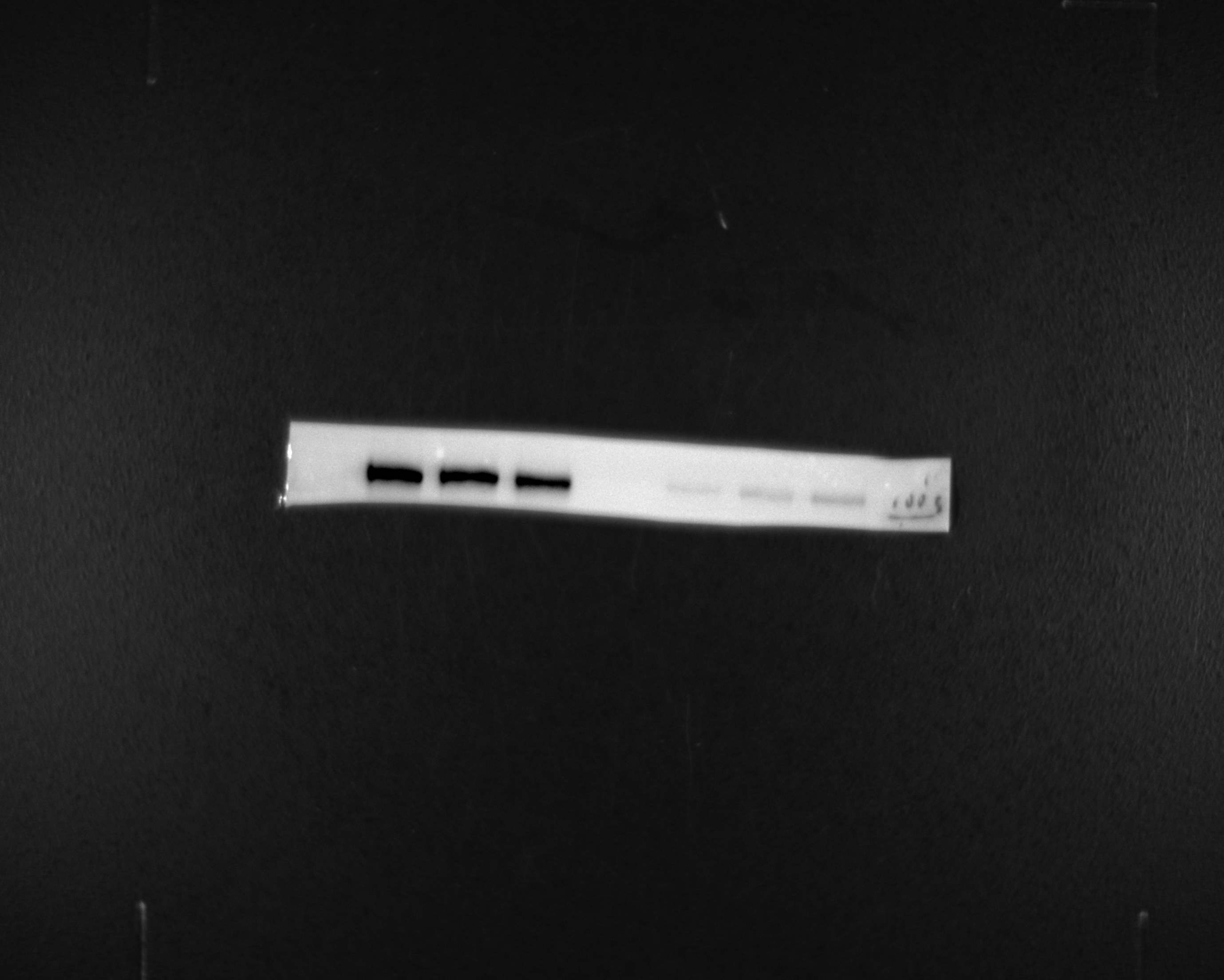

Supplement: Figure 5—source data 2. [file elife-101888-fig5-data2.zip › Figure 5I/UBE3A input.jpg]

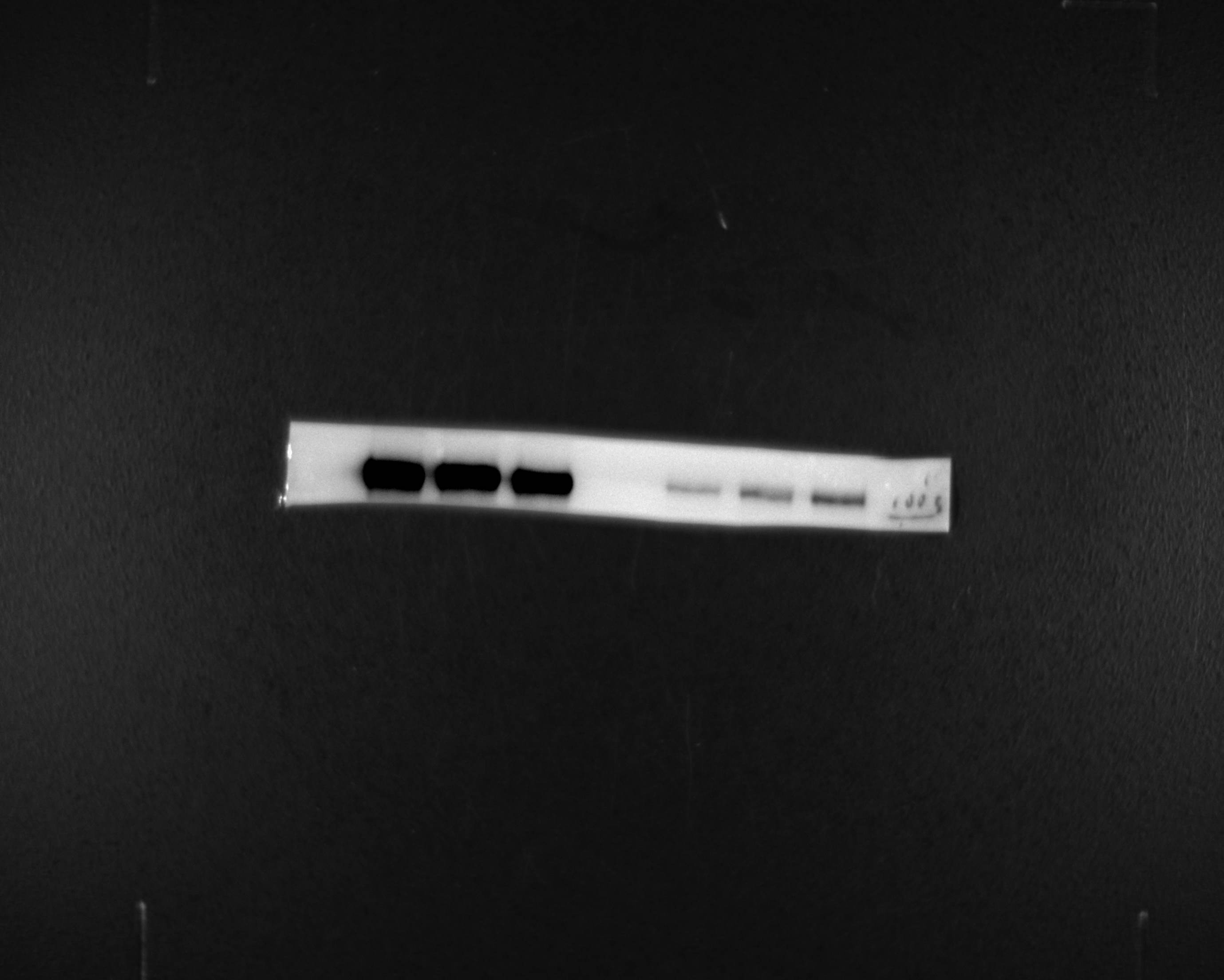

Supplement: Figure 5—source data 2. [file elife-101888-fig5-data2.zip › Figure 5I/UBE3A IP.jpg]

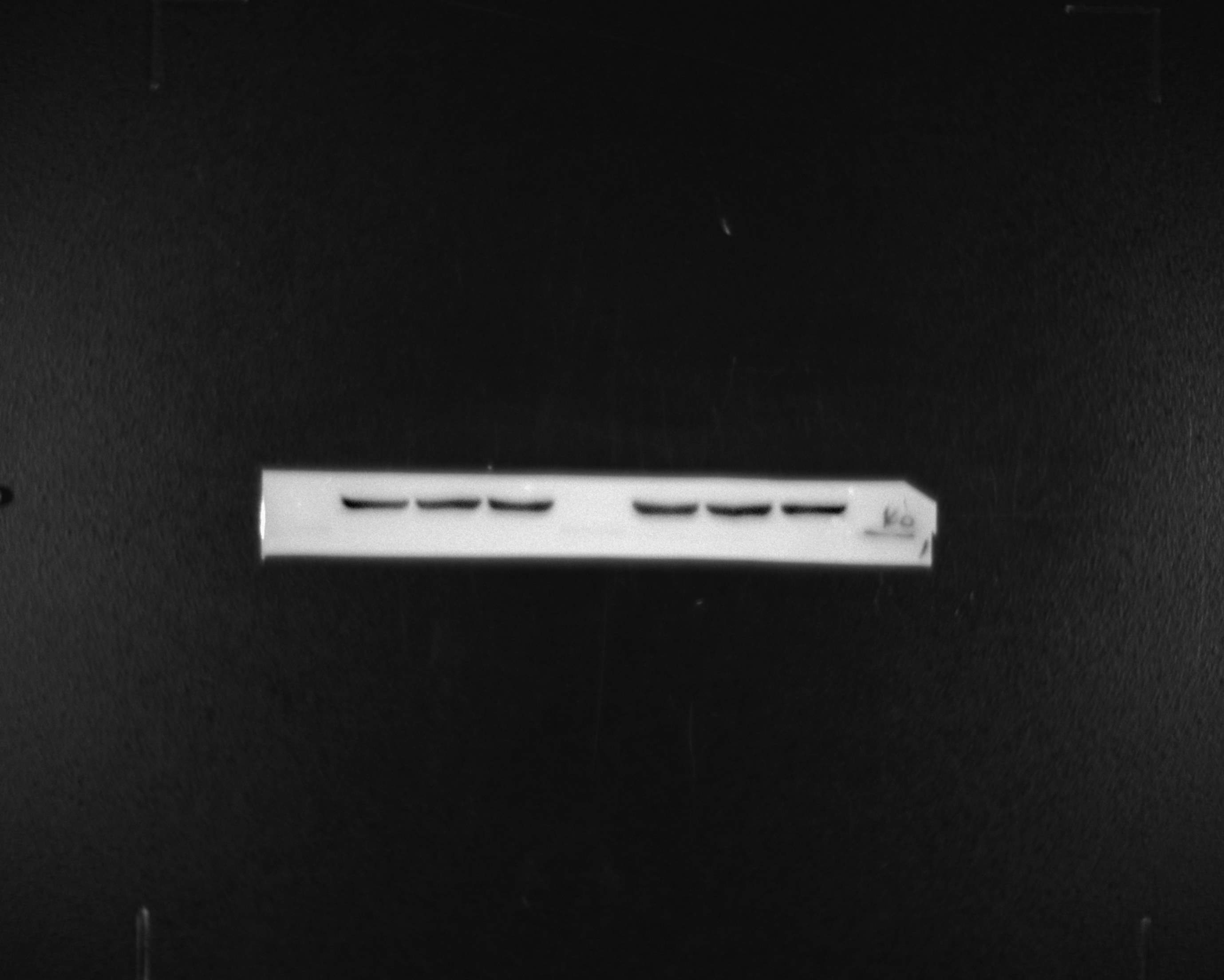

Supplement: Figure 5—source data 2. [file elife-101888-fig5-data2.zip › Figure 5J/Actin.jpg]

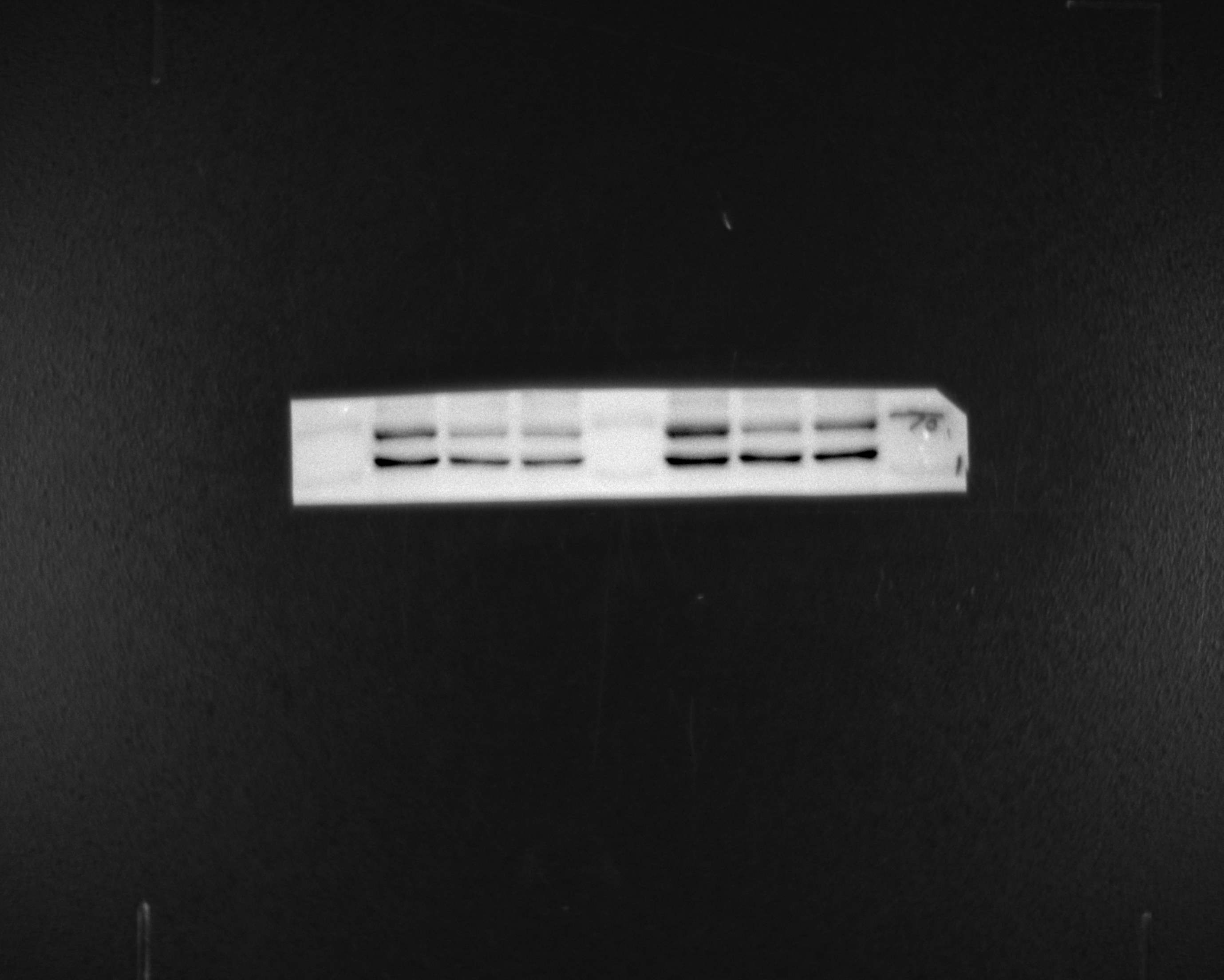

Supplement: Figure 5—source data 2. [file elife-101888-fig5-data2.zip › Figure 5J/ERα.jpg]

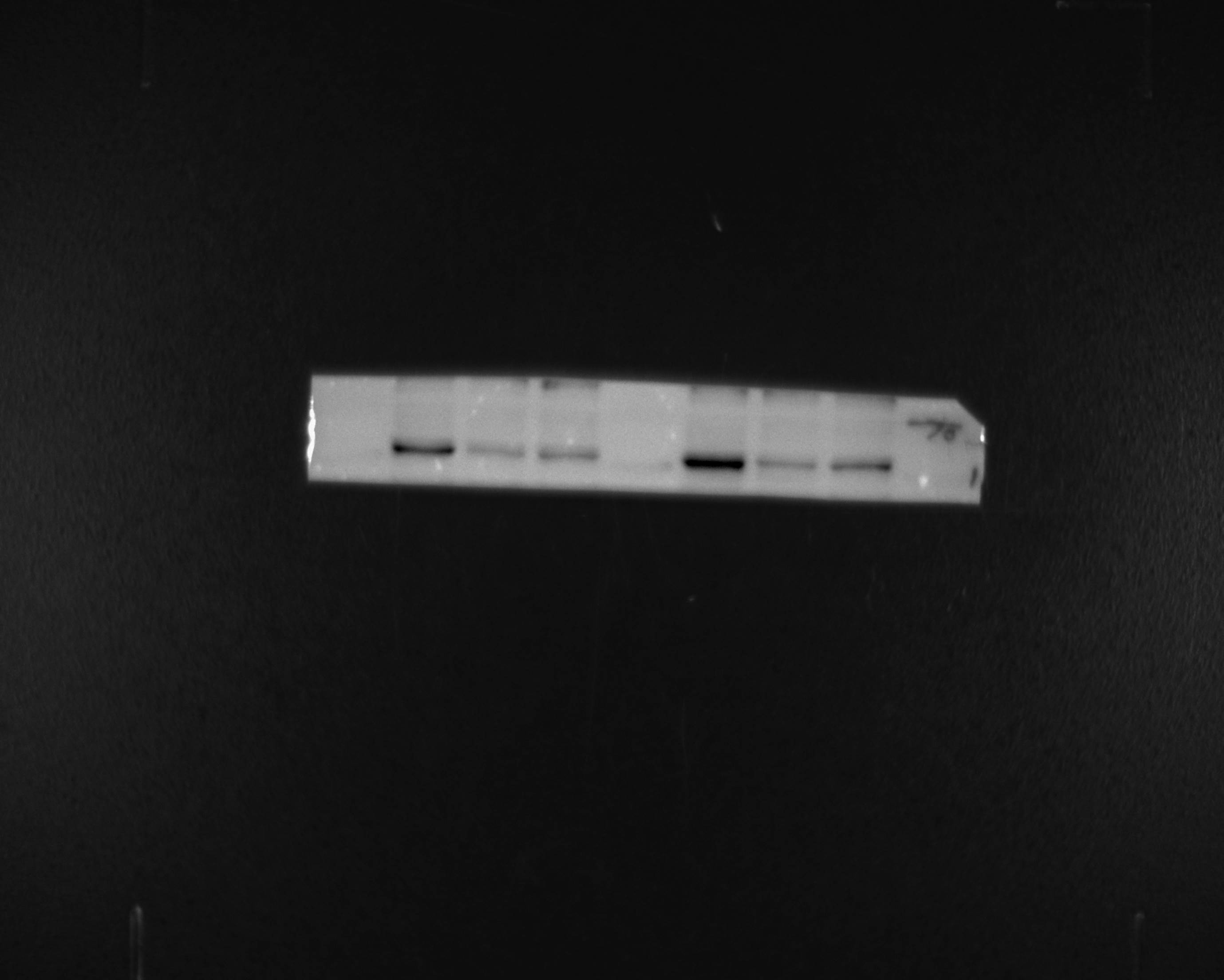

Supplement: Figure 5—source data 2. [file elife-101888-fig5-data2.zip › Figure 5J/FRMD8.jpg]

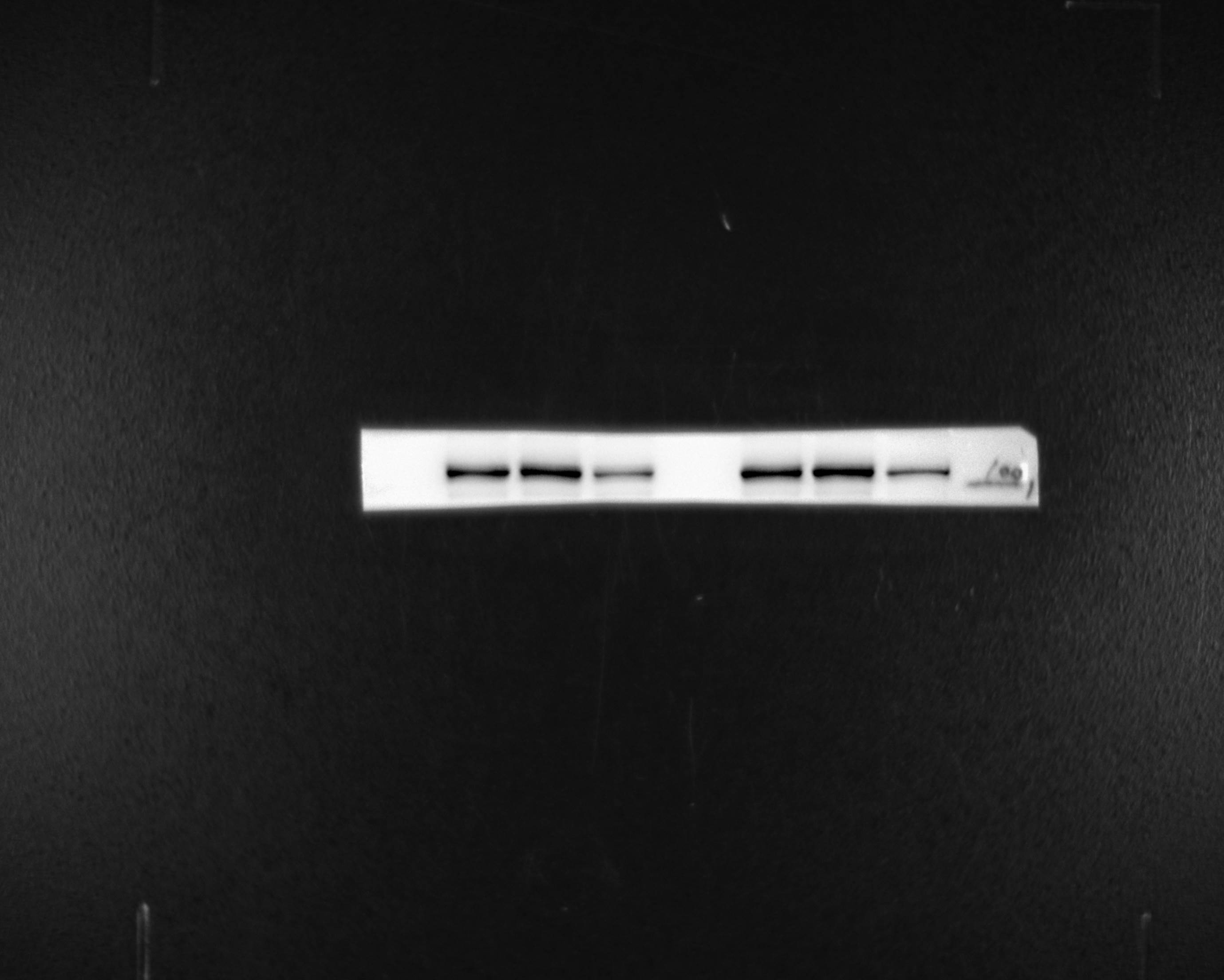

Supplement: Figure 5—source data 2. [file elife-101888-fig5-data2.zip › Figure 5J/UBE3A.jpg]

**Figure 6-source data:** Unedited western blot pictures for figure 6.

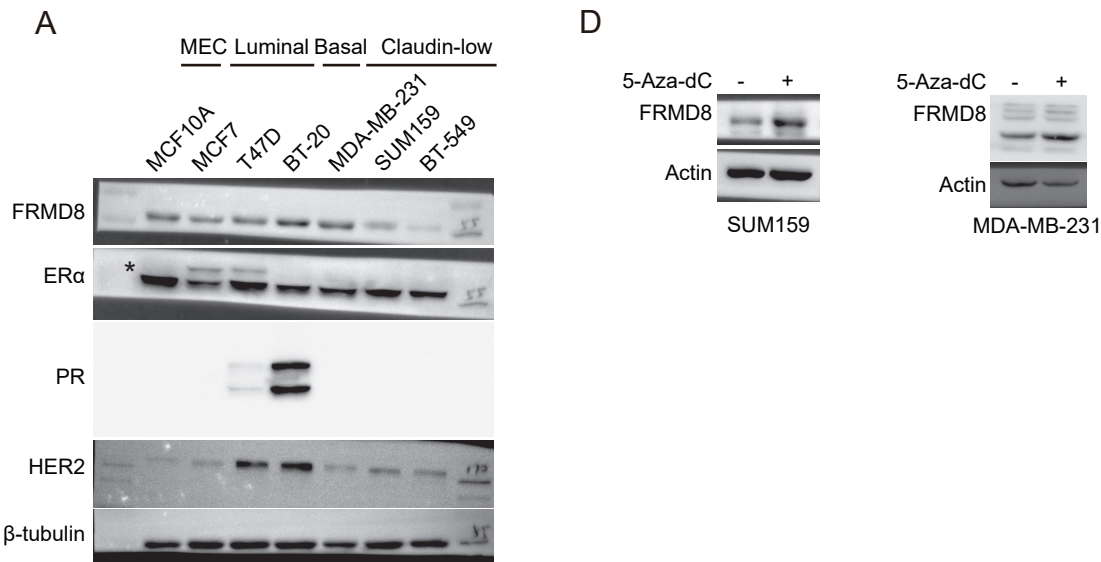

Supplement: Figure 6—source data 1. [file elife-101888-fig6-data1.pdf]

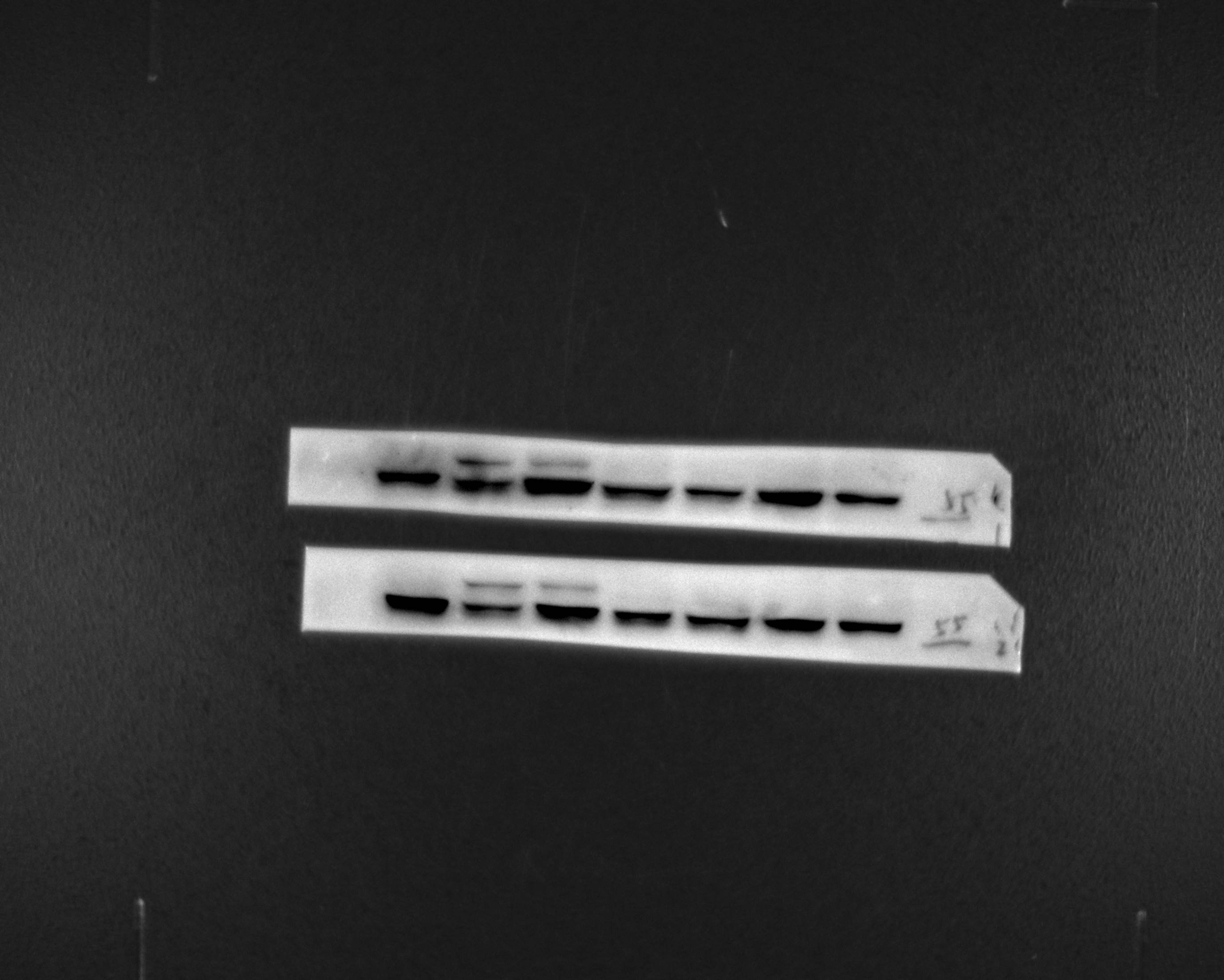

Supplement: Figure 6—source data 2. [file elife-101888-fig6-data2.zip › Figure 6A/ERα.jpg]

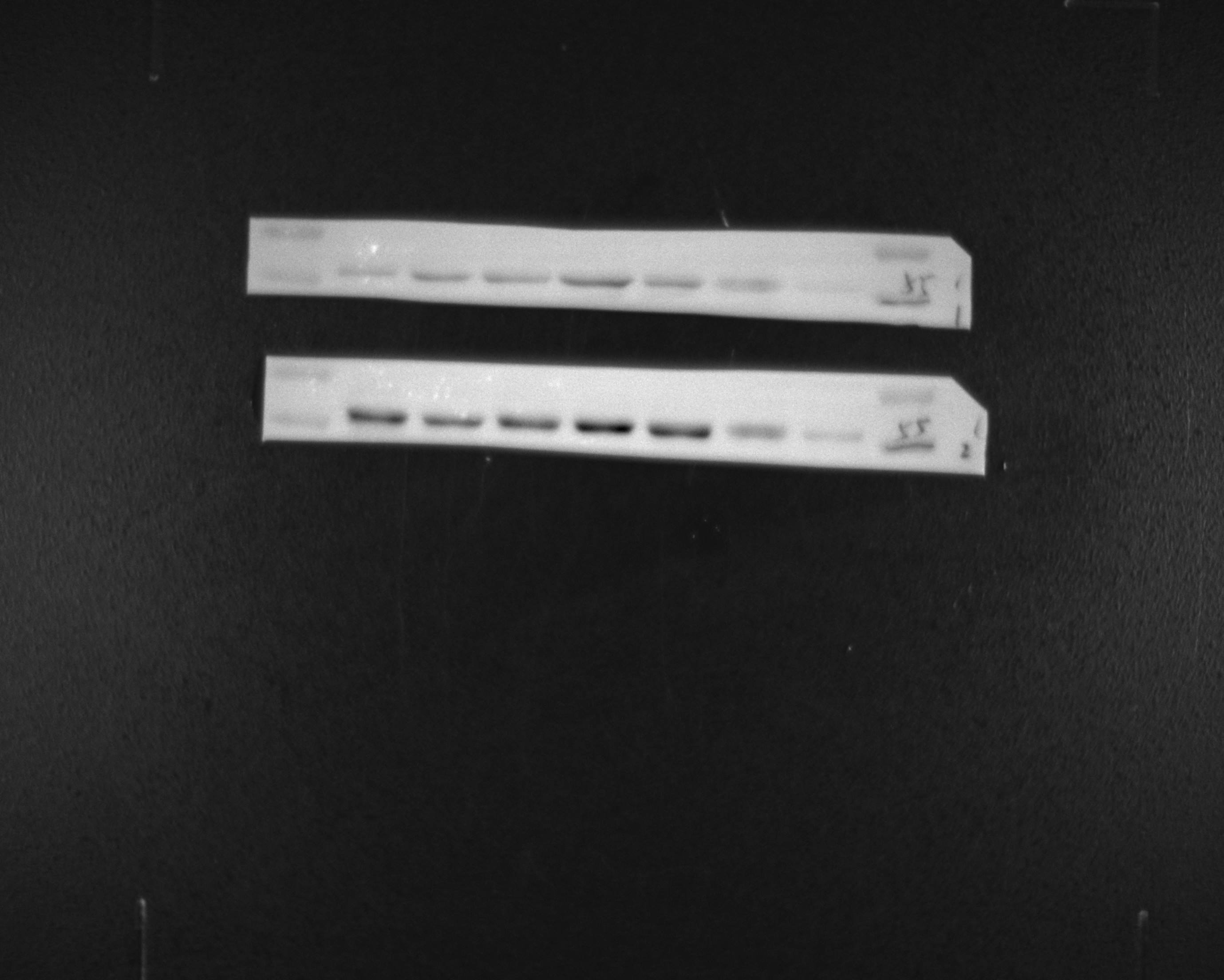

Supplement: Figure 6—source data 2. [file elife-101888-fig6-data2.zip › Figure 6A/FRMD8.jpg]

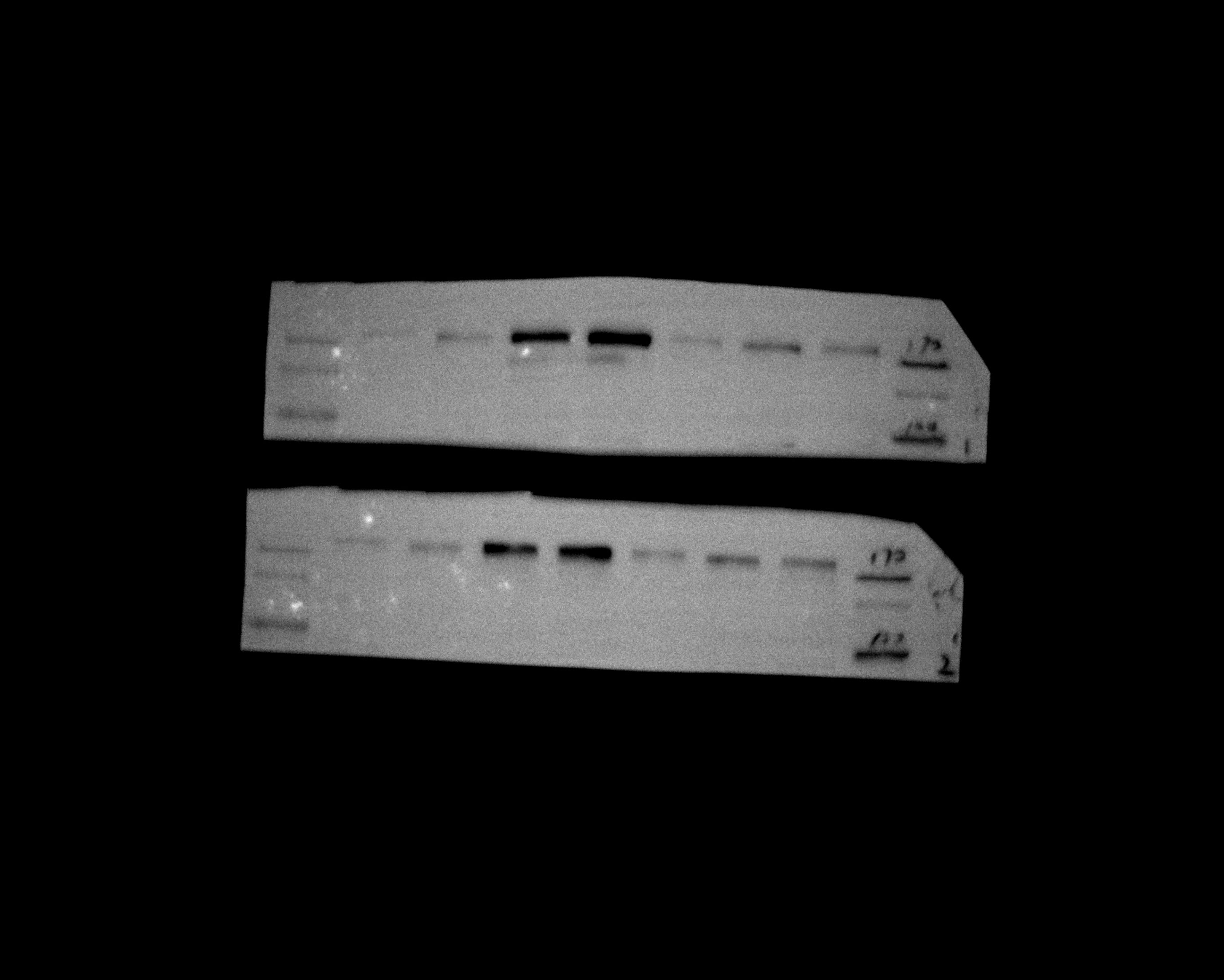

Supplement: Figure 6—source data 2. [file elife-101888-fig6-data2.zip › Figure 6A/HER2.jpg]

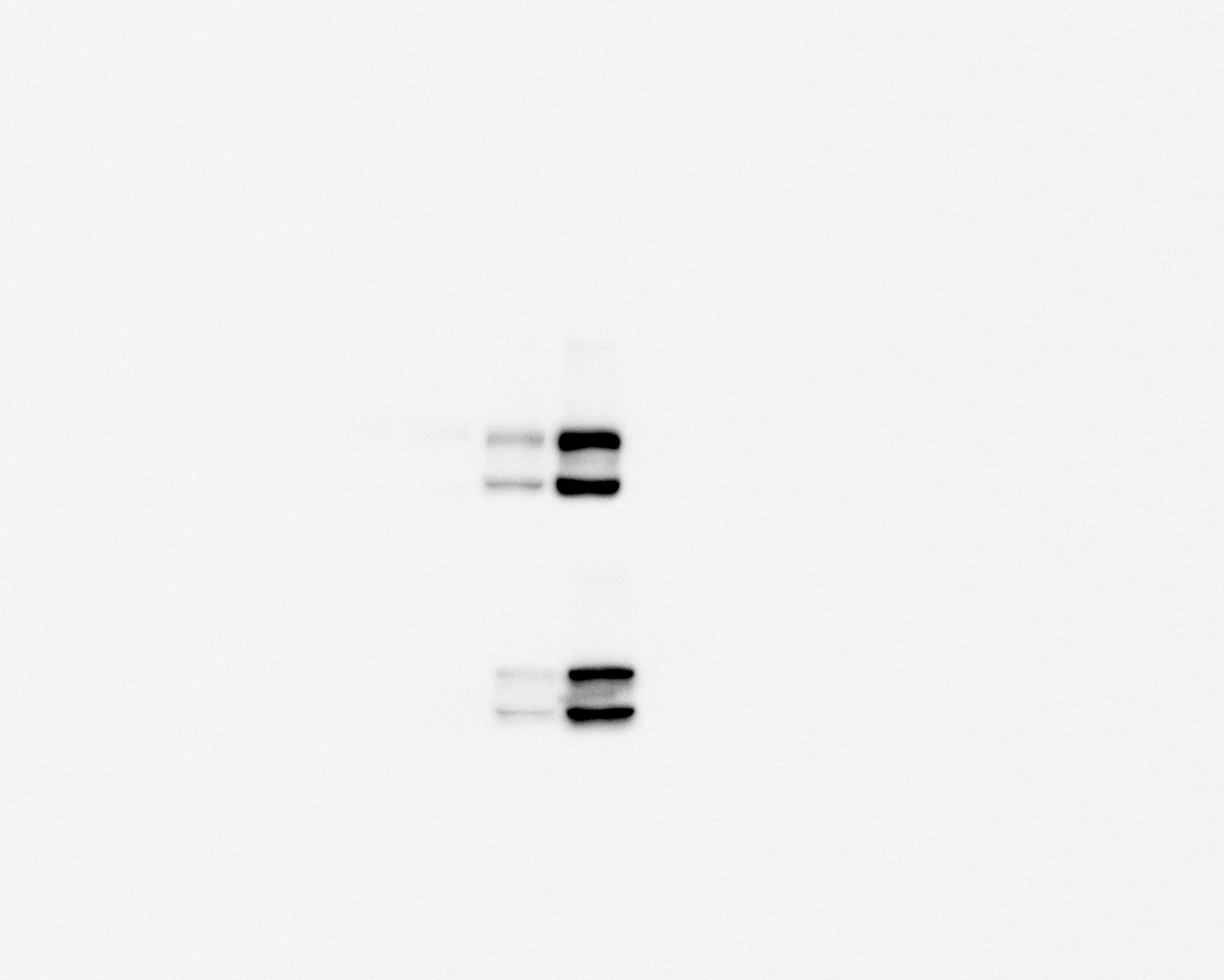

Supplement: Figure 6—source data 2. [file elife-101888-fig6-data2.zip › Figure 6A/PR.jpg]

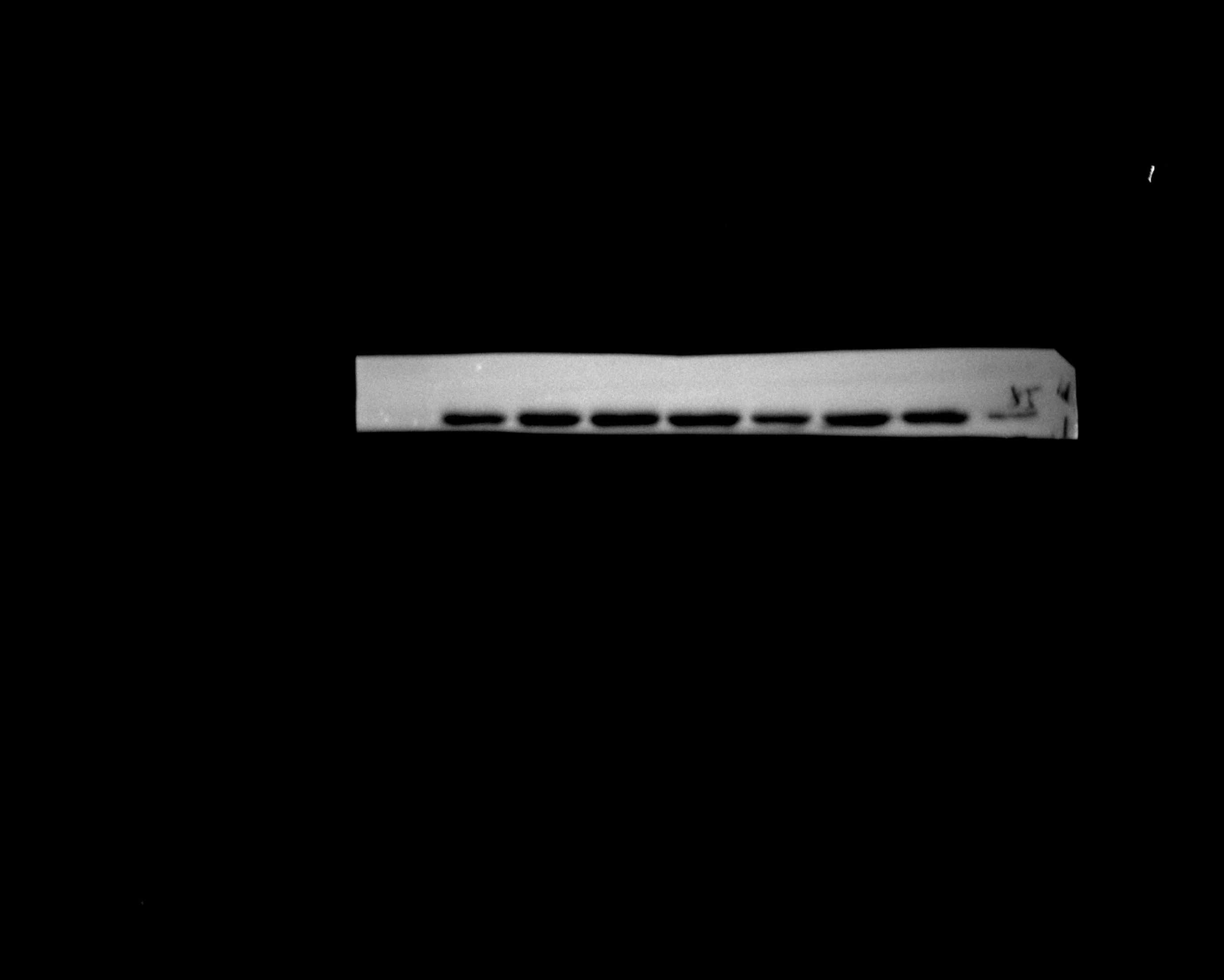

Supplement: Figure 6—source data 2. [file elife-101888-fig6-data2.zip › Figure 6A/β-tubulin.jpg]

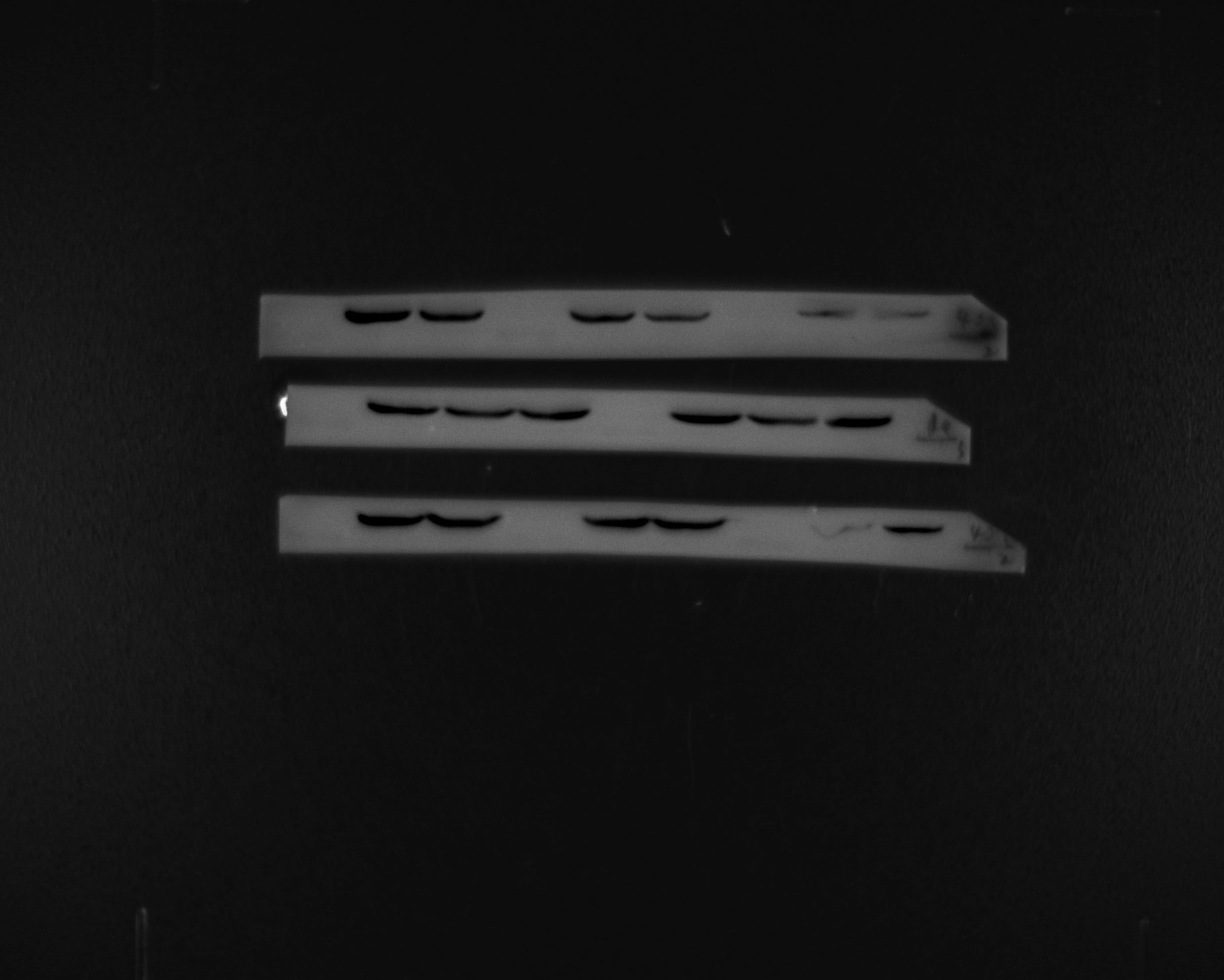

Supplement: Figure 6—source data 2. [file elife-101888-fig6-data2.zip › Figure 6D/Actin MD-MB-231.jpg]

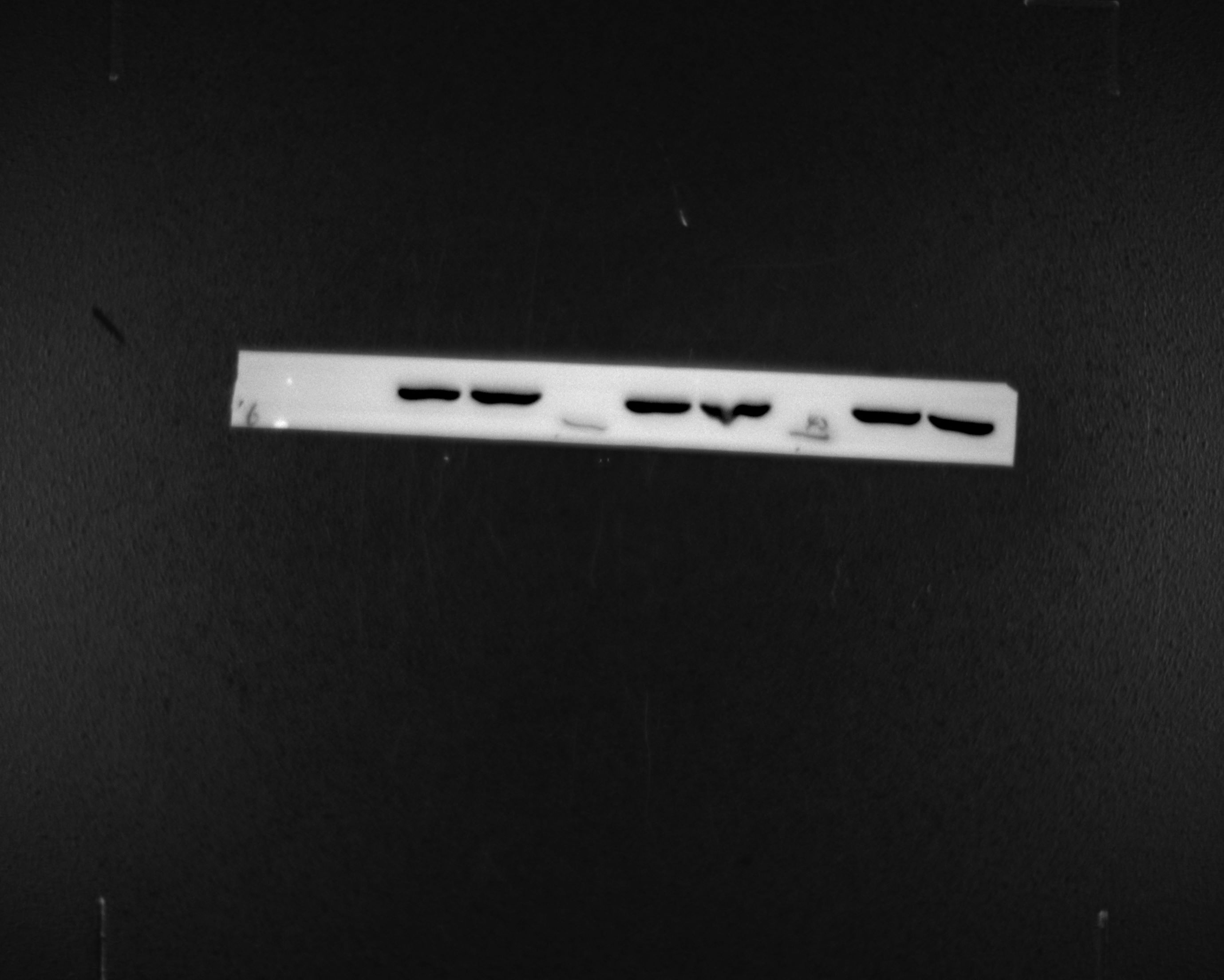

Supplement: Figure 6—source data 2. [file elife-101888-fig6-data2.zip › Figure 6D/Actin SUM159.jpg]

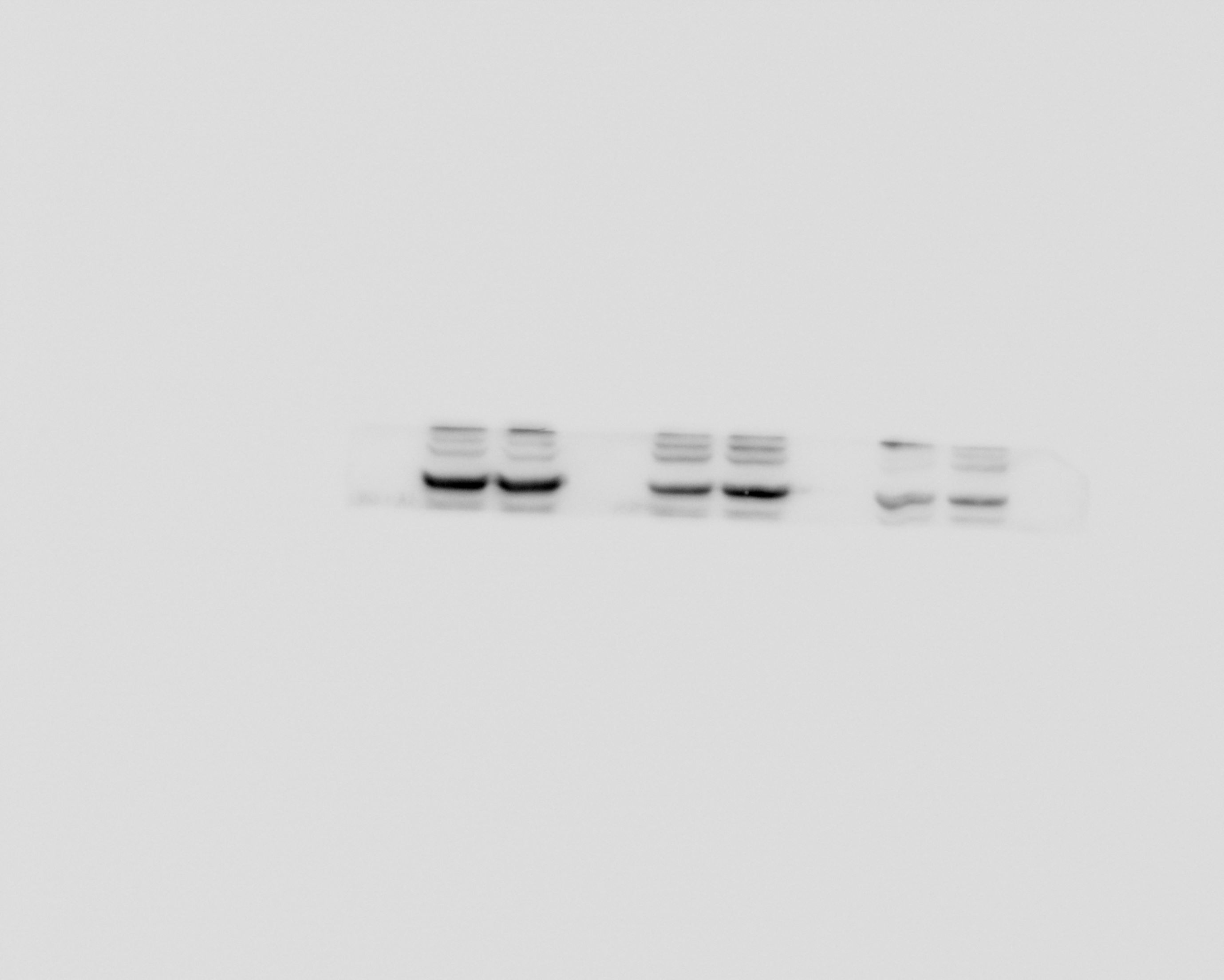

Supplement: Figure 6—source data 2. [file elife-101888-fig6-data2.zip › Figure 6D/FRMD8 MD-MB-231.jpg]

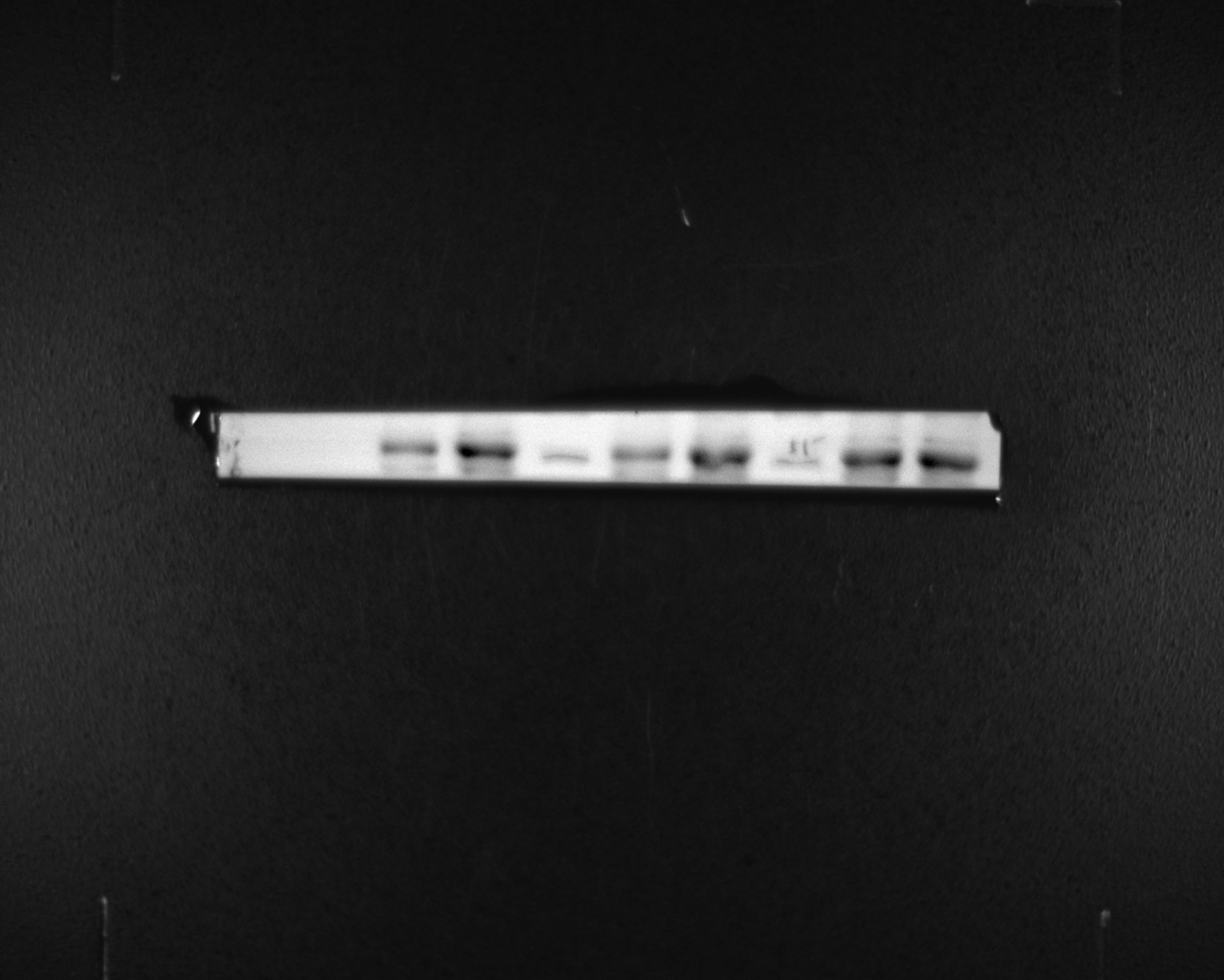

Supplement: Figure 6—source data 2. [file elife-101888-fig6-data2.zip › Figure 6D/FRMD8 SUM159.jpg]

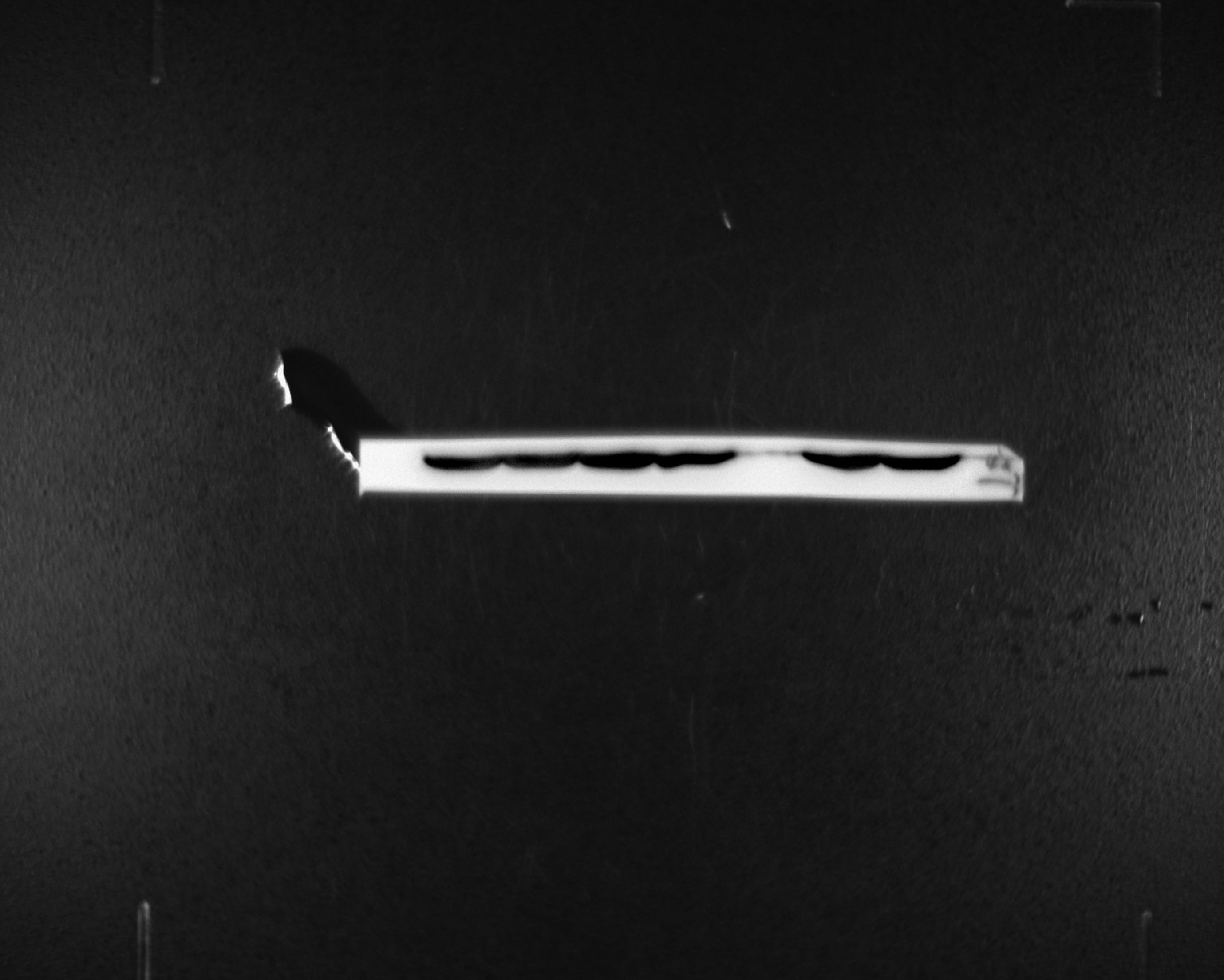

Supplement: Figure 6—figure supplement 1—source data 2. [file elife-101888-fig6-figsupp1-data2.zip › Figure 6– figure supplement 1A/Actin MCF7.jpg]

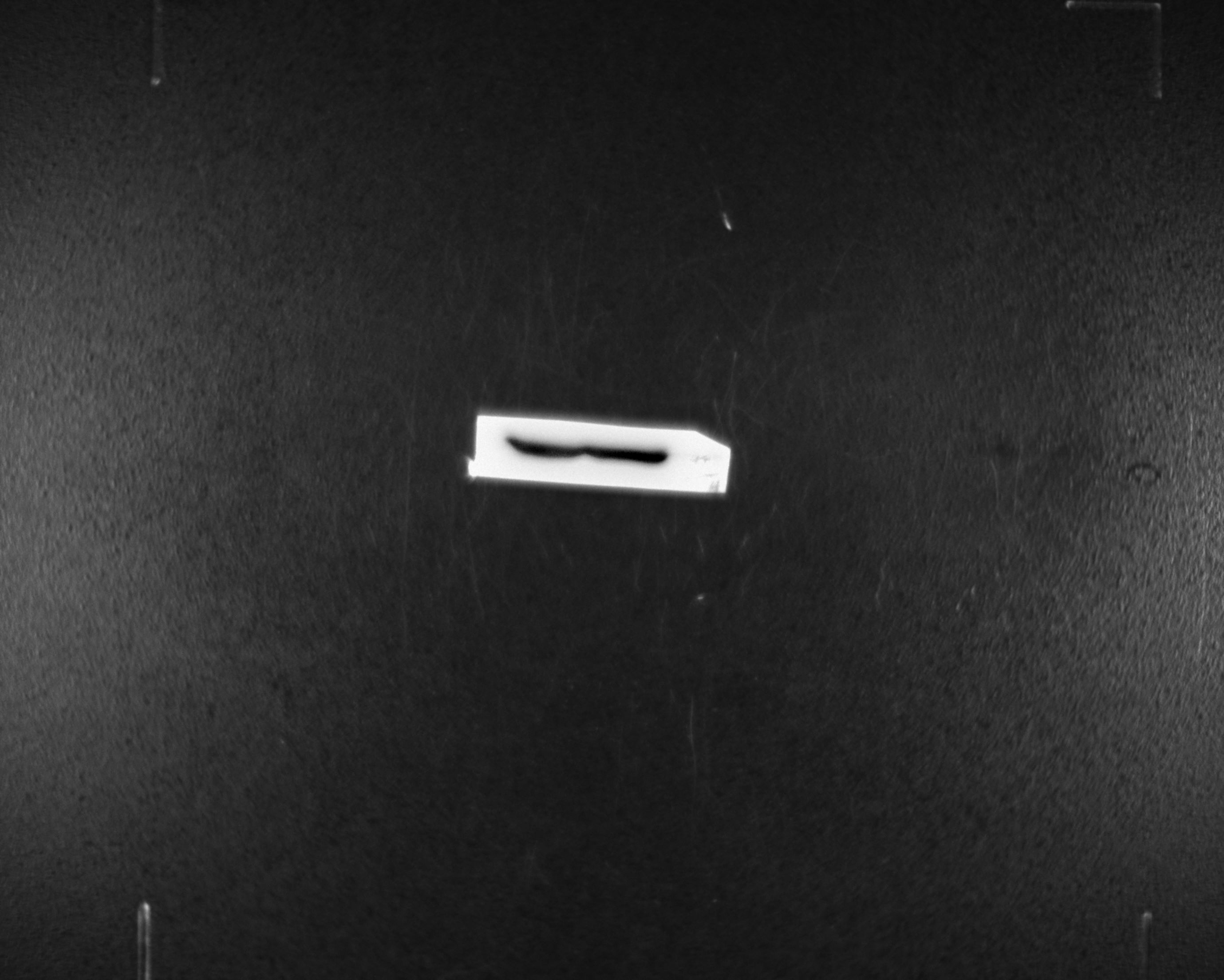

Supplement: Figure 6—figure supplement 1—source data 2. [file elife-101888-fig6-figsupp1-data2.zip › Figure 6– figure supplement 1A/Actin T47D.jpg]

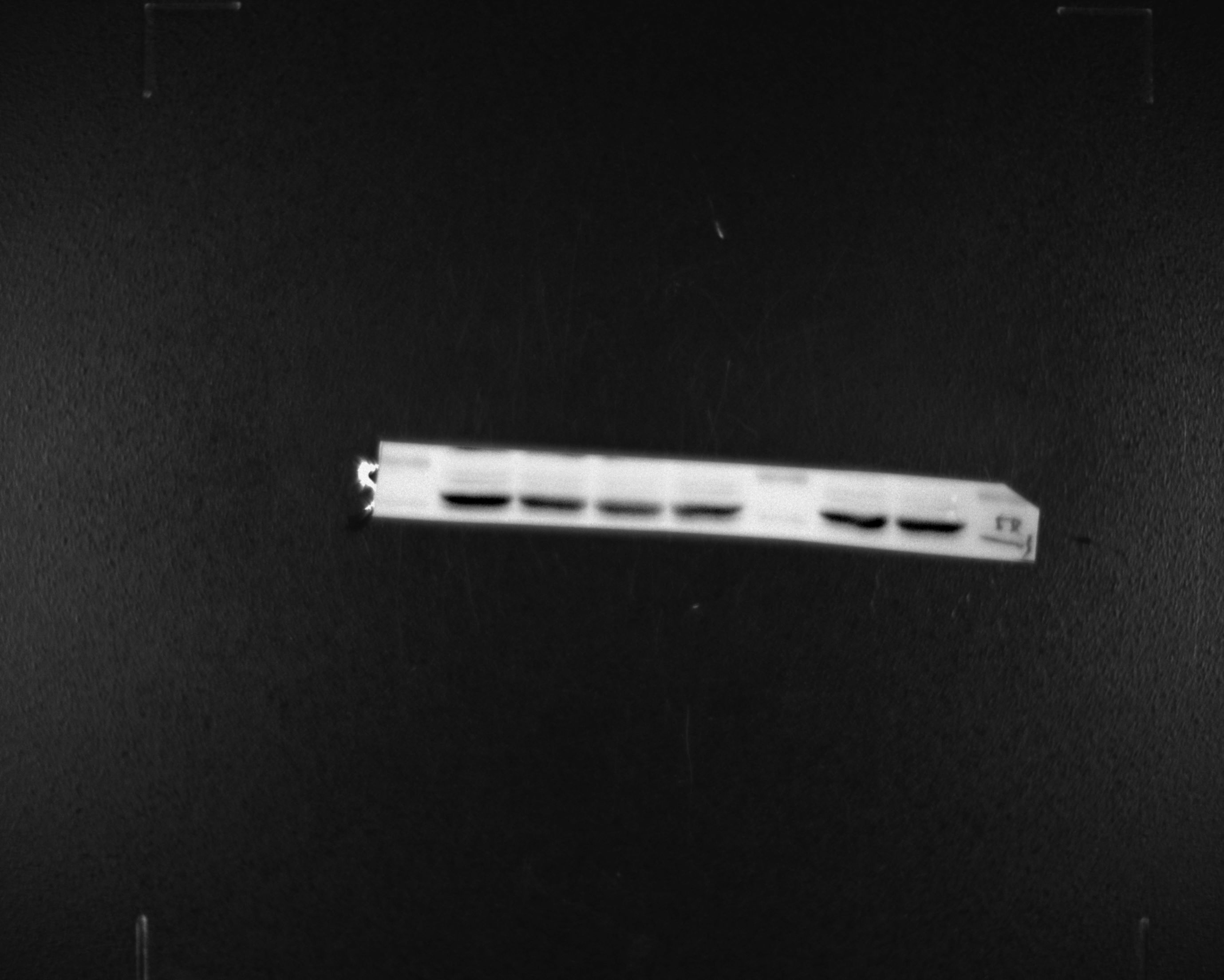

Supplement: Figure 6—figure supplement 1—source data 2. [file elife-101888-fig6-figsupp1-data2.zip › Figure 6– figure supplement 1A/FRMD8 MCF7.jpg]

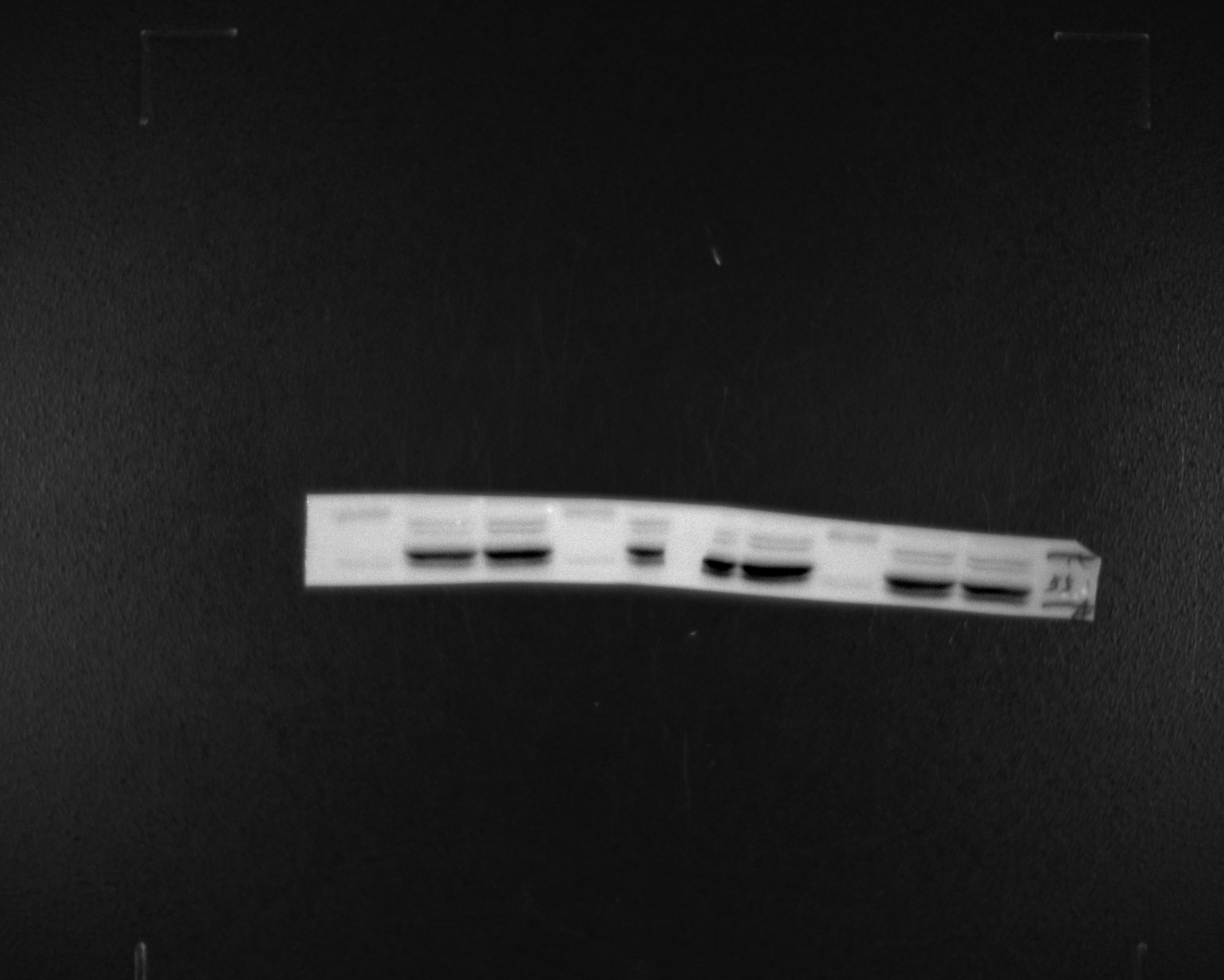

Supplement: Figure 6—figure supplement 1—source data 2. [file elife-101888-fig6-figsupp1-data2.zip › Figure 6– figure supplement 1A/FRMD8 T47D.jpg]
