# Supplementary material for: Loss function of tumor suppressor FRMD8 confers resistance to tamoxifen therapy via a dual mechanism
Source: eLife. 2025 Apr 11;13:RP101888. doi: 10.7554/eLife.101888 (PMC11991697; doi:10.7554/eLife.101888)
Supplement: Supplementary file 2. [file elife-101888-supp2.docx]

# Supplementary file 2. Primers for genotyping, RNA silencing and qRT-PCR

| **Genotyping** |  |  |
| --- | --- | --- |
| **Mice** | **Forward** | **Reverse** |
| *Frmd8* floxed mice | 5’-TGGGTGGAGGTGACAAGGCAAGAA-3’ | 5’-CAGGCTCAGGGTGGTTAGGTCAAT-3’ |
| *MMTV-Cre* transgenic mice | 5’-ATTTGCCTGCATTACCGGTCG-3’ | 5’-CAGCATTGCTGTCACTTGGTC-3’ |
| *MMTV-PyMT* transgenic mice | 5’-GGAAGCAAGTACTTCACAAGGG-3’ | 5’-GGAAAGTCACTAGGAGCAGGG-3’ |
| **siRNA targeted sequence** | | |
| siFRMD8#1 | CCAAGCAGGCCGAACTGAT |  |
| siFRMD8#2 | TGCTCTATGAGGAGGCCAA |  |
| siFOXO3A | CAGCGGAGCTCTAGCTTCCCGTATA |  |
| siUBE3A | CCTACATCTCATACTTGCTTT |  |
| **qRT-PCR** |  |  |
| **Gene** | **Forward** | **Reverse** |
| Mouse *Frmd8* | 5’-CGATGATGATGTCGCCATGG-3’ | 5’-AGTCCTCCAAGTCACAAGGG-3’ |
| Mouse *Gapdh* | 5’-GGGTCCCAGCTTAGGTTCAT-3’ | 5’-CATTCTCGGCCTTGACTGTG-3’ |
| Human *FRMD8* | 5’-TTCTTCCACGGTGAGGTTGA-3’ | 5’-TCGAACTCCAGCCACAAGAT-3’ |
| Human *ESR1* | 5’-ATGTGCCTGGCTAGAGATCC-3’ | 5’-CAAACTCCTCTCCCTGCAGA-3’ |
| Human *GAPDH* | 5’-GCACCACCAACTGCTTAGCA-3’ | 5’-TCTTCTGGGTGGCAGTGATG-3’ |
| **Chip-qPCR** |  |  |
| **Gene** | **Forward** | **Reverse** |
| Human *ESR1* | 5’-CCACTGGGAAATGAGAGACCTCGT-3’ | 5’-GTGGATCAAATGCCTTACTGGCCC-3’ |
